# Lysophosphatidylethanolamine 18:1 drives clear cell renal cell carcinoma by stabilizing SIRT6 to reprogram lipid metabolism

**DOI:** 10.1038/s41392-025-02496-1

**Published:** 2025-12-08

**Authors:** Nanxi Yue, Hongye Zhao, Yong Zhang, Junfei Gu, Jinchun Qi, Jinkun Wen, Wei Wang, Mingming Lv, Hao Sun, Jinsuo Chen, Chenxiao Yang, Changbao Qu, Xiaonan Chen, Zhan Yang

**Affiliations:** 1 Department of Biochemistry and Molecular Biology, The Key Laboratory of Neural and Vascular Biology, Ministry of Education of China, Hebei Medical University, Shijiazhuang, Hebei PR China; 2 Department of Urology, National Cancer Center, National Clinical Research Center for Cancer, Cancer Hospital, Chinese Academy of Medical Sciences and Peking Union Medical College, Beijing, China; 3 Department of Urology, The Second Hospital of Hebei Medical University, Shijiazhuang, China; 4 School of Clinical Medicine, Hebei University, Department of Urology, Affiliated Hospital of Hebei University, Baoding, China; 5 Center of Tumor Immunology and Cytotherapy, Medical Research Center, The Affiliated Hospital of Qingdao University, Qingdao, Shandong China; 6 Department of Urology, Shijiazhuang People’s Hospital, Shijiazhuang, Hebei China; 7 Department of Urology, Shengjing Hospital of China Medical University, Shenyang, PR China

**Keywords:** Cancer metabolism, Cancer microenvironment, Cell biology

## Abstract

Clear cell renal cell carcinoma (ccRCC) is characterized by profound lipid metabolic dysregulation, yet the mechanisms linking peritumoral adipose tissue (PAT)-derived lipid metabolites to tumor aggressiveness remain poorly defined. Here, we identified lysophosphatidylethanolamine 18:1 (LPE18:1), a lipid metabolite enriched in PAT and the arterial blood of ccRCC patients, as a critical driver of tumor growth and lipid deposition. Through multiomics analyses and functional studies, we demonstrated that LPE18:1 upregulates F-actin-capping protein subunit alpha-1 (CAPZA1), which recruits ubiquitin-specific peptidase 48 (USP48) to stabilize the NAD-dependent protein deacetylase sirtuin-6 (SIRT6) by inhibiting its proteasomal degradation. Increased SIRT6 epigenetically promotes acetyl-CoA acetyltransferase 2 (ACAT2) expression, redirecting lipid metabolism toward free cholesterol accumulation—a hallmark of ccRCC aggressiveness. Clinically, CAPZA1 and SIRT6 levels correlate with advanced tumor stage and poor prognosis in ccRCC cohorts. Genetic or pharmacological inhibition of the CAPZA1/SIRT6 axis can reverse LPE18:1-induced lipid deposition and tumor progression in xenograft models. Notably, targeting this axis with the SIRT6 inhibitor OSS-128167 combined with CAPZA1 depletion significantly suppresses ccRCC cell growth. Our study reveals a PAT-derived lipid metabolite-fuelled signaling cascade that reprograms lipid metabolism in ccRCC, identifying CAPZA1/USP48/SIRT6 as actionable therapeutic targets for metabolic malignancies.

## Introduction

Renal cell carcinoma (RCC), particularly its clear cell subtype (ccRCC), is a malignancy characterized by profound metabolic dysregulation, with lipid metabolism reprogramming playing a pivotal role in its tumorigenesis and progression.^1,2^ ccRCC accounts for more than 70% of kidney cancers and is histologically defined by intracellular lipid and glycogen accumulation, underscoring the critical interplay between lipid metabolic reprogramming and the oncogenic growth of ccRCC cells.^3–5^ There were over 430,000 new cases of RCC annually, the global burden of this disease is significant.^6^ Recent research has clarified that the distinctive lipid-laden phenotype of ccRCC is driven by a complex process of metabolic reprogramming.^6^ The altered lipid metabolism is not just a passive feature; it actively drives tumor progression, metastasis, and resistance to anti-cancer therapies, a major challenge in advanced disease.^7,8^ Emerging evidence highlights that perinephric adipose tissue (PAT), a dynamic component of the tumor microenvironment, facilitates ccRCC progression through bidirectional crosstalk with ccRCC tumors.^9^ For example, PAT undergoes thermogenic “browning”, releasing massive amounts of lactate as a “fuel” to power tumor growth, invasion, and therapy resistance.^10^ On the other hand, ccRCC cells secrete some factors that actively remodel PAT. Parathyroid hormone-related protein (PTHrP) secreted by ccRCC cells acts on the PAT, triggering a process whereby the white, energy-storing fat transforms into brown, energy-burning fat, which in turn fuels ccRCC progression.^10^ These suggest that the PAT and ccRCC form a mutually reinforcing partnership—the tumor directs the fat to change, and the changed fat, in turn, provides the “fuel” the tumor needs to thrive. Additionally, lipidomic dysregulation driven by epigenetic alterations, such as the deficiency of SETD2, a histone methyltransferase that functions as a critical tumor suppressor, accelerates sphingomyelin synthesis and accumulation, facilitating ccRCC development.^11^ Despite these advances, the mechanisms linking PAT-derived lipid metabolites to ccRCC progression remain poorly defined. Elucidating how lipid intermediates from PAT influence tumor metabolism and aggressiveness is essential for identifying therapeutic vulnerabilities in this recalcitrant malignancy.

Small lipid metabolites, including lysophospholipids, have emerged as key regulators of cancer cell behavior.^12^ Lysophosphatidylethanolamine (LPE), a hydrolytic product of phosphatidylethanolamine, is increasingly recognized as an important modulator of cellular functions by modulating the membrane dynamics and signaling cascades.^13^ Studies have shown that LPE regulates the chemotactic migration and invasion of ovarian cancer cells through the pertussis toxin-sensitive G protein-coupled receptor signaling pathway.^14^ Phospholipase A (PLA), primarily the PLA2 subtype, hydrolyzes membrane phospholipids to produce LPE, and the latter incorporates into the membrane bilayer, disrupting the packing of lipids, increasing membrane fluidity, and impairing TNFR1-mediated p38 signaling,^15^ all of which create a cellular environment that promotes cancer development and progression. Whole-cell and plasma membrane lipidomic analysis of adipogenesis showed that changes in LPE and other lipids in cells are closely related to adipogenic differentiation.^16^ The lipoprotein-associated phospholipase A2 (Lp-PLA2) inhibitor darapladib remodels lipid metabolism by reducing LPE species, thereby sensitizing cancer cells to ferroptosis.^17^ These studies clearly suggest that LPE exerts the crucial roles in the regulation of lipid metabolism and adipogenesis and is implicated in tumorigenesis and progression. However, the role of LPE, particularly its specific species and downstream effectors, in ccRCC is unclear.

Capping actin protein of muscle Z-line subunit alpha 1 (CAPZA1), a component of the F-actin capping machinery, regulates cytoskeletal dynamics and cell motility.^18^ In addition to its structural role, CAPZA1 modulates signal transduction by interacting with multiple kinases and phosphatases.^19,20^ In cancer, CAPZA1 plays context-dependent roles: it suppresses metastasis in hepatocellular carcinoma (HCC) by inhibiting EMT through the downregulation of Snail1 and ZEB1,^21^ whereas in gastric cancer, CAPZA1 overexpression stabilizes *Helicobacter pylori*-induced oncogenic cytotoxin-associated gene A (CagA), promoting CD44v9-positive cancer stem-like cell formation.^22,23^ Similarly, CAPZA1 phosphorylation enhances prostate cancer cell adhesion and migration.^24^ This functional dichotomy across cancer types suggests that the role of CAPZA1 is heavily influenced by tissue-specific signaling contexts and microenvironmental factors. Notably, bioinformatic analyses of ccRCC datasets indicate that CAPZA1 overexpression is not only an indicator of poor prognosis but also correlates clinically with advanced disease stage and reduced survival, positioning it as a potential biomarker.^25^ However, the functional significance of this observation and the mechanistic basis underlying the role of CAPZA1 in ccRCC pathogenesis remain completely unexplored. Specifically, the potential connection between CAPZA1 and lipid metabolic dysregulation characteristic of ccRCC represents a significant knowledge gap in the field of study. Given that metabolic and cytoskeletal dysregulation could be taken as a hallmark of ccRCC, CAPZA1 may influence lipid metabolic dysregulation-driven tumor progression or metastatic potential.

This study identified LPE18:1 as a PAT-derived metabolite that promotes ccRCC progression via CAPZA1-mediated stabilization of SIRT6, which facilitates ACAT2 expression to reprogram lipid metabolism. SIRT6 regulates diverse biological processes including DNA repair, inflammation, and lipid metabolism, while ACAT2 is a key cholesterol esterification enzyme that facilitates lipid droplet formation, a hallmark of ccRCC.^26^ While SIRT6 is known to function as a tumor suppressor in some cancers, recent evidence suggests it can act as a tumor promoter in specific contexts, such as in intrahepatic cholangiocarcinoma (iCCA) where it promotes glutamine metabolic reprogramming.^27^ Our finding reveals a novel signaling axis linking the SIRT6-mediated epigenetic regulation to lipid metabolic reprogramming, offering mechanistic insights into how PAT drives ccRCC aggressiveness and identifying CAPZA1/SIRT6 as potential therapeutic targets for the management of ccRCC and other related conditions.

## Results

### Perirenal adipose tissue browning promotes LPE18:1 production and consumption by ccRCC tumors

**Fig. 1:**
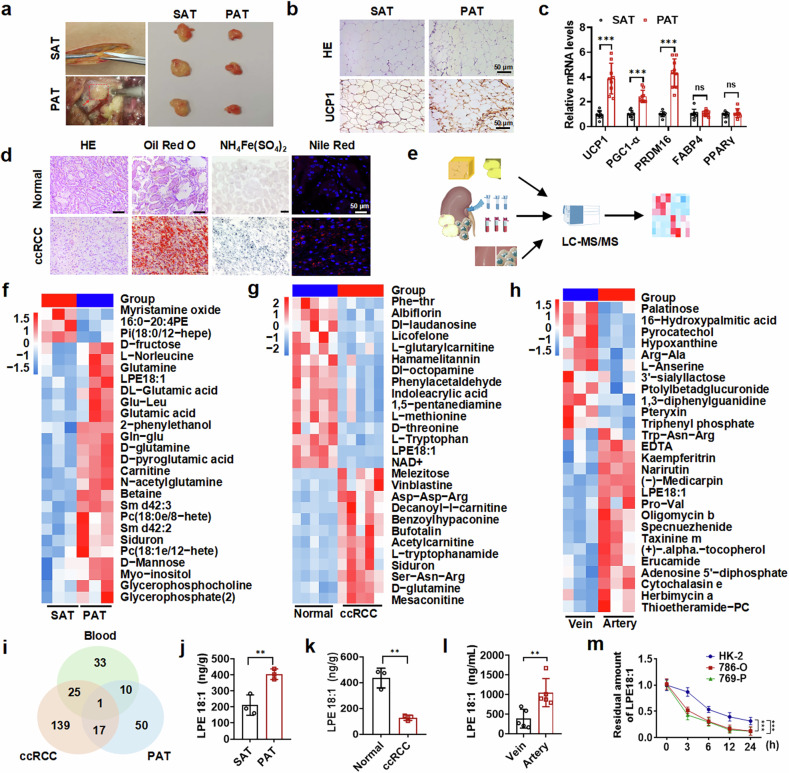
Browning of perirenal adipose tissue enhances LPE18:1 production and tumor consumption in ccRCC. **a** Schematic of perinephric (PAT) and subcutaneous (SAT) adipose tissue sampling in ccRCC patients. **b** H&E and IHC staining of UCP1 in PAT and SAT. Scale bar = 50 μm. **c** mRNA expression of browning markers (UCP1, PGC1-α, and PRDM16) and adipocyte markers (FABP4 and PPARγ) in PAT vs. SAT. **d** Histopathological analysis of normal kidney and ccRCC tissues via H&E, Oil Red O, and Prussian blue iron staining and Nile Red staining. Scale bar = 50 μm. **e** Workflow for metabolomic profiling of PAT and ccRCC tissues and paired arterial/venous blood. Heatmaps of differentially abundant metabolites in PAT vs. SAT (**f**), tumor vs. normal tissue (**g**), and venous vs. arterial blood (**h**). Red/blue indicates up/downregulation (fold change >2, P < 0.05). **i** Venn diagram of common dysregulated metabolites across PAT, tumor, and venous blood. HPLC quantification of LPE18:1 in PAT vs. SAT (**j**), arterial vs. venous blood (**k**), and ccRCC vs. normal kidney tissues (**l**). **m** LPE18:1 consumption assay in HK-2, 786-O, and 769-P cells over 24 hours. The data represent the means ± SDs (n ≥ 3 per group). **P < 0.01, ***P < 0.001

Previous studies have shown that PAT plays a vital role in the progression of ccRCC.^10^ Morphologically, we found that PAT tissue was brown and stiffer in texture than the distal subcutaneous adipose tissue (SAT) of ccRCC patients (Fig. 1a). Importantly, PAT cells in ccRCC patients vary in shape and size. Moreover, the expression of uncoupling protein 1 (UCP1), a mitochondrial endomembrane transporter responsible for thermogenesis, was greater in PAT cells than in SAT cells (Fig. 1b). Further gene expression analysis revealed that the expression of marker genes associated with adipocyte browning, including UCP1, PGC1-ɑ, and PRDM16, was greater in PAT than in SAT (Fig. 1c), confirming that the PAT attached to the ccRCCs may undergo browning.

Lipid metabolic reprogramming and lipid droplet deposition are the main characteristics of ccRCC.^11^ Tissue staining of the collected clinical samples, including Oil Red O, NH4Fe(SO4)2 and Nile Red staining, revealed the deposition of large lipid droplets in the ccRCC cells (Fig. 1d). To investigate whether and how browning of perirenal fat affects the development of ccRCC, we performed metabolomic analyses of PAT tissue, ccRCC tissue, and arterial and venous blood flowing through the kidney (Fig. 1e). Mass spectrometry analysis revealed significant differences in various metabolic molecules among tumor tissues, adipose tissues, and blood flowing through the kidney. Notably, the levels of lysophosphatidylethanolamine 18:1 (LPE18:1) were significantly elevated in PAT and renal arterial blood but markedly decreased in ccRCC tissues (Fig. 1f–i, Supplementary Fig. 1a–c and Supplementary Tables 1–3). Furthermore, high-performance liquid chromatography (HPLC) analysis confirmed that the levels of LPE18:1 were higher in PAT than in SAT, as well as in renal arterial blood than in venous blood, and lower in ccRCC tissue than in normal renal tissue (Fig. 1j–l). Interestingly, when equal amounts of LPE18:1 were added to normal renal epithelial cells and renal cancer cells, tumor cells consumed LPE18:1 at a significantly faster rate than did normal cells (Fig. 1m). These results suggest that LPE18:1, particularly derived from perinephric adipose tissue or peripheral blood, may contribute to the pathogenesis of ccRCC.

### LPE18:1 promotes ccRCC cell proliferation by enhancing lipid deposition and mitochondrial energy production

**Fig. 2:**
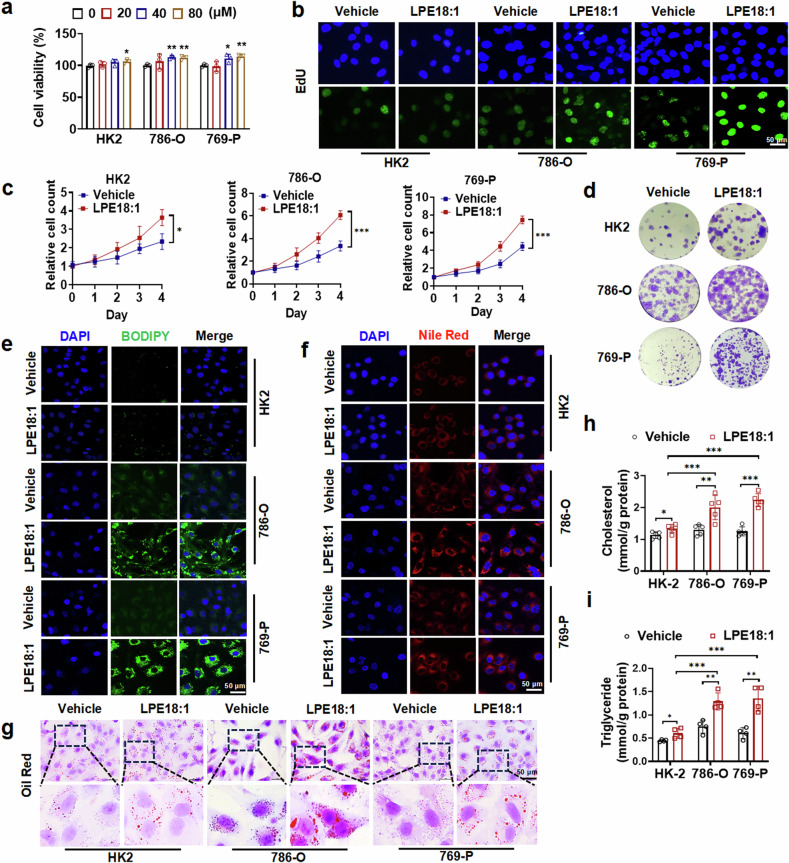
LPE18:1 promotes ccRCC proliferation and lipid accumulation. **a** Viability of HK2, 786-O and 769-P cells treated with LPE18:1 (0–80 μM) for 24 h, as assessed by a CCK-8 assay. **b** An EdU assay was used to detect the proliferative activity of HK-2, 786-O, and 769-P cells after LPE18:1 treatment (40 μM, 24 h). Scale bars = 50 μm. **c** Growth curves of HK-2, 786-O, and 769-P cells under LPE18:1 stimulation (40 μM) over 96 h. **d** Colony formation assay showing long-term proliferative effects of LPE18:1. Lipid droplet accumulation visualized via BODIPY 493/503 (**e**), Nile Red (**f**), and Oil Red O (**g**) staining in LPE18:1-treated cells. Nuclei were counterstained with DAPI. Scale bars = 50 μm. Intracellular triglyceride (**h**) and total cholesterol (**i**) levels in HK-2, 786-O, and 769-P cells following LPE18:1 treatment. The data represent the means ± SDs (n ≥ 3 per group). *P < 0.05, **P < 0.01, ***P < 0.001 versus untreated controls

Recent studies have indicated that LPEs promote cell proliferation and participate in lipid synthesis.^28,29^ To investigate the role of LPE18:1 in ccRCC, we first examined its regulatory effect on cell proliferation-related genes across different cell lines. Compared with the normal renal cell line HK2, LPE18:1 had a more significant pro-proliferative effect on 786-O and 769-P cells (Supplementary Fig. 2a, b). Therefore, we focused on these two cell lines for subsequent functional studies. We performed a CCK-8 assay to evaluate the effect of LPE18:1 on cell viability. The results demonstrated that LPE18:1 promoted ccRCC cell proliferation in a dose-dependent manner, with 40 μM showing the most pronounced effect (Fig. 2a). This concentration was thus selected for subsequent experiments. Furthermore, we carried out an EdU assay, a cell counting assay, and a colony formation assay to examine the effects of LPE18:1 on cell growth. The results showed that LPE18:1 promoted cell proliferation, with a more pronounced pro-proliferative effect on 786-O and 769-P cells than on HK2 cells (Fig. 2b–d and Supplementary Fig. 2c, d). Moreover, LPE18:1 also increased the invasive ability of tumor cells, although it did not alter the expression of EMT-related markers such as N-cadherin, E-cadherin, or ZEB2 (Supplementary Fig. 2e, f).

To explore whether LPE18:1 is involved in lipid accumulation in ccRCC cells, we treated cells with LPE18:1 and examined lipid deposition via BODIPY, Nile red, and Oil red O staining. Although LPE18:1 treatment increased lipid deposition in both normal HK2 cells and ccRCC cells, the promoting effect on 786-O and 769-P cells was more pronounced than that on HK2 cells (Fig. 2e–g and Supplementary Fig. 2g–i). To examine whether the proproliferative and lipogenic effects of LPE18:1 are specific to ccRCC, we extended our analysis to ACHN cells, a model of papillary RCC (pRCC). Notably, the ability of LPE18:1 to promote proliferation and lipid deposition in ACHN cells was significant but less pronounced than that in ccRCC cells (Supplementary Fig. 2j, k), suggesting subtype-specific sensitivity to LPE18:1. We subsequently measured the levels of cholesterol and triglycerides in these cells. Compared with those in normal renal epithelial HK-2 cells, LPE18:1 significantly increased the intracellular levels of cholesterol and triglycerides in 786-O and 769-P cells (Fig. 2h, i). To further determine whether LPE18:1-induced lipid accumulation affects energy metabolism, we performed Seahorse XF Cell Mito Stress Tests and ATP content assays. The results revealed that LPE18:1 treatment significantly increased the oxygen consumption rate (OCR), basal respiration, maximal respiration, and ATP production (Supplementary Fig. 2l–p), indicating that LPE18:1 induces mitochondrial oxidative phosphorylation. Collectively, these results demonstrate that LPE18:1 facilitates ccRCC cell proliferation not only by increasing lipid deposition but also by promoting lipid utilization for mitochondrial energy generation.

### LPE18:1 facilitates CAPZA1 expression in ccRCC

**Fig. 3:**
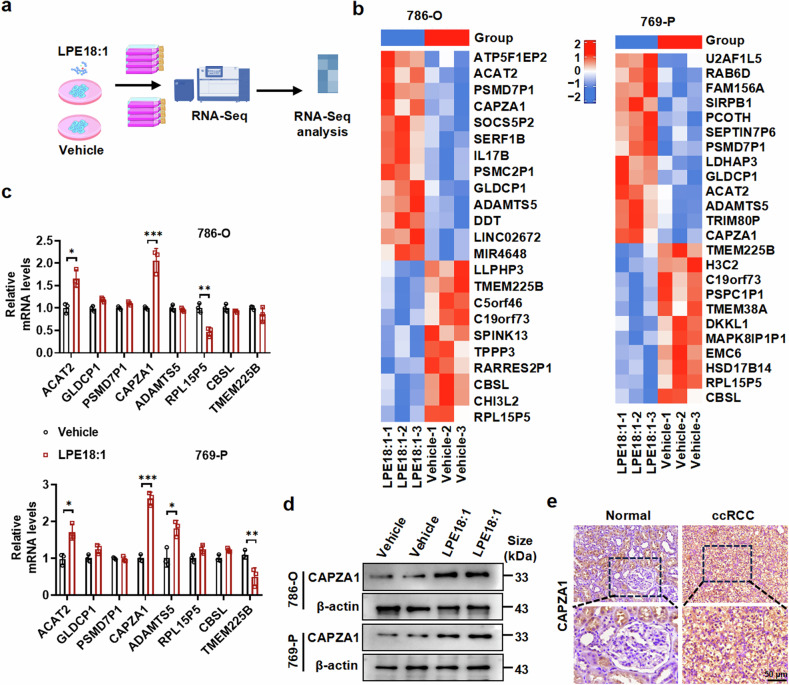
LPE18:1 induces CAPZA1 upregulation in ccRCC cells and tissues. **a** Schematic workflow of transcriptome sequencing in 786-O and 769-P cells treated with or without LPE18:1 (40 μM, 24 h). **b** Heatmap of differentially expressed genes between LPE18:1-treated and control cells. Red and blue indicate up- and downregulated genes, respectively ( | log₂FC | > 1, P < 0.05). **c** RT‒qPCR validation of candidate genes (including CAPZA1 and ACAT2) in 786-O and 769-P cells after LPE18:1 treatment. **d** Western blot analysis of CAPZA1 protein expression in LPE18:1-stimulated ccRCC cells. **e** Representative IHC images showing CAPZA1 expression in human ccRCC and adjacent normal tissues. Scale bar = 50 μm. The data are presented as the means ± SDs (n ≥ 3 per group). *P < 0.05, **P < 0.01, ***P < 0.001 versus the control

To investigate how LPE18:1 regulates cell proliferation and lipid metabolism, we treated 786-O and 769-P cells with LPE18:1 and performed transcriptomic analysis (Fig. 3a). The results indicated that genes, including ACAT2, ADAMTS5, CAPZA1, RPL15P5, and TMEM225B, were differentially expressed between LPE18:1-treated 786-O and 769-P cells and untreated cells (Fig. 3b and Supplementary Tables 4, 5). We further validated these findings via RT‒qPCR, confirming that CAPZA1 and ACAT2 expression was consistently upregulated in both cell lines following LPE18:1 treatment (Fig. 3c). Among these genes, CAPZA1 showed the most pronounced upregulation, leading us to examine its protein expression by Western blot. The results demonstrated that LPE18:1 significantly increased CAPZA1 protein levels (Fig. 3d). However, LPE18:1 treatment did not promote CAPZA1 mRNA or protein expression in HK2 cells (Supplementary Fig. 3a, b). Notably, treatment with oleic acid (OA), a common lipid species, failed to recapitulate the robust upregulation of CAPZA1 induced by LPE18:1 (Supplementary Fig. 3c), suggesting that the upregulation of CAPZA1 expression is not a general response to lipid exposure but is relatively specific to LPE18:1.

We next assessed CAPZA1 expression in clinical ccRCC tissues. IHC analysis revealed significantly higher CAPZA1 levels in ccRCC samples than in normal renal tissues (Fig. 3e and Supplementary Fig. 3d). Western blot and RT‒qPCR analyses corroborated these findings (Supplementary Fig. 3e–g). Furthermore, bioinformatics analysis of the TCGA-KIRC database indicated that CAPZA1 expression is significantly elevated in ccRCC tissues (Supplementary Fig. 3h). Clinicopathological analysis revealed that high CAPZA1 expression was positively correlated with advanced T stage, N stage, and M stage (Supplementary Fig. 3i–k). ROC curve analysis suggested that CAPZA1 can serve as a significant biomarker for ccRCC (Supplementary Fig. 3l). K‒M survival analysis revealed that high CAPZA1 expression was associated with worse overall survival in ccRCC patients (Supplementary Fig. 3m). Together, these results demonstrate that CAPZA1, a downstream effector of LPE18:1, is upregulated in ccRCC and is correlated with aggressive clinicopathological features and poor prognosis.

### CAPZA1 mediates LPE18:1-promoted ccRCC proliferation and lipid deposition

**Fig. 4:**
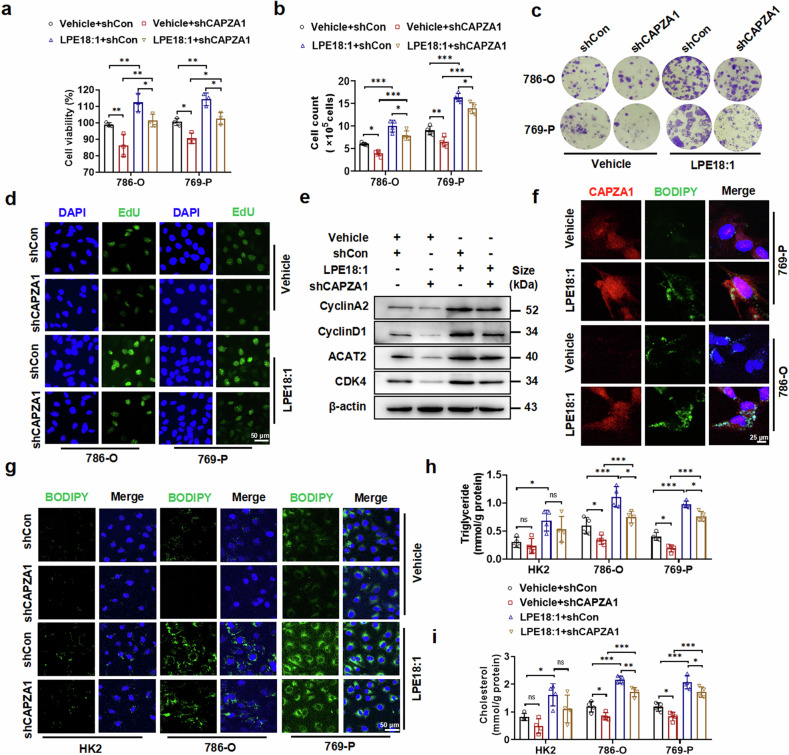
CAPZA1 is required for LPE18:1-induced proliferation and lipid deposition in ccRCC cells. **a** Viability of shControl- or shCAPZA1-transduced 786-O and 769-P cells as measured by a CCK-8 assay. Proliferation was assessed by a cell counting assay (**b**), colony formation (**c**) and EdU incorporation (**d**) in shCAPZA1 or shControl cells treated with/without LPE18:1 (40 μM, 24 h). **e** Western blot analysis of proliferation markers (Cyclin D1, Cyclin A2, and CDK4) and the lipid metabolic enzyme ACAT2 in 786-O cells under the indicated conditions. **f** Immunofluorescence showing colocalization of CAPZA1 (red) and lipid droplets (BODIPY, green) in LPE18:1-treated cells. Nuclei were stained with DAPI (blue). Scale bars = 25 μm. **g** BODIPY staining of lipid droplets in HK-2, 786-O, and 769-P cells after CAPZA1 knockdown and LPE18:1 treatment. Green: lipids; blue: nuclei. Scale bars = 50 μm. Intracellular triglyceride (**h**) and total cholesterol (**i**) levels under the corresponding treatments. The data represent the means ± SDs (n ≥ 3 per group). *P < 0.05, **P < 0.01, ***P < 0.001 vs. the control

To investigate the role of CAPZA1 in ccRCC cells, we generated 786-O and 769-P cells with stable CAPZA1 knockdown via shRNA transfection. Both the shCAPZA1-#1 construct and the shCAPZA1-#2 construct demonstrated efficient knockdown efficacy, as confirmed by significant reductions in the CAPZA1 mRNA and protein levels (Supplementary Fig. 4a, b). We next assessed the functional role of CAPZA1 in regulating cell viability and proliferative capacity. CAPZA1 knockdown significantly inhibited the increase in cell viability caused by LPE18:1 treatment (Fig. 4a, b). Colony formation assays revealed that LPE18:1 treatment promoted cell proliferation, whereas CAPZA1 knockdown attenuated this effect (Fig. 4c and Supplementary Fig. 4c). EdU incorporation assays yielded consistent results (Fig. 4d and Supplementary Fig. 4d). Western blot analysis revealed that CAPZA1 inhibition obviously suppressed the LPE18:1-induced upregulation of key proliferation regulators (CDK4 and Cyclin D1) as well as the cholesterol esterification enzyme ACAT2 (Fig. 4e and Supplementary Fig. 4e). Immunofluorescence staining and RT‒qPCR further confirmed that CAPZA1 knockdown significantly downregulated ACAT2 expression (Supplementary Fig. 4f, g).

To explore whether CAPZA1 is involved in LPE18:1-regulated lipid metabolism, we performed immunofluorescence colocalization analysis and observed that LPE18:1 treatment upregulated CAPZA1 expression and enhanced intracellular lipid accumulation (Fig. 4f). We then examined lipid deposition via BODIPY staining in HK2, 786-O, and 769-P cells following CAPZA1 knockdown and LPE18:1 treatment. Although LPE18:1 increased lipid deposition in both normal renal and ccRCC cells, the effect was more pronounced in 786-O and 769-P cells than in HK2 cells. CAPZA1 knockdown significantly attenuated LPE18:1-induced lipid accumulation in ccRCC cells but not in HK2 cells (Fig. 4g and Supplementary Fig. 4h). Nile red staining further validated these results (Supplementary Fig. 4i, j). Biochemical analyses demonstrated that CAPZA1 inhibition suppressed LPE18:1-induced accumulation of triglycerides (TGs) and total cholesterol (TC) in ccRCC cells, which was consistent with the observed reduction in lipid droplets (Fig. 4h, i).

To directly link ACAT2 to cholesterol esterification, we quantitatively assessed the conversion rate of free cholesterol to cholesteryl ester. As shown in Supplementary Fig. 4k, LPE18:1 stimulation significantly increased the ratio of cholesteryl ester to free cholesterol, indicating enhanced esterification. Conversely, ACAT2 knockdown markedly reduced LPE18:1-driven cholesterol esterification. These data provide functional evidence that ACAT2 is the key enzyme responsible for esterifying free cholesterol to form stored cholesteryl ester in response to LPE18:1. Collectively, these findings demonstrate that CAPZA1 serves as a crucial mediator in LPE18:1-induced cellular processes, orchestrating both proliferative activation and lipid accumulation in ccRCC cells.

### CAPZA1 deletion attenuates LPE18:1-induced ccRCC tumor growth and lipid deposition in vivo

**Fig. 5:**
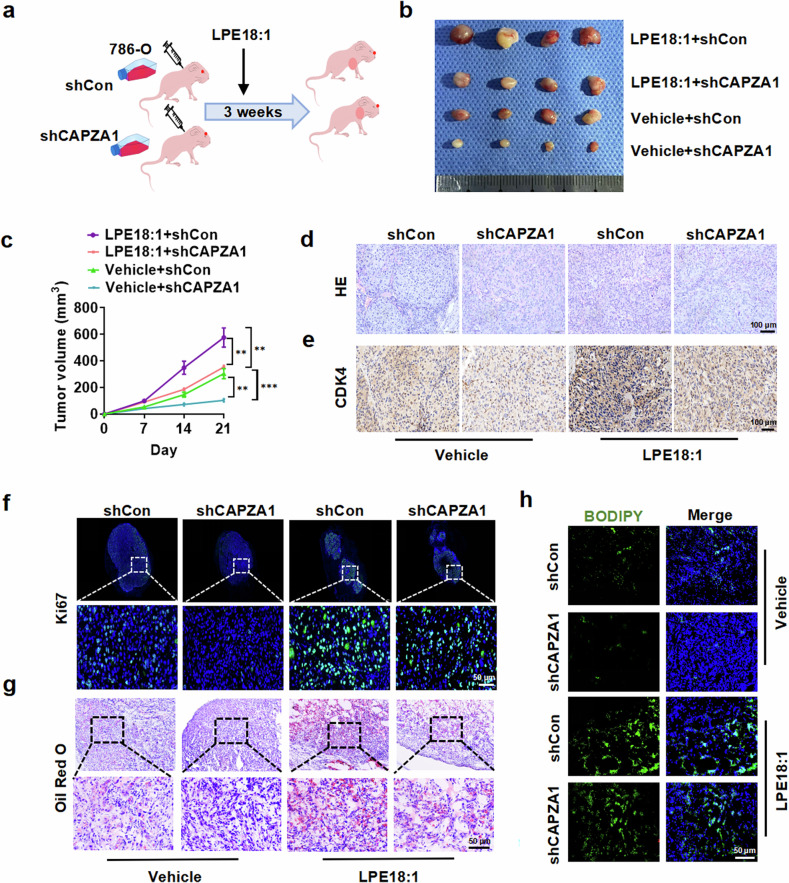
CAPZA1 deletion attenuates LPE18:1-induced tumor growth and lipid deposition in vivo. **a** Schematic of the xenograft tumor model established using 786-O cells with stable CAPZA1 knockdown in nude mice. (n = 8 per group). **b** Representative images of excised subcutaneous tumors from each treatment group. **c** Tumor growth curves monitored over time. Hematoxylin and eosin (H&E) staining (**d**) and immunohistochemical (IHC) analysis of CDK4 (**e**) in xenograft tumor sections. Scale bars = 100 μm. **f** Immunofluorescence staining of Ki-67 in tumor tissues. Scale bar = 50 μm. Lipid deposition detected by Oil Red O (**g**) and BODIPY 493/503 (**h**) staining. Red/green: lipids; blue: DAPI-stained nuclei. Scale bars = 50 μm. The data represent the means ± SDs (n ≥ 3 per group). *P < 0.05, **P < 0.01, ***P < 0.001 vs. corresponding controls

To assess the physiological significance of the LPE18:1/CAPZA1 axis in ccRCC pathogenesis, we established a xenograft tumor model through subcutaneous implantation of 786-O cells with stable CAPZA1 knockdown into nude mice (Fig. 5a). Histopathological analysis of xenograft tumors revealed that LPE18:1 treatment alone significantly promoted tumor growth, whereas simultaneous CAPZA1 knockdown obviously attenuated LPE18:1-induced tumor progression (Fig. 5b). Both the tumor volume measurements and wet weight analysis yielded consistent results (Fig. 5c and Supplementary Fig. 5a). Histomorphology analysis of xenograft tumor specimens revealed that LPE18:1 treatment significantly enhanced cell proliferation, whereas CAPZA1 knockdown effectively abrogated the LPE18:1-induced proliferative response (Fig. 5d, e and Supplementary Fig. 5b). Immunofluorescence analysis of Ki-67 expression yielded consistent results (Fig. 5f and Supplementary Fig. 5c). We subsequently assessed lipid deposition in xenograft tumor tissues via Oil Red O staining and found that LPE18:1 treatment significantly promoted intracellular lipid accumulation, whereas concurrent CAPZA1 knockdown nearly completely abrogated LPE18:1-induced lipid deposition (Fig. 5g and Supplementary Fig. 5d). Consistent with these findings, BODIPY fluorescence staining also revealed a significant reduction in LPE18:1-induced lipid deposition upon CAPZA1 deletion (Fig. 5h and Supplementary Fig. 5e). Taken together, these findings demonstrate that CAPZA1 plays a pivotal role in mediating LPE18:1-induced lipid accumulation and tumor progression in ccRCC.

### SIRT6 is a key downstream effector of CAPZA1 in ccRCC

**Fig. 6:**
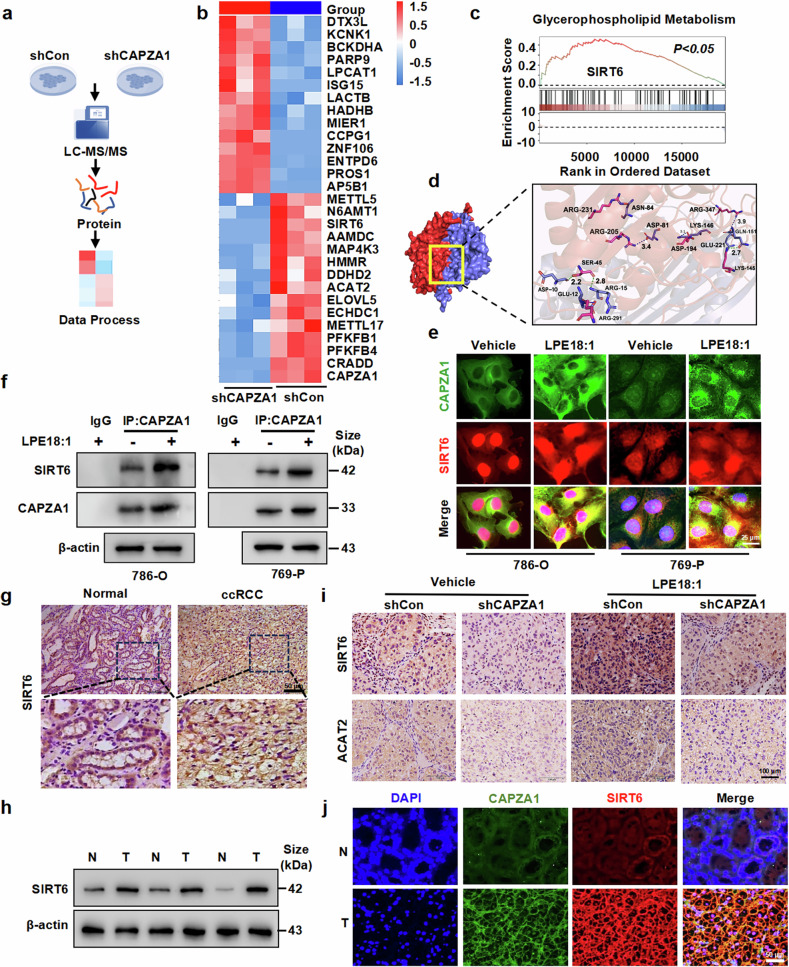
SIRT6 acts as a key downstream effector of CAPZA1 in ccRCC. **a** Schematic workflow for the identification of CAPZA1-binding proteins by immunoprecipitation‒mass spectrometry (IP‒MS) in 769-P cells. **b** Heatmap of proteins differentially associated with CAPZA1 upon shCAPZA1 knockdown (red: upregulated; blue: downregulated). **c** GSEA enrichment plot indicating the SIRT6-modulated glycerophospholipid metabolism pathway in the TCGA-KIRC cohort (*P* < 0.05). **d** Predicted molecular docking model illustrating hydrogen-bond interactions between CAPZA1 and SIRT6. **e** Confocal microscopy images showing colocalization of CAPZA1 and SIRT6 in 786-O and 769-P cells. Scale bars = 25 μm. **f** Coimmunoprecipitation (Co-IP) confirming the enhanced CAPZA1–SIRT6 interaction under LPE18:1 treatment. **g** IHC staining of SIRT6 in paired normal kidney and ccRCC clinical tissues. Scale bar = 50 μm. **h** Western blot analysis of SIRT6 protein levels in ccRCC and adjacent normal tissues. **i** IHC detection of SIRT6 and ACAT2 in xenograft tumors from different treatment groups. Scale bar = 100 μm. **j** Dual immunofluorescence staining of CAPZA1 (red) and SIRT6 (green) in human ccRCC and normal kidney tissues. Nuclei were stained with DAPI (blue). Scale bars = 50 μm. The data represent the means ± SDs (n ≥ 3 per group). *P < 0.05, **P < 0.01, ***P < 0.001

To elucidate the molecular mechanisms underlying the role of CAPZA1 in ccRCC pathogenesis, we conducted immunoprecipitation‒mass spectrometry (IP‒MS) analysis to identify potential CAPZA1-interacting proteins (Fig. 6a). IP‒MS analysis revealed 360 differentially expressed proteins between shCAPZA1- and shCon-transfected cells (Supplementary Table 6). Bioinformatics analysis and gene set enrichment analysis (GSEA) consistently revealed that SIRT6 is a promising CAPZA1-interacting candidate (Fig. 6b, c and Supplementary Fig. 6a). SIRT6, an NAD + -dependent deacetylase, regulates diverse biological processes, including DNA repair, the oxidative stress response, and lipid metabolism homeostasis, and has dual roles in cancer development and progression^30^. Molecular docking analysis revealed the presence of specific hydrogen bond interactions between CAPZA1 and key amino acid residues of SIRT6 (Fig. 6d). To investigate whether the CAPZA1-SIRT6 interaction is regulated by LPE18:1, we performed dual-label immunofluorescence colocalization analysis. The results demonstrated that LPE18:1 treatment not only upregulated SIRT6 expression but also enhanced its interaction with CAPZA1 (Fig. 6e and Supplementary Fig. 6b). Furthermore, coimmunoprecipitation (co-IP) followed by Western blot analysis provided additional evidence that LPE18:1 enhanced the physical interaction between CAPZA1 and SIRT6 (Fig. 6f). TCGA-KIRC database analysis revealed that the expression of SIRT6 was significantly greater in paired and unpaired ccRCC tissues than in normal kidney tissues (Supplementary Fig. 6c). In addition, analysis of clinicopathological features revealed that high expression of SIRT6 in ccRCC patients was positively correlated with clinical T and G stages (Supplementary Fig. 6d, e). Importantly, Kaplan‒Meier survival analysis revealed that ccRCC patients with high SIRT6 expression had significantly poorer overall survival than did patients with low SIRT6 expression (Supplementary Fig. 6f). We subsequently examined SIRT6 expression in clinical samples. Immunohistochemical analysis revealed significantly higher SIRT6 levels in ccRCC tissues than in adjacent normal tissues (Fig. 6g and Supplementary Fig. 6g). These findings were further validated by consistent results from both Western blot analysis and RT‒qPCR (Fig. 6h and Supplementary Fig. 6h, i). In addition, we evaluated SIRT6 expression in xenografts and found that LPE18:1 treatment significantly promoted SIRT6 expression, whereas the deletion of CAPZA1 blocked the promoting effects of LPE18:1 on SIRT6 expression (Fig. 6i and Supplementary Fig. 6j). Immunofluorescence double staining revealed that high expression of CAPZA1 was accompanied by increased expression of SIRT6 in ccRCC tissues (Fig. 6j and Supplementary Fig. 6k). These findings suggest that SIRT6 functions as a downstream effector of CAPZA1 and LPE18:1, potentially driving ccRCC progression.

### SIRT6 is required for LPE18:1-induced cell growth and lipid accumulation in vitro and in vivo

**Fig. 7:**
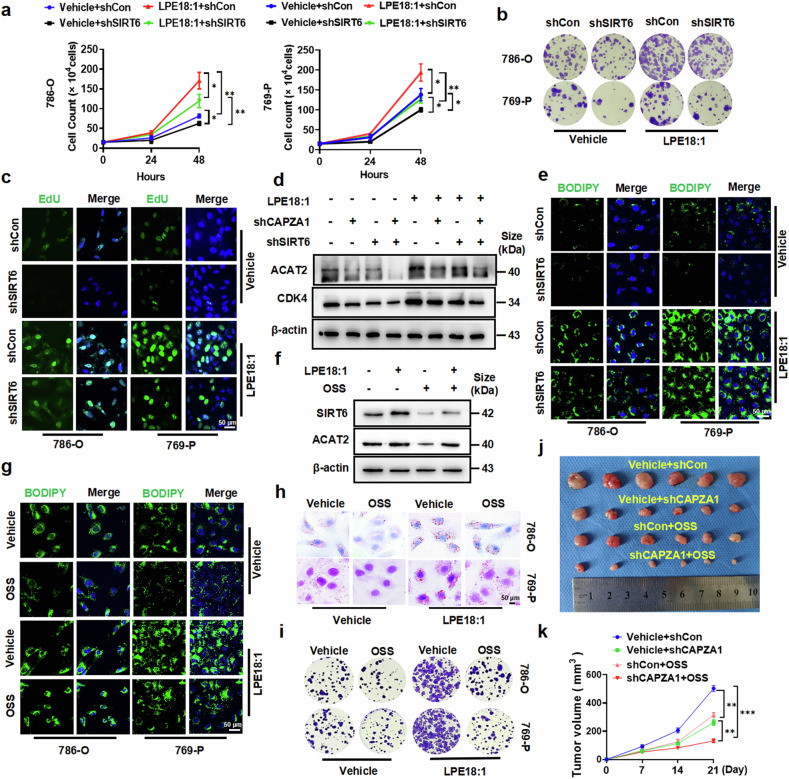
SIRT6 is essential for LPE18:1/CAPZA1-induced proliferation and lipid deposition in ccRCC cells. **a** Cell growth was measured by a cell counting assay in 786-O and 769-P cells transfected with shSIRT6 or control shRNA (shCon) and treated with or without LPE18:1 (40 μM). Colony formation assay (**b**) and EdU incorporation assay (**c**) evaluating the proliferative capacity of 786-O and 769-P cells under the same conditions as in (**a**). Scale bars = 50 μm. **d** Western blot analysis of ACAT2 and CDK4 expression in 786-O cells transfected with shCAPZA1, shSIRT6, or both, followed by treatment with or without LPE18:1. **e** Lipid accumulation detected by BODIPY staining in 786-O and 769-P cells transfected with shSIRT6 or shCon and treated with or without LPE18:1 (40 μM, 24 h). Scale bars = 50 μm. **f** Western blot analysis of SIRT6 and ACAT2 expression in 786-O cells treated with OSS-128167 (OSS, 100 μM, 24 h), LPE18:1 (40 μM, 24 h), or both. Lipid accumulation was assessed via BODIPY (**g**) and Oil Red O (**h**) staining, and cell proliferation was assessed via a colony formation assay (**i**) in 786-O and 769-P cells treated with OSS, LPE18:1, or both. Scale bars = 50 μm. **j** Xenograft tumor volumes measured on day 21 (n = 12 per group). **k** Tumor growth curves of nude mice that were subcutaneously injected with CAPZA1-depleted or control 786-O cells and treated with OSS-128167 (50 mg/kg) or vehicle every 7 days for 21 days. The data are presented as the means ± SDs (n ≥ 3 per group). *P < 0.05, **P < 0.01, ***P < 0.001 compared with the respective controls

Previous studies and the above results have consistently demonstrated that SIRT6 is involved in lipid metabolism (Fig. 6c);^31,32^ however, its role in LPE18:1-induced ccRCC proliferation remains unclear. To determine whether SIRT6 is necessary for LPE18:1-mediated effects, we transfected 786-O and 769-P cells with shSIRT6 or scramble control (shCon) and validated the knockdown efficiency (Supplementary Fig. 7a, b). We found that SIRT6 knockdown significantly attenuated LPE18:1-induced cell proliferation (Fig. 7a). Consistent with these findings, colony formation assays revealed that depleting SIRT6 substantially suppressed the ability of LPE18:1 to promote colony formation (Fig. 7b and Supplementary Fig. 7c). EdU incorporation assays further confirmed that SIRT6 deficiency inhibited LPE18:1-stimulated cell proliferation (Fig. 7c and Supplementary Fig. 7d).

We next asked whether SIRT6 participates in LPE18:1-promoted lipid metabolism. Nile red staining revealed that SIRT6 knockdown markedly reduced LPE18:1-induced lipid deposition (Supplementary Fig. 7e, f). At the molecular level, Western blot analysis indicated that LPE18:1 significantly increased ACAT2 expression, and this effect was abolished by SIRT6 or CAPZA1 knockdown (Fig. 7d and Supplementary Fig. 7g). We further evaluated lipid accumulation via BODIPY and Oil Red O staining. Both methods consistently demonstrated that the loss of SIRT6 significantly impaired LPE18:1-induced lipid droplet formation in 786-O and 769-P cells (Fig. 7e and Supplementary Fig. 7h–j).

To explore the mechanistic basis of SIRT6-mediated ACAT2 upregulation—a seemingly paradoxical effect for a transcriptional repressor—we performed chromatin immunoprecipitation (ChIP) assays. We found that SIRT6 was directly enriched within a specific region (− 788 to −1247 bp) of the ACAT2 promoter, and this binding was enhanced upon LPE18:1 stimulation (Supplementary Fig. 8a). However, the acetylation levels of H3K56, a canonical SIRT6 substrate, were not changed in this region (Supplementary Fig. 8b), suggesting that SIRT6 may not act primarily as a deacetylase in this context. Bioinformatic analysis identified two putative NRF2 binding motifs within the same promoter region (Supplementary Fig. 8c). Subsequent ChIP experiments confirmed that both SIRT6 and NRF2 co-occupied this region (Supplementary Fig. 8d). Luciferase reporter assays revealed that SIRT6 overexpression increased ACAT2 promoter activity, an effect that was abolished by NRF2 knockdown (Supplementary Fig. 8e). Consistent with these findings, SIRT6 overexpression in 786-O and 769-P cells upregulated ACAT2 expression, whereas concurrent NRF2 knockdown reversed this effect (Supplementary Fig. 8f, g). These findings indicate that SIRT6 functions as a coactivator in complex with NRF2 to promote ACAT2 transcription.

To pharmacologically inhibit SIRT6, we used a SIRT6-specific inhibitor, OSS-128167, to treat 786-O and 769-P cells. The results revealed that OSS-128167 markedly reduced SIRT6 and ACAT2 protein levels and blocked LPE18:1-induced ACAT2 upregulation (Fig. 7f and Supplementary Fig. 9a). Similarly, OSS-128167 suppressed ACAT2 expression induced by SIRT6 overexpression (Supplementary Fig. 9b, c). Lipid staining confirmed that OSS-128167 treatment significantly inhibited LPE18:1-induced or SIRT6 overexpression-promoted lipid accumulation (Fig. 7g, h and Supplementary Fig. 9d–i). Furthermore, colony formation experiments revealed that OSS-128167 reversed the stimulatory effects of LPE18:1 or CAPZA1 overexpression on colony formation (Fig. 7i and Supplementary Fig. 9j–l).

To validate the role of SIRT6 in vivo, we established xenograft tumors via CAPZA1-deficient or control cells and treated the mice with OSS-128167 or vehicle (Supplementary Fig. 9m). CAPZA1 depletion significantly inhibited tumor growth, and OSS-128167 treatment further suppressed tumor progression (Fig. 7j, k and Supplementary Fig. 9n). Histological analysis demonstrated that knockdown of CAPZA1 significantly inhibited tumor cell proliferation (Supplementary Fig. 9o), lipid deposition (Supplementary Fig. 9p, q), and ACAT2 expression (Supplementary Fig. 9r) in xenograft tumors, whereas OSS-128167 treatment further enhanced these inhibitory effects (Supplementary Fig. 9o–r). Collectively, these results demonstrate that SIRT6 is essential for LPE18:1-induced proliferation and lipid accumulation in ccRCC. Mechanistically, SIRT6 directly binds to the ACAT2 promoter and cooperates with NRF2 to activate ACAT2 transcription, revealing a noncanonical coactivator role of SIRT6 in lipid metabolic reprogramming.

### CAPZA1 enhances SIRT6 stability by inhibiting the ubiquitin‒proteasome pathway

**Fig. 8:**
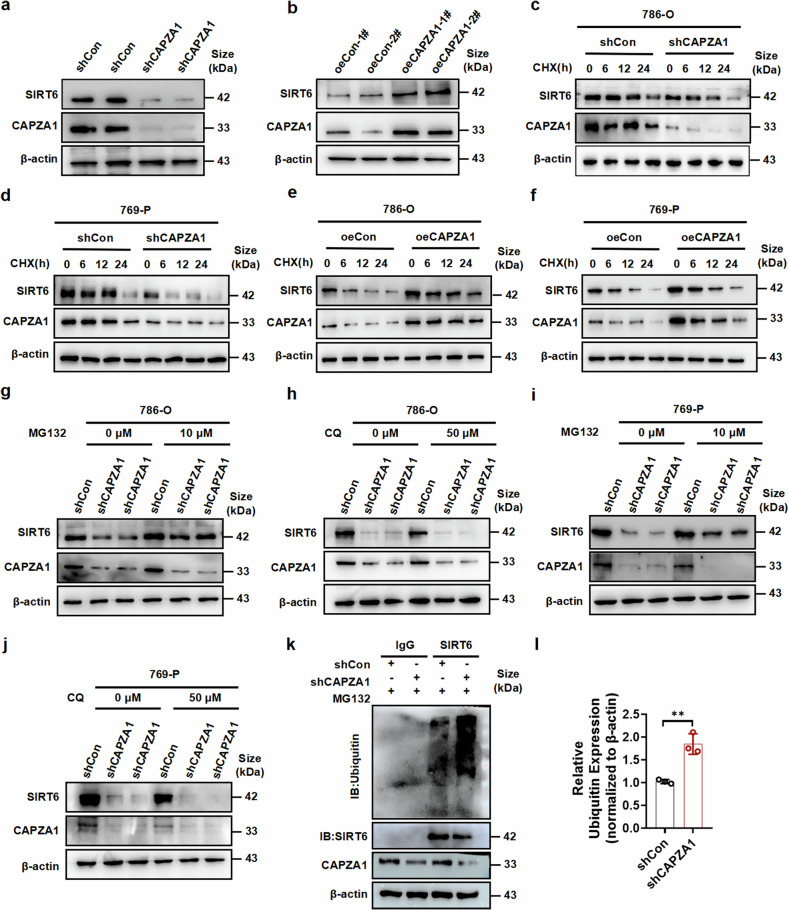
CAPZA1 enhances SIRT6 stability via the ubiquitin‒proteasome pathway. SIRT6 protein levels in 786-O cells with CAPZA1 knockdown (**a**) or overexpression (**b**). **c**, **d** SIRT6 and CAPZA1 protein levels in 786-O and 769-P cells with or without CAPZA1 knockdown following treatment with cycloheximide (CHX, 20 μg/mL) for the indicated times. **e**, **f** SIRT6 and CAPZA1 protein levels in 786-O and 769-P cells with or without CAPZA1 overexpression following CHX treatment (20 μg/mL) for the indicated times. SIRT6 and CAPZA1 protein levels in CAPZA1-knockdown cells treated with MG132 (10 μM; **g**, **h**) or chloroquine (CQ, 50 μM; **i**, **j**). **k**, **l** Ubiquitination assay: Denaturing immunoprecipitation and immunoblotting (IP/IB) of SIRT6 in CAPZA1-knockdown cells treated with MG132. The data are presented as the means ± SDs (n ≥ 3 per group). *P < 0.05, **P < 0.01, ***P < 0.001 compared with the corresponding controls

CAPZA1 has been reported to be an actin-binding protein that recruits the E3 ligase URB5 to mediate the degradation of its target proteins.^33^ The results of IP-MS combined with GO enrichment analysis revealed that ubiquitylation-related pathways were significantly enriched after CAPZA1 knockdown (Supplementary Fig. 10a). GSEA also revealed that genes highly expressed in CAPZA1-overexpressing cells were enriched in the ubiquitination pathway (Supplementary Fig. 10b). Furthermore, several studies have confirmed that SIRT6 plays a role in the regulation of ubiquitination.^34,35^ Therefore, we reasoned that CAPZA1 might affect SIRT6 expression through the regulation of ubiquitination. As expected, knockdown of CAPZA1 significantly decreased SIRT6 protein expression, whereas overexpression of CAPZA1 increased SIRT6 protein levels (Fig. 8a, b). In addition, the mRNA expression level of SIRT6 was not significantly changed by the knockdown or overexpression of CAPZA1 (Supplementary Fig. 10c, d), suggesting that SIRT6 gene expression is regulated at the posttranscriptional level. To investigate whether CAPZA1 is involved in the stability of SIRT6, 786-O and 769-P cells were transfected with shCAPZA1 and then treated with cycloheximide (CHX). The depletion of CAPZA1 significantly reduced SIRT6 protein stability (Fig. 8c, d and Supplementary Fig. 10e, f). In contrast, overexpression of CAPZA1 obviously increased SIRT6 protein stability (Fig. 8e, f and Supplementary Fig. 10g, h). These results suggest that CAPZA1 extends the half-life of the SIRT6 protein. To elucidate the molecular mechanism underlying the CAPZA1-mediated regulation of SIRT6 stability, we investigated the degradation pathways of SIRT6 in 786-O and 769-P cells via the use of pharmacological inhibitors. Treatment with the proteasome inhibitor MG132 substantially rescued the decrease in the SIRT6 protein level caused by CAPZA1 knockdown, whereas the inhibition of lysosomal activity by chloroquine (CQ) had no restorative effect (Fig. 8g–j). These results clearly indicate that SIRT6 is degraded via the ubiquitin/proteasome-dependent pathway in 786-O and 769-P cells. CoIP analysis revealed that CAPZA1 depletion significantly increased the polyubiquitination of SIRT6 (Fig. 8k, l). Together, these findings establish that CAPZA1 stabilizes SIRT6 by inhibiting its ubiquitination and thus preventing its proteasomal degradation.

### CAPZA1 promotes SIRT6 stabilization by mediating the interaction of USP48 with SIRT6

**Fig. 9:**
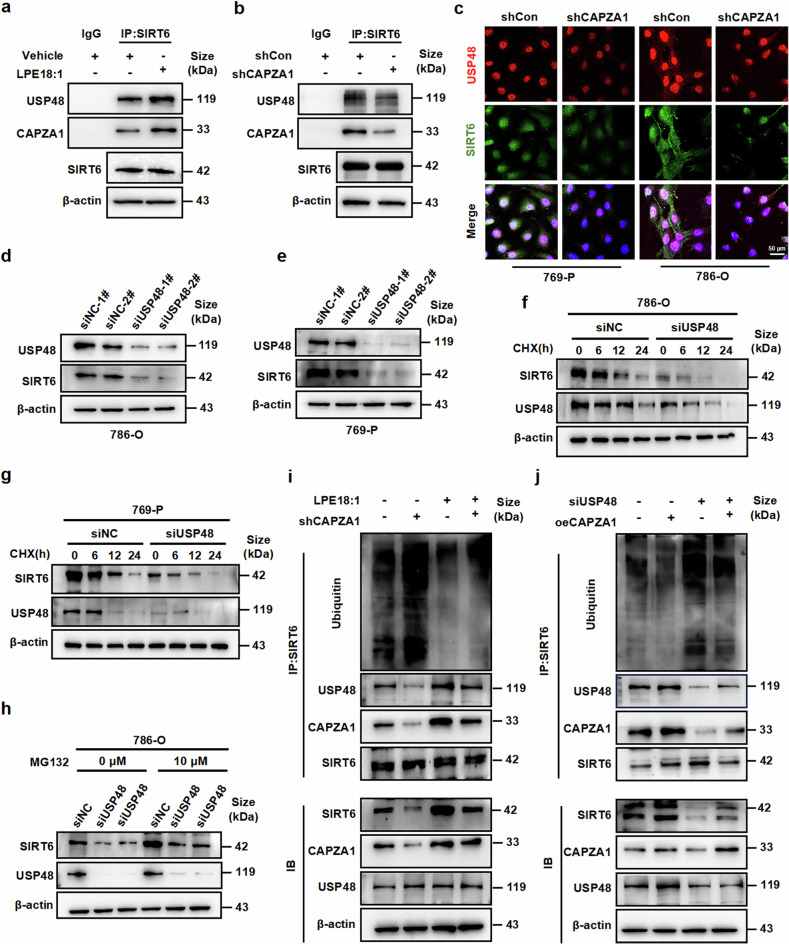
CAPZA1 recruits USP48 to deubiquitinate SIRT6. **a** Co-IP analysis of the USP48–CAPZA1 interaction in cells treated with or without LPE 18:1. **b** Co-IP validation of the USP48–SIRT6 interaction upon CAPZA1 knockdown. **c** Immunofluorescence double staining showing colocalization of USP48 and SIRT6 in 786-O and 769-P cells with or without CAPZA1 knockdown. Scale bars = 50 μm. **d, e** SIRT6 protein levels in 786-O and 769-P cells after USP48 knockdown. **f**, **g** SIRT6 degradation kinetics in USP48-knockdown 786-O and 769-P cells treated with CHX (20 μg/mL) for 0, 6, 12, and 24 h. **h** Western blot analysis of SIRT6 in 786-O cells transfected with siNC or siUSP48 and treated with MG132 (10 μM). **i** Co-IP/Western blot analysis of SIRT6 ubiquitination and CAPZA1–USP48–SIRT6 complex formation in shCAPZA1 cells treated with or without LPE18:1. **j** Co-IP analysis of SIRT6 ubiquitination and complex formation in cells transfected with siUSP48 and/or oeCAPZA1. The data are presented as the means ± SDs (n ≥ 3 per group). *P < 0.05, **P < 0.01, ***P < 0.001 compared with the corresponding controls

A previous study reported that USP48, a deubiquitinating enzyme, interacts with SIRT6.^36^ To determine whether CAPZA1 recruits USP48 to deubiquitinate SIRT6, we first analyzed the relationship between SIRT6 and USP48 from the TCGA-KIRC database, and the results revealed that USP48 expression was positively correlated with SIRT6 expression in the databases (Supplementary Fig. 11a, b). In addition, the results of bioinformatics analysis and our data revealed that USP48 was significantly upregulated in ccRCC tissues compared with normal kidney tissues (Supplementary Fig. 11c, d). CoIP analysis confirmed that SIRT6 subsequently interacted with CAPZA1 and USP48 and that LPE18:1 treatment increased their interaction (Fig. 9a). Knockdown of CAPZA1 largely reduced the interaction between USP48 and SIRT6 (Fig. 9b). The same results were obtained by immunofluorescence staining (Fig. 9c). Notably, we found that knockdown of USP48 in 786-O and 769-P cells significantly reduced the protein level of SIRT6 (Fig. 9d, e). Furthermore, we explored whether USP48 affects the stability of SIRT6. We knocked down USP48 expression with siRNA in 786-O and 769-P cells and then treated the cells with CHX, and the results revealed that the half-life of the SIRT6 protein was significantly shortened in USP48-depleted cells (Fig. 9f, g and Supplementary Fig. 11e, f). Next, we treated 786-O cells with the proteasome inhibitor MG132 and found that inhibition of the ubiquitin‒proteasome system rescued the decrease in SIRT6 protein levels caused by USP48 knockdown (Fig. 9h). Additionally, we used an anti-SIRT6 antibody to perform CoIP analysis and confirmed that LPE18:1 enhanced the interaction of SIRT6 with CAPZA1 and USP48 and decreased SIRT6 ubiquitination. However, knockdown of CAPZA1 reduced SIRT6 binding to USP48 and thus increased SIRT6 ubiquitination (Fig. 9i). Conversely, overexpression of CAPZA1 promoted the interaction of SIRT6 with USP48 and reduced the ubiquitination of SIRT6, whereas knockdown of USP48 increased the ubiquitination of SIRT6 (Fig. 9j). These findings suggest that CAPZA1 acts as a scaffold to mediate the interaction between USP48 and SIRT6, thus decreasing the ubiquitination-mediated degradation of SIRT6.

## Discussion

Clear cell renal cell carcinoma (ccRCC) is a metabolic malignancy characterized by dysregulated lipid homeostasis and aggressive progression.^37^ Despite advances in surgical management for early-stage disease,^38^ therapeutic options for metastatic ccRCC remain limited, underscoring the need to elucidate the mechanisms driving its metabolic reprogramming.^39^ Here, we revealed that peritumoral adipose tissue (PAT) adjacent to ccRCC lesions exhibited a “browning” phenotype, echoing recent findings that PAT-derived metabolites fuel tumor progression through lactate-mediated crosstalk.^10^ Expanding on these observations, our study revealed a novel lipid-mediated signaling axis driven by the glycerophospholipid derivative LPE18:1. Through untargeted metabolomics, we demonstrated that LPE18:1 drives ccRCC proliferation and lipid accumulation—a critical adaptation given the reliance of tumors on cholesterol for membrane biosynthesis.^40^ This finding aligns with emerging evidence that glycerophospholipid remodeling is a hallmark of ccRCC metabolic rewiring, although the specific mediators of this process remain poorly defined.

**Fig. 10:**
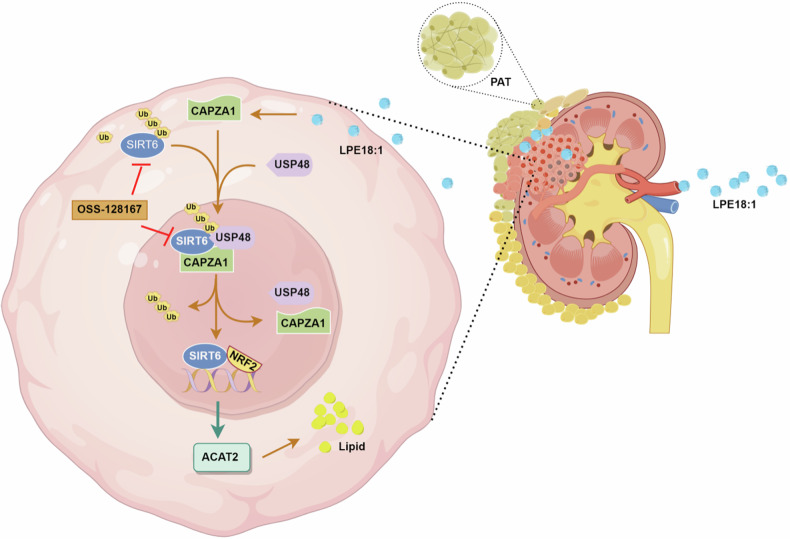
Proposed model of LPE18:1-induced lipid metabolic reprogramming via the CAPZA1/SIRT6 axis in ccRCC. Browning of perirenal adipose tissue increases LPE18:1 secretion, which is taken up by ccRCC cells. LPE18:1 upregulates CAPZA1 expression, which in turn stabilizes SIRT6 through two mechanisms: physically binding to SIRT6 to prevent its degradation and recruiting the deubiquitinase USP48 to remove ubiquitin chains from SIRT6. Stabilized SIRT6 then translocates to the nucleus, where it acts as a coactivator in complex with NRF2 to bind the ACAT2 promoter and increase its transcription. Elevated ACAT2 expression drives lipid droplet formation and cholesterol esterification, thereby promoting lipid accumulation and tumor proliferation. This mechanism highlights the role of perirenal adipose tissue in promoting ccRCC progression through lipid metabolic reprogramming. Created at https://www.figdraw.com

Central to our findings is the identification of CAPZA1 as a downstream effector of LPE18:1 signaling. While the actin-capping function of CAPZA1 is well documented,^21,41^ its role in tumor metabolism is poorly understood. We established that CAPZA1 mediates LPE18:1-driven oncogenesis through dual mechanisms: 1) stabilizing the NAD + -dependent deacetylase SIRT6 via recruitment of the deubiquitinase USP48 and 2) potentiating SIRT6-mediated upregulation of ACAT2, a key cholesterol esterification enzyme. This mechanistic cascade resolves a critical knowledge gap by linking membrane phospholipid derivatives to cholesterol deposition—a nexus of particular relevance given the characteristic lipid droplet accumulation in ccRCC.^42,43^ Notably, our discovery that the CAPZA1/USP48 interaction shields SIRT6 from proteasomal degradation adds nuance to previous reports of USP-mediated stabilization in other cancers, while the SIRT6-ACAT2 axis introduces a novel metabolic vulnerability specific to renal malignancies (Fig. 10).

Notably, LPE18:1 may exert its protumor effects through a dual mechanism: as a signaling molecule that activates the CAPZA1/SIRT6/ACAT2 axis to promote lipid storage and proliferation and potentially as a metabolic substrate that fuels mitochondrial oxidative phosphorylation. Our Seahorse data support the latter, showing enhanced OCR and ATP production upon LPE18:1 treatment (Supplementary Fig. 2l–p). This dual role aligns with the metabolic plasticity of ccRCC, which avidly scavenges lipids for both structural biosynthesis and energy generation.

The role of SIRT6 in lipid metabolism is highly context dependent and varies by cell type, metabolic status, and disease environment.^32,44,45^ While SIRT6 is widely recognized as a tumor suppressor and lipid reducer in certain contexts—such as in the liver, where it suppresses lipogenic pathways^46^ or promotes fatty acid β-oxidation,^32^ and in cardiomyocytes, where it inhibits PPARγ-mediated fatty acid uptake^47^—emerging evidence underscores its functional diversity. For example, supraphysiological overexpression of SIRT6 in hypothalamic neurons promotes diet-induced obesity,^45^ highlighting the tissue specificity of its actions. In ccRCC, a metabolic milieu defined by HIF activation and lipid accumulation, we revealed a noncanonical role of SIRT6 that diverges from its traditional function as a deacetylase and transcriptional repressor. Rather than suppressing gene expression, SIRT6 acts as a transcriptional coactivator in conjunction with NRF2 to increase ACAT2 expression, thereby facilitating cholesterol esterification and lipid droplet formation. This mechanism aligns with reports of context-specific oncogenic roles of SIRT6 in non-small cell lung cancer^48^ and skin cancer^49^ and broadens the understanding of the functional plasticity of SIRT6 in cancer biology.

Our findings that SIRT6 promotes tumorigenesis in ccRCC are consistent with recent work,^50,51^ which demonstrated that SIRT6 overexpression promoted growth in 786-O cells. Our findings elucidate the mechanisms underlying the role of SIRT6 in ccRCC by identifying an endogenous metabolite, LPE18:1, as a novel upstream activator of SIRT6 and by elucidating ACAT2 as a key downstream effector responsible for SIRT6-mediated lipid accumulation and tumor progression. This highlights the metabolic plasticity of deacetylases such as SIRT6, which can function as oncogenes or tumor suppressors depending on the tumor microenvironment.^49^ This duality is paralleled by ACAT2, in which our results revealed that ACAT2 has a protumorigenic role, which is different from previous studies that associated low ACAT2 expression with poor survival in patients with ccRCC.^52^ These discrepancies may stem from differences in tumor stage or molecular context, as cholesterol esterification by ACAT2 confers a survival advantage, especially in lipid-rich tumors. In the present study, we established that in ccRCC, SIRT6 promoted tumorigenesis by upregulating ACAT2 expression. Moreover, the weaker response to LPE18:1 in pRCC-derived ACHN cells suggests that the LPE18:1/CAPZA1/SIRT6 axis may be particularly relevant in the lipid-rich context of ccRCC. This protumorigenic activity challenges the conventional view of SIRT6 as a tumor suppressor in renal cancer and underscores the metabolic adaptability of deacetylases within distinct tumor microenvironments.^53,54^ The increased cholesterol esterification driven by this axis facilitates lipid droplet formation, which is a hallmark of ccRCC progression. Furthermore, clinical analyses showing positive correlations among CAPZA1, USP48, and SIRT6 in patient samples reinforce the translational significance of these findings. Our findings position the LPE18:1/CAPZA1/SIRT6 axis as a high-value therapeutic target for ccRCC. Pharmacological inhibition of SIRT6 via OSS-128167 significantly attenuated LPE18:1-driven lipid accumulation and tumor cell growth. Particularly compelling is the prospect of combinatorial therapies targeting both upstream lipid signaling (LPE18:1 generation) and downstream effectors (SIRT6 deacetylase activity). Furthermore, the identification of USP48 as a stabilizer of oncogenic SIRT6 expands the landscape of druggable deubiquitinating enzymes in renal cancer.

These findings advance our understanding of lipid-mediated crosstalk between metabolic reprogramming and tumor cell growth in ccRCC while raising critical questions about the influence of the microenvironment on metabolic reprogramming. Future studies should investigate whether PAT-derived LPE18:1 fuels tumor progression through endocrine signaling or local paracrine interactions. Additionally, the clinical implications of ACAT2 suppression warrant further exploration, particularly given the conflicting reports of its roles in different malignancies. As therapeutic strategies targeting lipid metabolism gain traction in oncology, our work provides a mechanistic foundation for developing precision approaches tailored to the unique metabolic vulnerabilities of ccRCC.

## Materials and methods

### Antibodies and reagents

The following antibodies were purchased for this study: anti-CAPZA1 (Proteintech, 11806-1-AP; 1:1,000 for WB), anti-SIRT6 (Proteintech, 13572-1-AP; 1:1,000 for WB), anti-ACAT2 (Abcam, ab40793; 1:1,000 for WB), anti-β-actin (Abcam, ab8226; 1:1,000 for WB), anti-ubiquitin (Proteintech, 10201-2-AP; 1:3,000 for WB), and HRP-conjugated secondary antibodies (Proteintech, SA00001-1/2; 1:10,000). Lysophosphatidylethanolamine 18:1 (LPE18:1; Avanti Polar Lipids, 846725 P) was dissolved in PBS for cell treatment. The SIRT6 inhibitor OSS-128167 (MedChemExpress, HY-128167) and the proteasome inhibitor MG132 (Selleck, S2619) were prepared in DMSO. CCK-8 assay kits (MCE, HY-K0301), EdU staining kits (Beyotime, C0071S), and Oil Red O staining kits (Solarbio, G1263) were used according to the manufacturers’ protocols.

### Human specimens

A total of 63 ccRCC tissues, paired adjacent normal kidney tissues and adipose tissues were obtained from patients undergoing nephrectomy at the Department of Urology, National Caneer Center/Cancer Hospital, Chinese Academy of Medical Sciences and Peking Union Medical College, Shengjing Hospital Affiliated with China Medical University and Shijiazhuang People’s Hospital. Perinephric adipose tissue (n = 22) and subcutaneous adipose tissue (n = 22) were collected intraoperatively. Arterial and venous blood samples from renal circulation were centrifuged at 1500 × g for 15 min to isolate the plasma. Tissues were snap-frozen in liquid nitrogen or fixed in 4% paraformaldehyde for histology.^55^ The study was conducted in accordance with the ethical standards of the respective institutional review boards and was approved by the Ethics Committee of the National Cancer Center/Cancer Hospital, Chinese Academy of Medical Sciences (No. NCC2024C-370), the Medical Ethics Committee of Shengjing Hospital Affiliated with China Medical University (No. 2024PS353K), and the Medical Research Ethics Committee of Shijiazhuang People’s Hospital (No. 2025037). All participants provided written informed consent prior to sample collection.

### Metabolomics of mass spectrometry

Metabolomic profiling of perinephric adipose tissue (PAT), ccRCC tissues, and plasma was performed via the APTBIO Metabolomics Platform (Shanghai Applied Protein Technology Co., Ltd.)^56^. Tissue samples (30 mg) were homogenized in liquid nitrogen, followed by extraction with 80% methanol/water (v/v) containing 0.1% formic acid. The plasma samples (100 μL) were deproteinized via a methanol:acetonitrile:water (2:2:1) solvent system. After centrifugation (14,000 × g, 15 min, 4 °C), the supernatants were vacuum-dried and reconstituted in 100 μL of acetonitrile/water (1:1) for LC‒MS/MS analysis. Chromatographic separation was performed on a Waters ACQUITY UPLC HSS T3 column with mobile phases A and B. Mass spectrometry was conducted on a Q Exactive HF-X system (Thermo Scientific) equipped with a heated electrospray ionization (HESI) source in both positive and negative ion modes. Full-scan MS data were acquired over 70–1050 m/z with a resolution of 60,000, and MS/MS spectra were collected in data-dependent acquisition (DDA) mode (top 10 ions, 15,000 resolution, stepped NCE 20/30/40 eV). The raw data were processed via Compound Discoverer 3.1 (Thermo Scientific) and aligned with the APTBIO in-house metabolomics database (covering HMDB, KEGG, and LipidMaps). Metabolites were annotated on the basis of accurate mass (Δ <5 ppm), retention time, and MS/MS fragmentation patterns. QC samples (pooled from all samples) were injected every 10 runs to monitor system stability. Data normalization was performed via internal standards (e.g., LPE 17:1) and total ion current (TIC), followed by multivariate analysis (PCA, PLS-DA) via SIMCA 16.0 (Umetrics) and pathway enrichment analysis via MetaboAnalyst 5.0.

### Cell lines and transfection

Human ccRCC cell lines (786-O, 769-P, A498) and normal renal tubular epithelial cells (HK-2) were cultured in RPMI-1640 (Gibco) supplemented with 10% FBS (Clark Bio) and 1% penicillin/streptomycin. For CAPZA1 or SIRT6 knockdown, cells were transfected with shRNA lentiviral vectors (GeneChem) via Lipofectamine 2000 (Invitrogen). Stable clones were selected with 2 μg/mL puromycin (Selleck). The cells were transfected with overexpression plasmids (pcDNA3.1-CAPZA1 or pcDNA3.1-SIRT6) for 48 h. The knockdown efficiency was validated via RT‒qPCR and Western blotting.^55,57^

### RNA sequencing for transcriptome profiling

PAT, ccRCC tissues and cells were extracted via TRIzol (Invitrogen). RNA integrity was assessed via a Bioanalyzer 2100 (Agilent). Libraries were prepared via the TruSeq Stranded mRNA Kit (Illumina) and sequenced on a NovaSeq 6000 (150 bp paired-end). The raw reads were aligned to the human genome (GRCh38) via STAR, and DEGs were identified with DESeq2 (|log2FC | >1, P < 0.05). Functional annotation was performed via DAVID, and protein‒protein interaction networks were visualized via STRING.^55,58^

### Data collection from the Gene Expression Omnibus (GEO) database

Publicly available RNA-seq datasets from the GEO and TCGA databases were analyzed to validate CAPZA1 and SIRT6 expression in ccRCC^58^. The raw data were processed via the GEO2R Limma tool to identify differentially expressed genes (DEGs) with |log2FC | >1 and adjusted P < 0.05. Gene set enrichment analysis (GSEA) was performed via the Molecular Signatures Database (MsigDB, accession numbers: C2curated gene sets, C5ontology gene sets) to assess pathway enrichment in TCGA. TCGA-KIRC data were accessed via cBioPortal to correlate CAPZA1/SIRT6 expression with clinical parameters.

### Animal experiments

Xenograft tumor models were established by subcutaneously injecting 5 × 10^6^ 786-O cells (suspended in 50% Matrigel) into the flanks of 6-week-old male BALB/c nude mice (SPF Biotechnology Co. Ltd., Beijing). The mice were randomly divided into four groups (n = 8 per group): control, LPE18:1-treated (20 mg/kg, intraperitoneal injection every 2 days), CAPZA1-knockdown (shCAPZA1), and combined treatment. The tumor volume was measured weekly via calipers and calculated as (length × width²)/2. After 4 weeks, the mice were euthanized, and the tumors were excised, weighed, and processed for histology or snap-frozen for biochemical analyses.^56,58^ All animal experiments were approved by the Medical Research Ethics Committee of Shijiazhuang People’s Hospital (No. 202510031).

### Cell viability assay

Cell viability was assessed via a CCK-8 kit (MCE, HY-K0301). Briefly, 786-O and 769-P cells (1.5 × 10^3/well) were seeded in 96-well plates, treated with LPE18:1 (0–80 μM) for 24–96 h, and incubated with 10 μL of CCK-8 reagent for 2 h. Absorbance at 450 nm was measured via a microplate reader (Thermo Fisher). For the colony formation assays, the cells (100/well) were cultured for 14 days, fixed with 4% paraformaldehyde, and stained with 0.1% crystal violet.^58,59^

### EdU assay

Cell proliferation was evaluated via an EdU Apollo 567 Kit (Beyotime, C0071S). The cells were incubated with 10 μM EdU for 3 h, fixed with 4% paraformaldehyde, permeabilized with 0.3% Triton X-100, and stained with click reaction buffer. Nuclei were counterstained with DAPI. Images were captured using a Leica DM6000B microscope, and the number of EdU-positive cells was quantified via ImageJ.^58^

### Western blot analysis

Total protein was extracted via RIPA lysis buffer (Beyotime) supplemented with protease inhibitors (Sigma). Proteins (20–30 μg) were separated via SDS‒PAGE, transferred to PVDF membranes (Millipore), and blocked with 5% nonfat milk. The membranes were incubated with primary antibodies (anti-CAPZA1, 1:1,000; anti-SIRT6, 1:1,000; anti-ACAT2, 1:1,000, etc.) overnight at 4 °C, followed by incubation with HRP-conjugated secondary antibodies (1:10,000). The signals were detected via enhanced chemiluminescence (ECL) (Vilber Lourmat) and analyzed with Image Lab.^58,60,61^

### RNA isolation and quantitative RT‒PCR analysis

Total RNA was extracted via TRIzol (Invitrogen), reverse-transcribed into cDNA via a Monad RT Kit, and amplified via SYBR Green Master Mix (Bio-Rad) on a CFX96 Real-Time PCR System. The primers used for gene detection were designed via Primer-BLAST (NCBI) (Supplementary Table 7). Relative mRNA levels were normalized to those of GAPDH and calculated via the 2^−ΔΔCt method.^56,62^

### Coimmunoprecipitation (CoIP) assay

CoIP was performed using the Pierce™ Classic Magnetic IP/Co-IP Kit (Thermo Scientific). The cell lysates were incubated with anti-CAPZA1 or anti-SIRT6 antibodies conjugated to protein A/G magnetic beads overnight at 4 °C. The beads were washed five times with lysis buffer, and the bound proteins were eluted with Laemmli buffer. Eluates were analyzed by Western blot or mass spectrometry.^57^

### Mass spectrometry analyses

To identify CAPZA1-interacting proteins, coimmunoprecipitated complexes were resolved by SDS‒PAGE and subjected to in-gel tryptic digestion on the APTBIO Proteomics Platform.^55^ Briefly, excised gel bands were destained, reduced with 10 mM DTT, alkylated with 55 mM iodoacetamide, and digested with sequencing-grade trypsin (Promega, V5111) at 37 °C overnight. Peptides were desalted via C18 StageTips and analyzed via nanoLC‒MS/MS on a Q Exactive HF‒X mass spectrometer (Thermo Scientific) coupled to an UltiMate 3000 HPLC system. Chromatographic separation was performed on a C18 column (75 μm × 25 cm, 1.9 μm particles) with a 120-min gradient (2–35% acetonitrile in 0.1% formic acid). MS data were acquired in data-dependent acquisition (DDA) mode: full MS scans (350–1800 m/z, 60,000 resolution) followed by MS/MS of the top 20 ions (15,000 resolution, 1.6 m/z isolation window, 28% NCE). The raw files were processed via MaxQuant (v2.2.0.0) against the UniProt Human database (2023_01 release), with carbamidomethylation as a fixed modification and methionine oxidation as a variable modification. The identifications were filtered at 1% FDR at both the peptide and protein levels. High-confidence interactors were defined as proteins with ≥2 unique peptides, detected in ≥2/3 biological replicates, and absent in the IgG control groups.

### TG and cholesterol measurement assay

The levels of intracellular triglycerides (TGs) and total cholesterol (TC) were measured via commercial kits (Applygen, E1013; Jiancheng Bioengineering, A111-1-1). The cells or tissues were homogenized in lysis buffer, and the supernatants were mixed with assay reagents. The absorbance at 510 nm (TG) or 550 nm (TC) was measured via a microplate reader.^56^

### Hematoxylin and eosin, immunofluorescence and immunohistochemical staining

Tissues were fixed in 4% paraformaldehyde, paraffin-embedded, and sectioned (4 μm). For H&E staining, the sections were dewaxed, stained with hematoxylin and eosin, and imaged. For IHC, the sections were incubated with anti-CAPZA1 (1:200) or anti-SIRT6 (1:200) antibodies overnight, followed by incubation with HRP-polymer secondary antibodies and DAB substrate. For immunofluorescence, the cells were fixed, permeabilized, and stained with Alexa Fluor 488/594-conjugated antibodies. Images were captured via a Leica confocal microscope.^57^

### Oil red O staining

The cells or frozen tissue sections were fixed with 4% paraformaldehyde, incubated with Oil Red O working solution (Solarbio, G1263) for 15 min, and counterstained with hematoxylin. Lipid droplets were visualized under a light microscope (Leica DM6000B), and the optical density was quantified at 510 nm.^56^

### BODIPY493/503 staining and Nile red staining

The cells were fixed and stained with BODIPY493/503 (5 μM, MCE) or Nile Red (1:200, Solarbio) for 30 min.^63,64^ Nuclei were counterstained with DAPI. The fluorescence intensity was analyzed via ImageJ, and images were acquired with a Leica TCS SP8 confocal microscope.

### Statistical analysis

The data are expressed as the means ± SDs from three independent experiments. Differences between groups were analyzed by Student’s t test (two groups) or one-way ANOVA (≥3 groups) via GraphPad Prism 9.0. Survival analysis was performed via the Kaplan‒Meier method with log-rank tests. A P value < 0.05 was considered to indicate statistical significance.^56^

## Supplementary information

Supplementary Materials

Original data of Western blot

## Data Availability

The data in the main manuscript or Supplementary Materials have been uploaded to the OMlX, China National Center for Bioinformation/Beijing Institute of Genomics, Chinese Academy of Sciences (https://ngdc.cncb.ac.cn/omix accession no. OMIX012596, no. OMIX012656, and no. OMIX012675).^65,66^ The data could be accessible upon the corresponding authors approval for reasonable requests without prejudice to approved ethics and local legislation via NGDC.
