# Supplementary material for: Lysophosphatidylethanolamine 18:1 drives clear cell renal cell carcinoma by stabilizing SIRT6 to reprogram lipid metabolism
Source: Signal Transduct Target Ther. 2025 Dec 8;10:398. doi: 10.1038/s41392-025-02496-1 (PMC12682907; doi:10.1038/s41392-025-02496-1)
Supplement: Supplementary file 1 — Supplementary Materials [file 41392_2025_2496_MOESM1_ESM.docx]

**Supplementary Materials for**

**Lysophosphatidylethanolamine 18:1 drives clear cell renal cell carcinoma by stabilizing SIRT6 to reprogram lipid metabolism**

Nanxi Yue **^1#^** , Hongye Zhao **^1#^**, Yong Zhang **^2#^** Junfei Gu **^3#^** Jinchun Qi **^3^**, Jinkun Wen **^1^**, Wei Wang **^1^**, Mingming Lv **^1^**, Hao Sun **^1^**, Jinsuo Chen **^4^**, Chenxiao Yang **^5^**, Changbao Qu **^6^***，Xiaonan Chen **^7^*** and Zhan Yang **^1, 5^***

Correspondence to: yangzhan@hebmu.edu.cn

**This file includes:**

Methods

Figures S1 to S11

Tables S1 to S7

**Other Supplementary Materials for this manuscript include the following:**

Original Blots

**Methods**

**ChIP analysis**

Cultured 786-O cells were fixed in formaldehyde for ChIP experiments, as previously described^1^. The cells were subjected to ultrasound treatment, which fragmented the cross-linked chromatin to an average length of 350–550 nt. The DNA fragments were then diluted 10-fold with enzyme-free water. Immunoprecipitation of the DNA fragments was performed at 4°C using anti-SIRT6，anti-H3K56ac, anti-NRF2, or anti-IgG antibodies. Following this, the samples were incubated overnight with magnetic beads treated with protein A-agarose/salmon sperm DNA. After reversing the cross-linking, the binding of proteins to the ACAT2 promoter was assessed by RT–qPCR. The sequence of the ChIP primers are provided in Supplementary Table 7.

**Luciferase assay**

The 2000 nt ACAT2 promoter sequence was inserted into the pGL3 fluorescence reporter vector, and the sequence was confirmed through Sanger sequencing, as previously described^2^. 786-O cells were plated in a 24-well plate and co-transfected with the indicated vector. Luciferase activity was assessed using a dual luciferase assay system (Promega) with Flash and Glow readers (LB955, Germany). The relative activity ratio of firefly luciferase to Renilla luciferase was used to indicate promoter activity.

**3D invasion assay**

Cells were brought to a concentration of 1×10⁶ cells/mL and blended with an equal volume of ice-cold Matrigel™ Matrix (Corning，356234), resulting in a final density of 2×10⁵ cells/mL embedded in the matrix^3^.A volume of 10 µL of this mixture was seeded at the center of each well in a 12-well plate. Polymerization was carried out by incubating the plate at 37 °C for 30 minutes. After the gel solidified, 1 mL of complete medium was gently overlaid onto each well. Cultures were maintained for 10-14 days under standard conditions (37 °C, 5% CO₂), with medium changes performed every 48 hours. On day 14, bright-field images of the gel droplets were captured at 20× magnification using an inverted microscope.

**Oxygen consumption rate assay**

The oxygen consumption rate was measured using Seahorse XFe96 analyzer (Seahorse XF Cell Mito Stress Test Kit, Agilent, 103015-100)^4^. The probe plate was hydrated with 200 μL of sterile water per well overnight at 37°C. Then, 200 μL of XF calibrant was added to each well, and the plate was incubated for an additional 45 minutes under CO2-free conditions at 37°C. The assay medium was supplemented with 10 mM glucose, 1 mM pyruvate, and 2 mM glutamine. Oligomycin, FCCP, and rotenone/antimycin A (Rot/AA) were applied at final concentrations of 1.5 μM, 1.5 μM, and 0.5 μM, respectively. After aspirating the culture medium to 20 μL, the cells were washed twice with 200 μL of the assay medium. Then, 160 μL of medium was added, and the cells were incubated for 60 minutes at 37 °C without CO₂ and analyzed with GraphPad Prism 9.0.

**Quantification of LPE18:1 by Liquid Chromatography-Mass Spectrometry (LC-MS)**

A volume of 100 μLof sample was mixed with 300 μL of methanol, followed by centrifugation at 17,000 × g for 15 min. Both the sample and mixed standard solutions were diluted 1:1 (v/v) with a 75% methanol-water solution containing internal standard (IS), yielding a final IS concentration of 250 ng/mL. Chromatographic separation was performed at a column temperature of 40 °C with an injection volume of 5 μL. The mobile phase consisted of 0.1% formic acid–10 mM ammonium formate in water and 90% acetonitrile–10 mM ammonium formate in water, under gradient elution conditions. Mass spectrometric detection was carried out using an AB Sciex 4500 system operated in negative ion mode with multiple reaction monitoring (MRM). The ion source parameters were set as follows: curtain gas, 25 psi; collision gas, 10 psi; ion spray voltage, 4500 V; and turbo gas temperature, 500 °C. Quantification was performed using MultiQuant 3.0.3 software based on an internal standard calibration curve^5^.

**
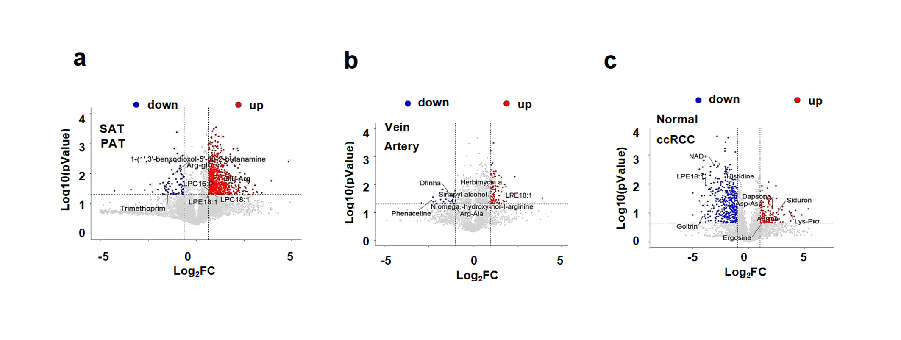
**

**Supplementary Fig. 1** Volcano plots of metabolite differential expression across PAT vs. SAT (**a**), tumor vs. normal tissue (**b**), and venous vs. arterial blood (**c**). Red/blue dots represent significantly upregulated/downregulated metabolites (|log2FC| >1, P<0.05); black dots indicate nonsignificant changes.

**
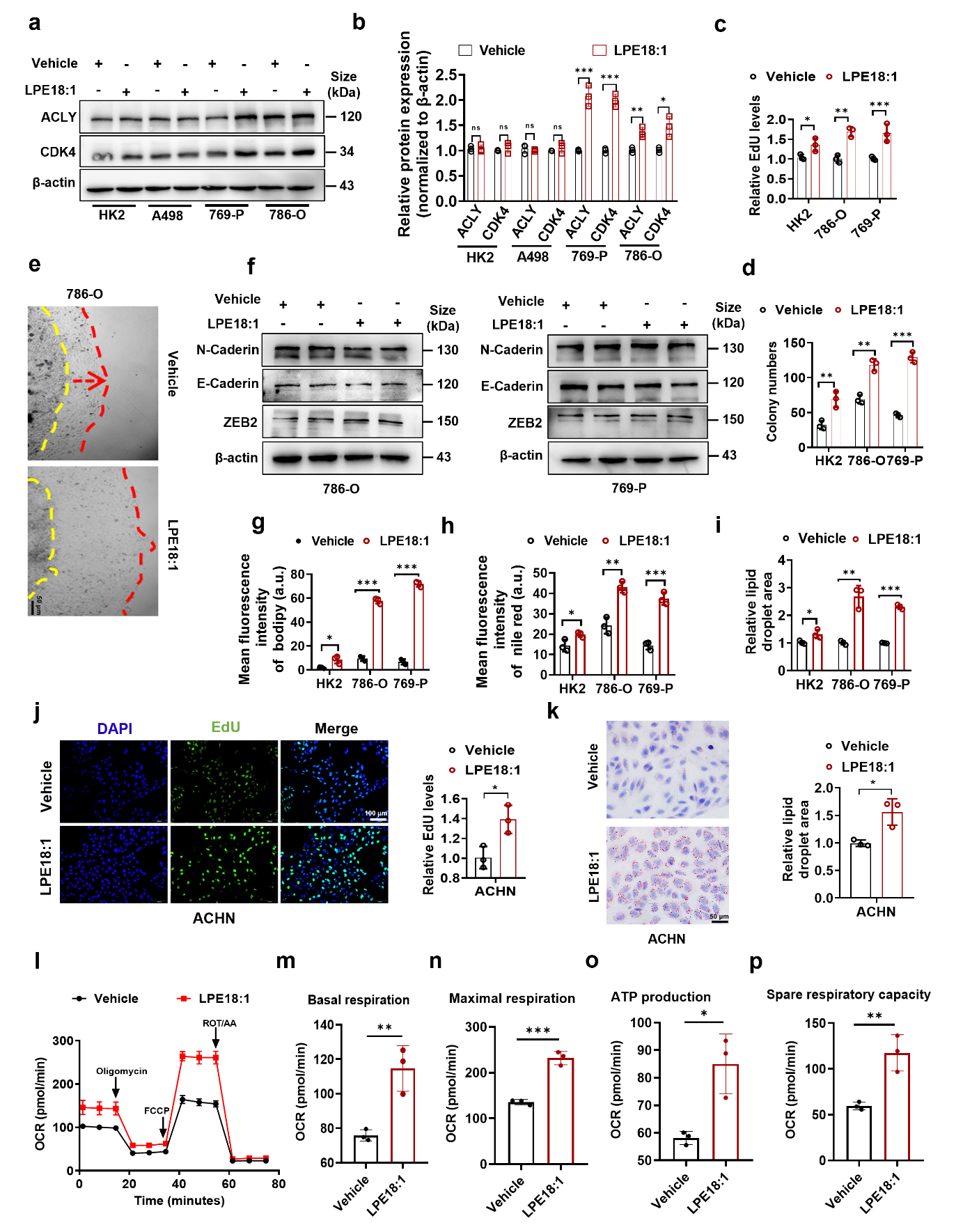
**

**Supplementary Fig. 2.** Supplementary data characterizing LPE18:1-induced effects in renal cancer models. **a, b** Western blot analysis of ACLY and CDK4 protein levels in multiple renal cell lines treated with LPE18:1 (40 μM, 24 h). **c, d** Quantification of EdU-positive cells (c) and colony numbers (d) from proliferation assays. **e** Invasion capacity of 786-O cells evaluated by 3D Matrigel assay with or without LPE18:1. **f** Protein levels of EMT markers (N-Cadherin, E Cadherin, ZEB2) in LPE18:1-treated cells. **g–i** Quantitative analysis of fluorescence intensity from BODIPY (g), Nile Red (h), and Oil Red O (i) staining. **j, k** EdU incorporation (j) Scale bars = 100 μm and Oil Red O staining (k) Scale bars = 50 μm in ACHN (pRCC) cells treated with LPE18:1. **l–p** Seahorse XF Mito Stress Test profiles in 786-O cells: OCR trace (l), basal respiration (m), maximal respiration (n), ATP production (o), and spare respiratory capacity (p). All data are presented as mean ± SD (n ≥ 3 per group). *P < 0.05, **P < 0.01, ***P < 0.001 vs. control.

**
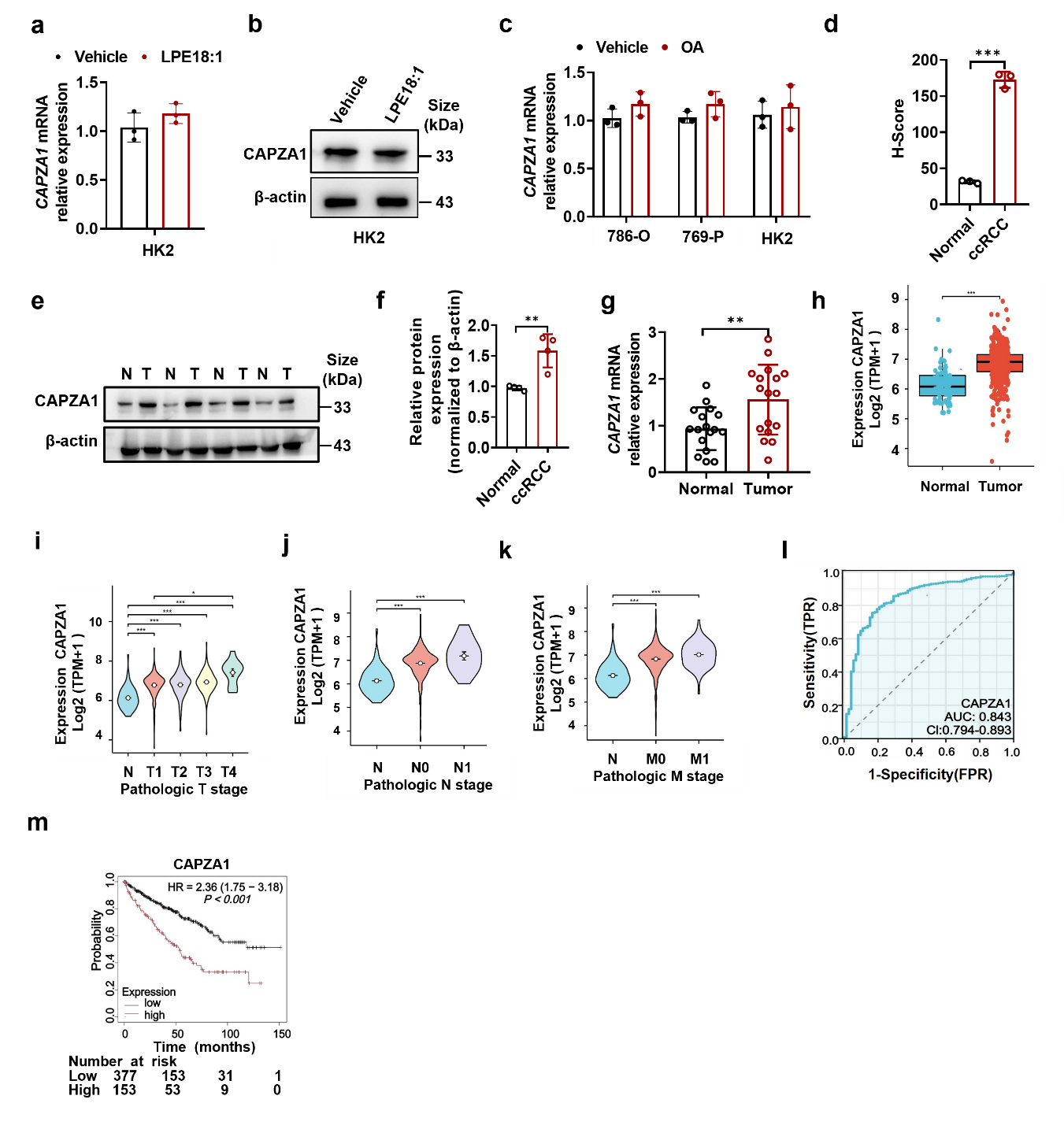
**

**Supplementary Fig. 3**. Supplementary validation and clinical relevance of CAPZA1 in ccRCC. **a, b** CAPZA1 mRNA (a) and protein (b) expression in HK-2 cells treated with or without LPE18:1 (40 μM, 24 h). **c** CAPZA1 mRNA levels in HK-2, 786-O, and 769-P cells treated with oleic acid (OA, 100 μM, 24 h). **d** Quantification of CAPZA1 IHC staining using H-score in normal kidney and ccRCC tissues. **e–g** CAPZA1 protein (e, f) and mRNA (g) expression in paired normal and tumor tissues from ccRCC patients. **h** Analysis of CAPZA1 expression in the TCGA-KIRC cohort. **I-k** Correlation between CAPZA1 expression and pathological T stage (i), N stage (j), and M stage (k) in TCGA-KIRC patients. **l** ROC curve evaluating the diagnostic value of CAPZA1 in ccRCC (AUC = 0.843). **m** Kaplan–Meier survival analysis of overall survival in ccRCC patients stratified by CAPZA1 expression. Data represent mean ± SD (n ≥ 3 per group). *P < 0.05, **P < 0.01, ***P < 0.001.

**
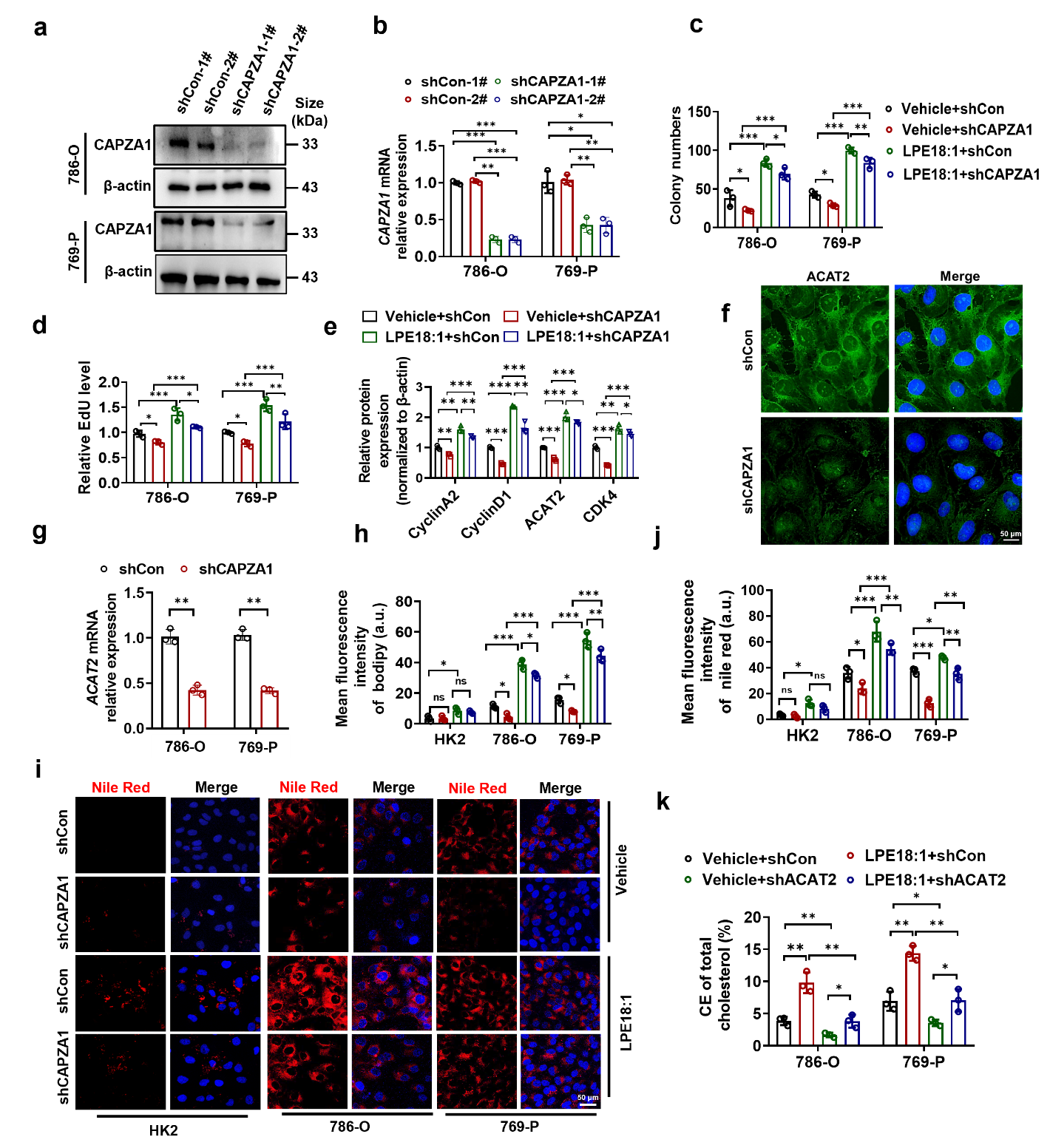
**

**Supplementary Fig. 4**. Validation of CAPZA1 knockdown and its functional consequences in lipid metabolism. **a, b** Knockdown efficiency of CAPZA1 in 786-O and 769-P cells transduced with shCAPZA1-#1/-#2 or shControl, confirmed by Western blot (a) and RT-qPCR (b). **c, d** Quantification of colony numbers (c) and EdU-positive cells (d) from proliferation assays. **e** Densitometric analysis of protein expression from Western blot in Fig. 4. **f** Immunofluorescence detection of ACAT2 expression in shCAPZA1-transfected 786-O cells. Scale bars = 50 μm **g** ACAT2 mRNA levels in 786-O and 769-P cells after CAPZA1 knockdown. **h** Quantification of BODIPY fluorescence intensity. **i, j** Nile Red staining of lipid content under CAPZA1 knockdown with/without LPE18:1. Red: neutral lipids; blue: nuclei. Scale bars = 50 μm **k** Cholesterol esterification rate measured as cholesteryl ester-to-free cholesterol ratio in 786-O and 769 P cells. All data are presented as mean ± SD (n ≥ 3 per group). *P < 0.05, **P < 0.01, ***P < 0.001.

**
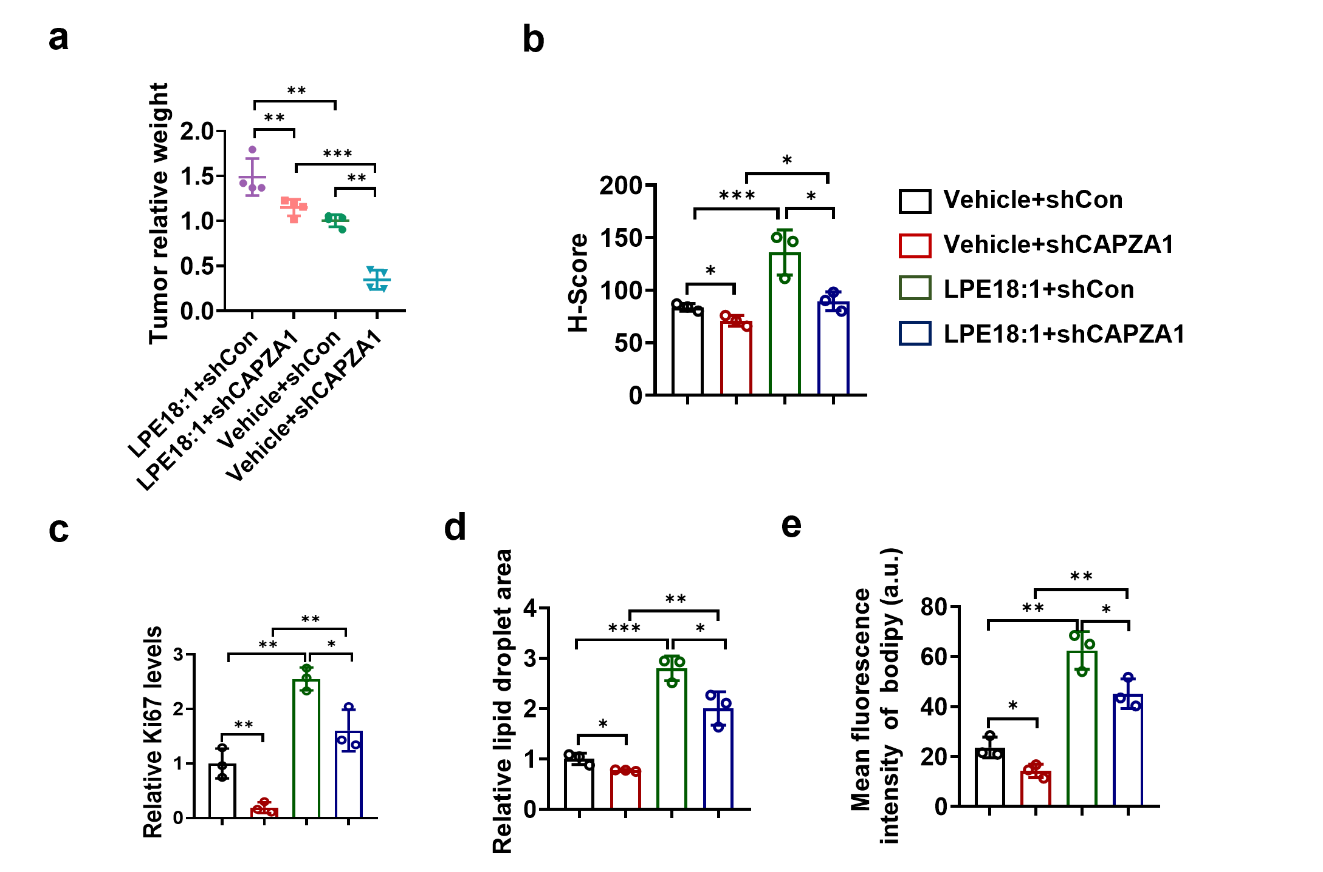
**

**Supplementary Fig. 5**. Quantitative analysis of tumor growth and lipid accumulation in xenograft models. **a** Final tumor weights across treatment groups at endpoint (21 days). **b** Quantification of CDK4 IHC staining intensity in xenograft tissues. **c** Quantitative analysis of Ki-67-positive cells from immunofluorescence. **d, e** Quantification of lipid deposition based on Oil Red O (d) and BODIPY (e) staining intensity. All data are presented as mean ± SD(n ≥ 3 per group). *P < 0.05, **P < 0.01, ***P < 0.001.


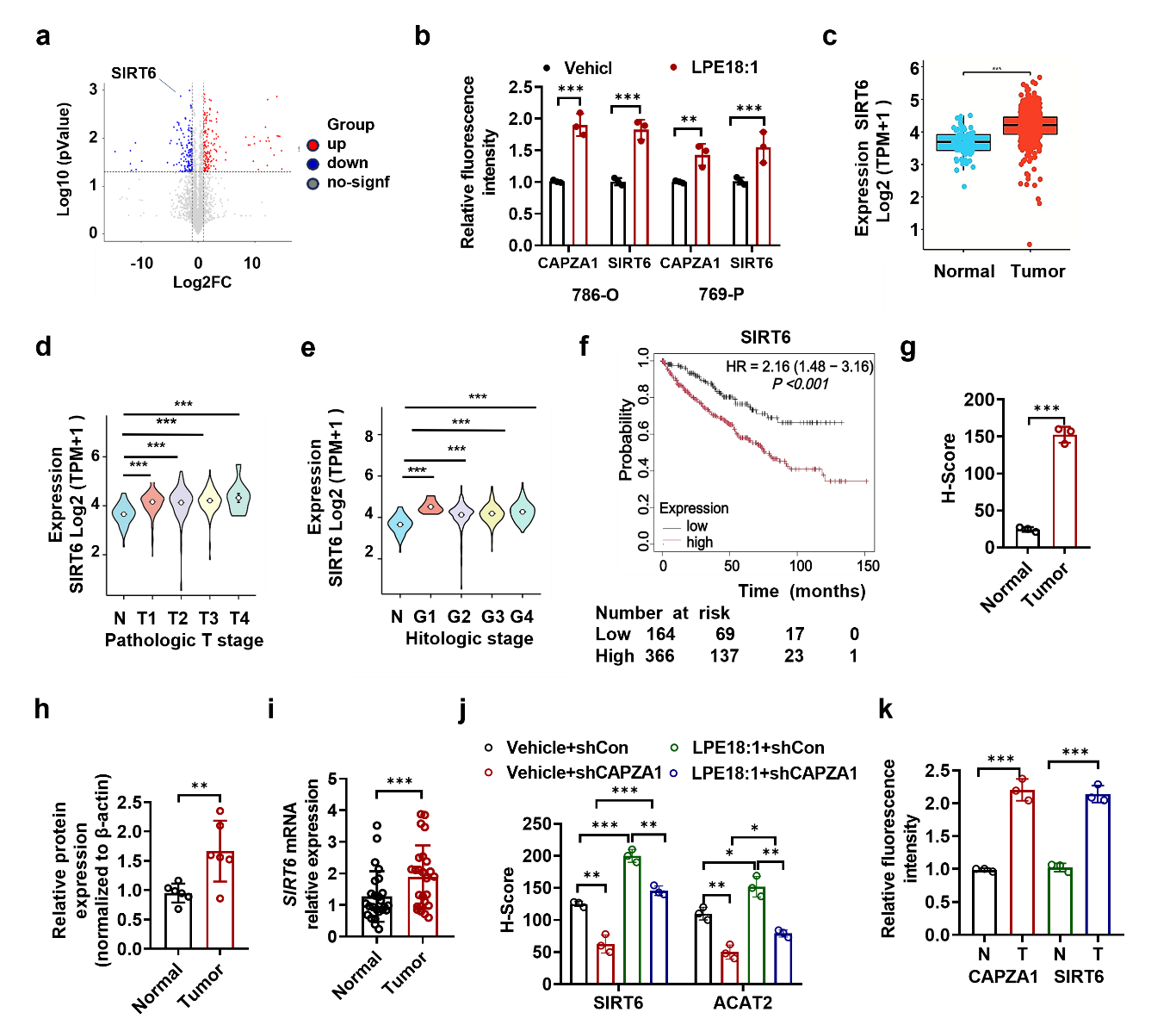


**Supplementary Fig. 6.** Clinical relevance and functional validation of SIRT6 in ccRCC. **a** Volcano plot of CAPZA1-interacting proteins identified by IP-MS in 769-P cells. **b** Quantification of CAPZA1 and SIRT6 fluorescence intensity from confocal imaging. **c** SIRT6 mRNA expression in tumor vs. normal tissues from TCGA-KIRC dataset. **d, e** Correlation between SIRT6 expression and clinical T stage (d) and histologic grade (e) in TCGA-KIRC. **f** Kaplan–Meier survival analysis of ccRCC patients stratified by SIRT6 expression level. **g** H-score quantification of SIRT6 IHC staining in normal and ccRCC tissues. **h** Densitometric analysis of SIRT6 Western blot signals in clinical samples. **i** SIRT6 mRNA expression levels measured by RT-qPCR in patient tissues. **j** H-score evaluation of SIRT6 and ACAT2 IHC staining in xenograft tumor sections. **k** Quantification of CAPZA1 and SIRT6 fluorescence intensity from confocal imaging in human ccRCC and normal kidney tissues. All data are presented as mean ± SD (n ≥ 3 per group). *P < 0.05, **P < 0.01, ***P < 0.001.


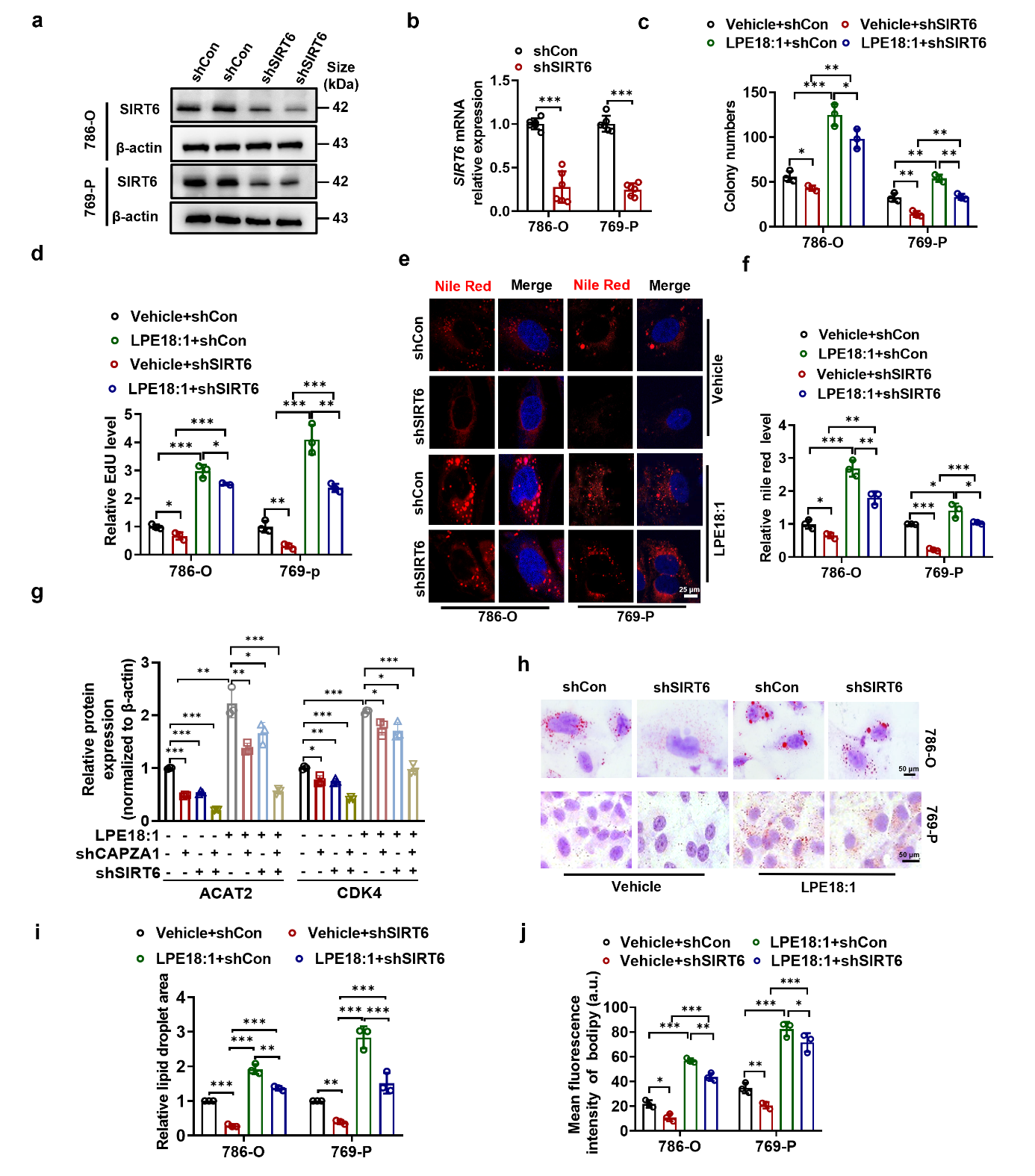


**Supplementary Fig. 7.** SIRT6 knockdown attenuates LPE18:1-induced lipid accumulation and proliferation in ccRCC cells. **a, b** Western blot (a) and RT-qPCR (b) analysis of SIRT6 expression in 786-O and 769-P cells transfected with shSIRT6 or shCon. **c, d** Quantification of colony formation (c) and EdU incorporation (d) assays. **e, f** Lipid accumulation detected by Nile Red staining in 786-O and 769-P cells transfected with shSIRT6 or shCon and treated with or without LPE18:1 (40 μM, 24 h) Scale bars = 25 μm. **g** Densitometric analysis of ACAT2 and CDK4 protein levels from Western blot in Fig. 7d. **h, i** Lipid accumulation detected by Oil Red O staining in 786-O and 769-P cells transfected and treated as in (e, f) Scale bars = 50 μm. **j** Quantification of BODIPY fluorescence intensity. Data represent mean ± SD (n ≥ 3 per group). *P < 0.05, **P < 0.01, ***P < 0.001 compared to respective controls.


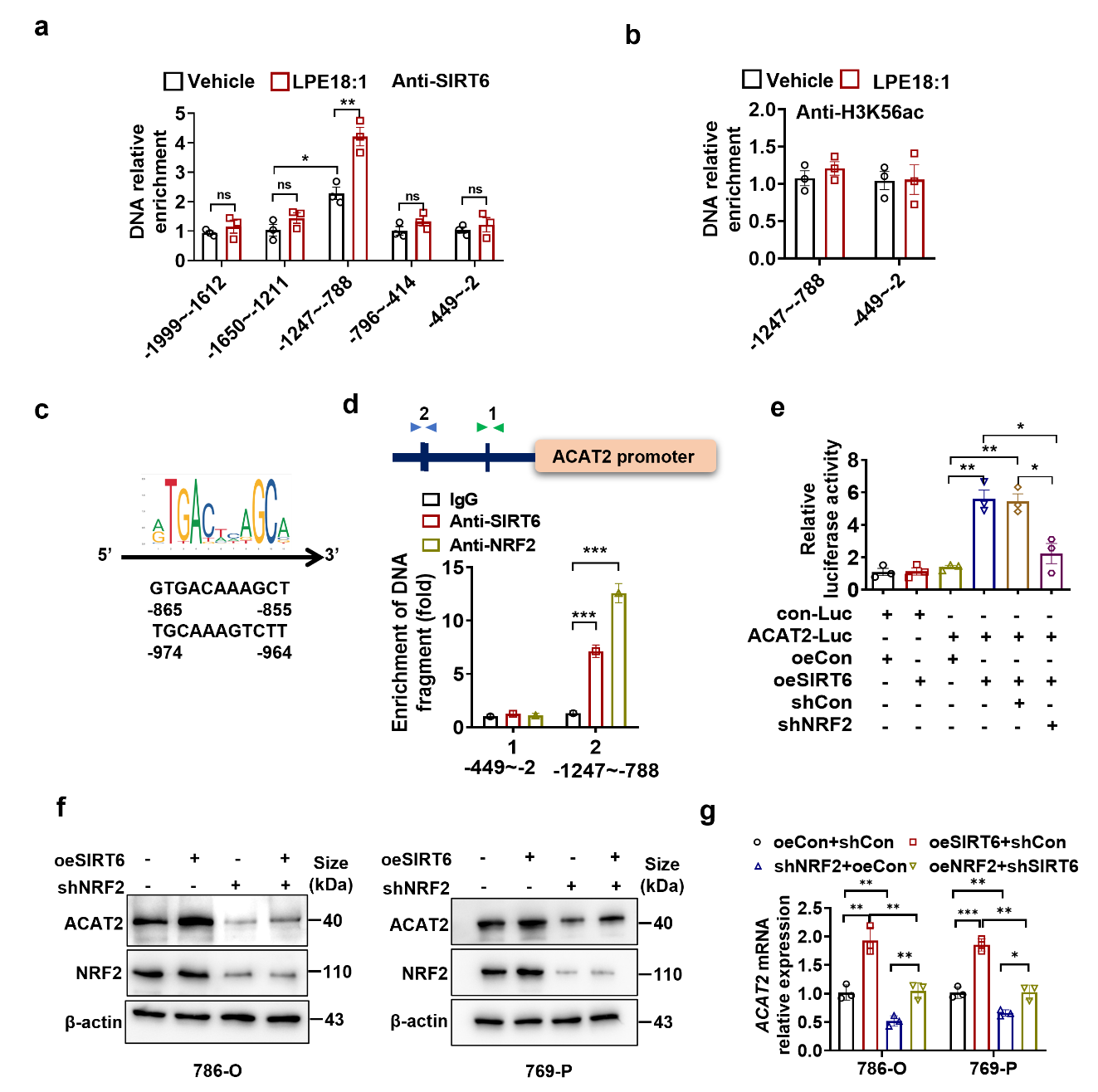


**Supplementary Fig. 8.** SIRT6 interacts with NRF2 to transcriptionally activate ACAT2 expression. **a** ChIP-PCR analysis of SIRT6 binding to the ACAT2 promoter region (up to –2000 bp) in 786-O cells treated with or without LPE18:1 (40 μM, 24 h), using an anti-SIRT6 antibody. **b** ChIP-PCR analysis of H3K56ac levels in two ACAT2 promoter regions (–2 to –449 bp and –788 to –1247 bp) in 786-O cells treated with or without LPE18:1 (40 μM, 24 h), using an acetyl-H3K56 antibody. **c** Two putative NRF2 binding motifs identified within the –2000 bp region of the ACAT2 promoter, predicted using the JASPAR database. **d** ChIP assay showing co-occupancy of SIRT6 and NRF2 on the ACAT2 promoter using anti-SIRT6 and anti-NRF2 antibodies. **e** Luciferase reporter assay evaluating the effects of SIRT6 and NRF2 on ACAT2 promoter activity in transfected cells. **f, g** Western blot (f) and RT-qPCR (g) analysis of ACAT2 expression in 786-O and 769-P cells transfected with oeSIRT6 and/or shNRF2. Data are presented as mean ± SD (n ≥ 3 per group). *P < 0.05, **P < 0.01, ***P < 0.001 compared to corresponding controls.

**
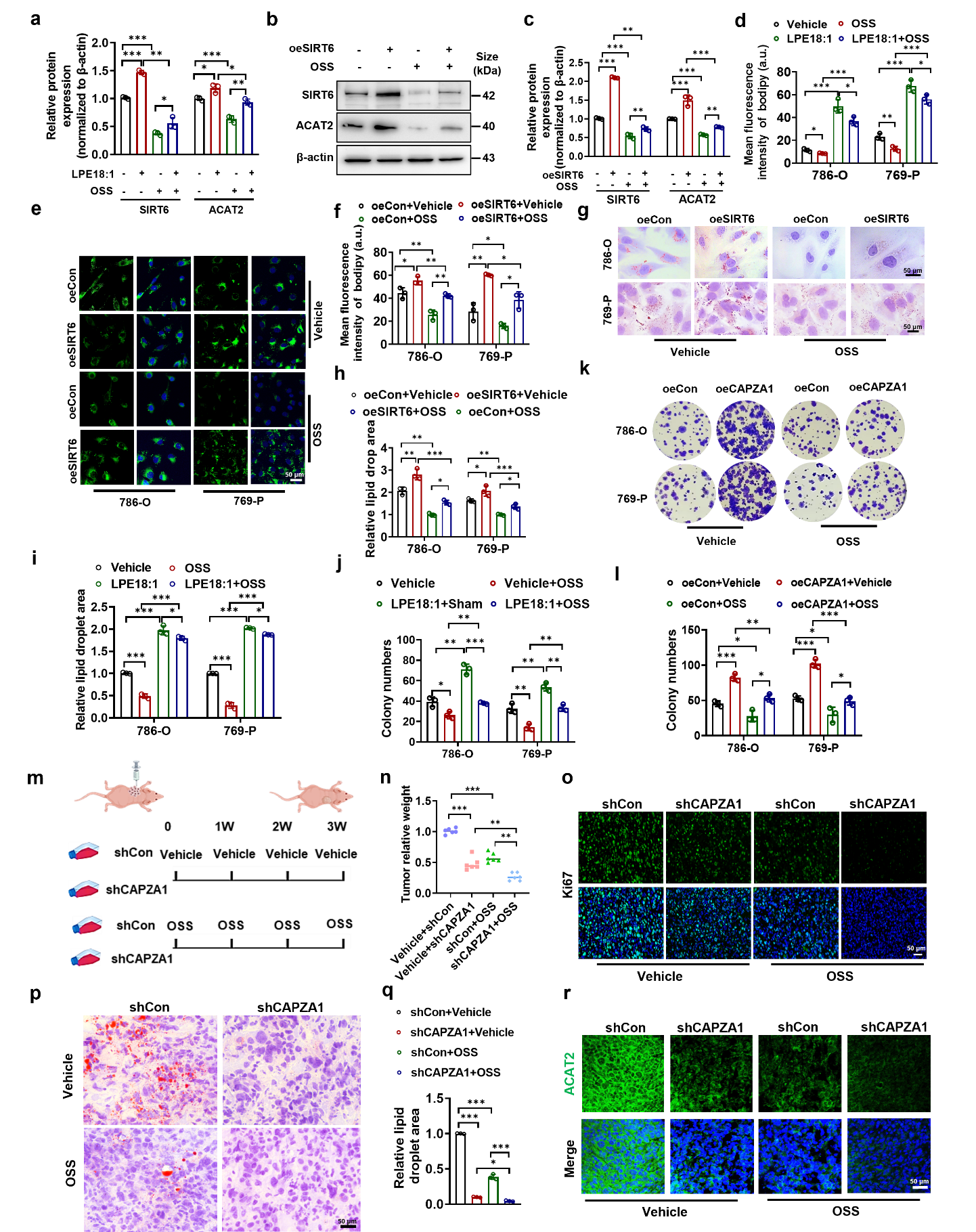
**

**Supplementary Fig. 9.** Pharmacological inhibition of SIRT6 suppresses LPE18:1 induced effects in vitro and in vivo. **a** Quantification of Western blot results from Fig.7f. **b, c** Western blot analysis of SIRT6 and ACAT2 expression in 786-O cells transfected with oeSIRT6 or empty vector and treated with or without OSS-128167 (OSS, 100 μM, 24 h). **d** Quantification of BODIPY fluorescence intensity. **e-h** Lipid accumulation detected by BODIPY and Oil Red O staining in 786-O and 769-P cells overexpressing SIRT6 and treated with or without OSS-128167 (OSS) (100 μM, 24h) Scale bars = 50 μm. **i** Quantification of Oil Red O staining. **j** Quantification of colony numbers from proliferation assays. **k, l** Colony formation assay evaluating cell growth in 786-O and 769-P cells overexpressing CAPZA1 and treated with or without OSS-128167 (OSS) (100 μM, 24h). **m** Schematic of the xenograft tumor model using CAPZA1-knockdown 786-O cells treated with or without OSS-128167 (OSS). **n** Final tumor weights from each treatment group at the endpoint (21 days). **o** Ki-67 immunofluorescence staining of xenograft tumor sections. Scale bars = 50 μm. **p, q** Lipid accumulation detected by Oil Red O staining in xenograft tumor sections. Scale bars = 50 μm. **r** ACAT2 immunofluorescence staining in xenograft tumor sections. Scale bars = 50 μm. Data are presented as mean ± SD (n ≥ 3 per group). *P < 0.05, **P < 0.01, ***P < 0.001 compared to corresponding controls.

**
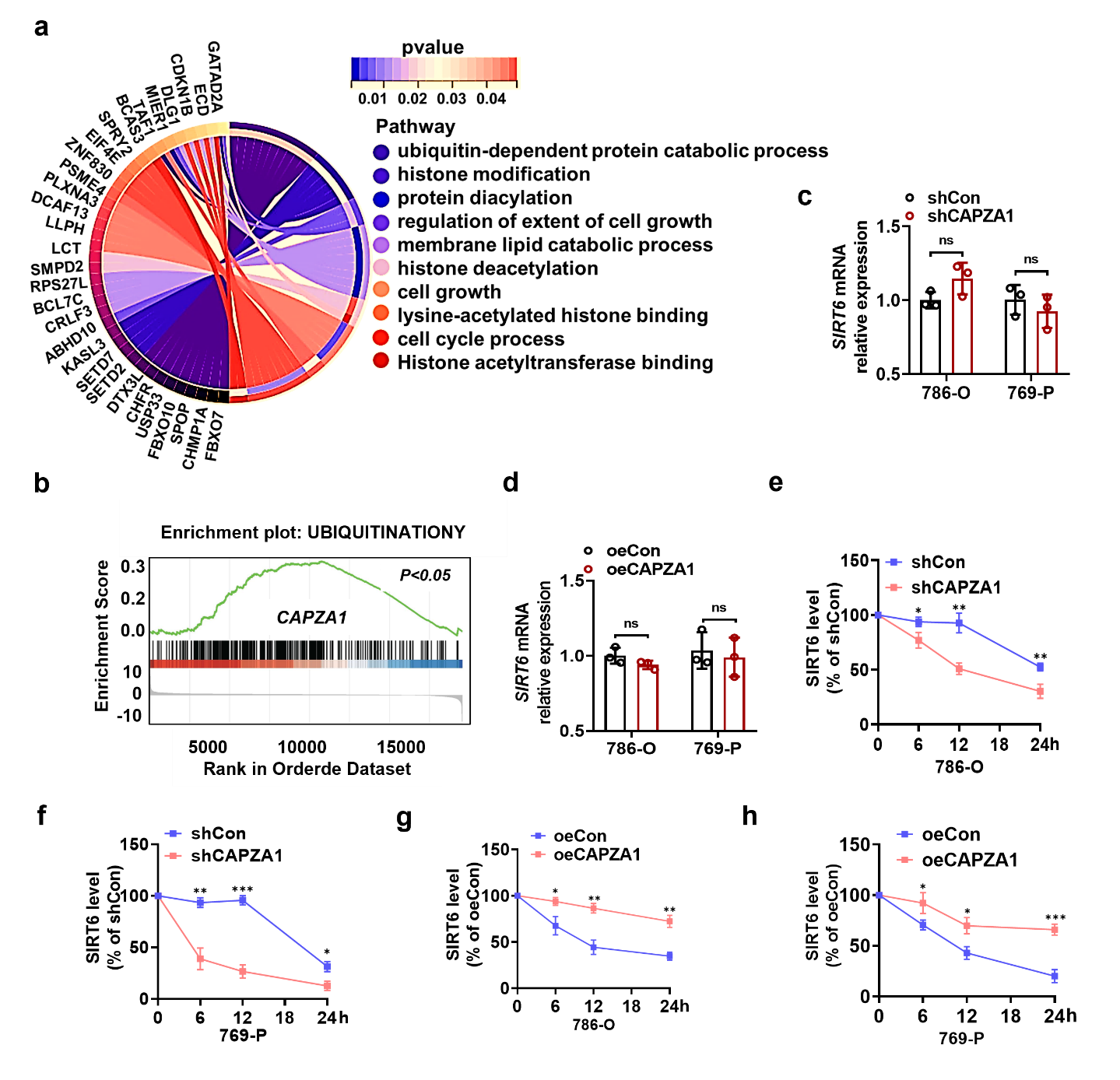
**

**Supplementary Fig. 10.** CAPZA1 does not affect SIRT6 transcription but regulates its protein stability. **a** GO enrichment analysis of ubiquitination-related pathways from proteomic data after CAPZA1 knockdown. **b** GSEA showing enrichment of ubiquitination related signaling associated with CAPZA1 expression in TCGA-KIRC data. **c, d** SIRT6 mRNA levels measured by RT-qPCR in CAPZA1-overexpressing or -knockdown cells. **e h** Quantitative analysis of SIRT6 protein stability from Western blot data at indicated time points in CHX chase assays. Data are presented as mean ± SD (n ≥ 3 per group). *P < 0.05, **P < 0.01, ***P < 0.001 compared to corresponding controls.

**
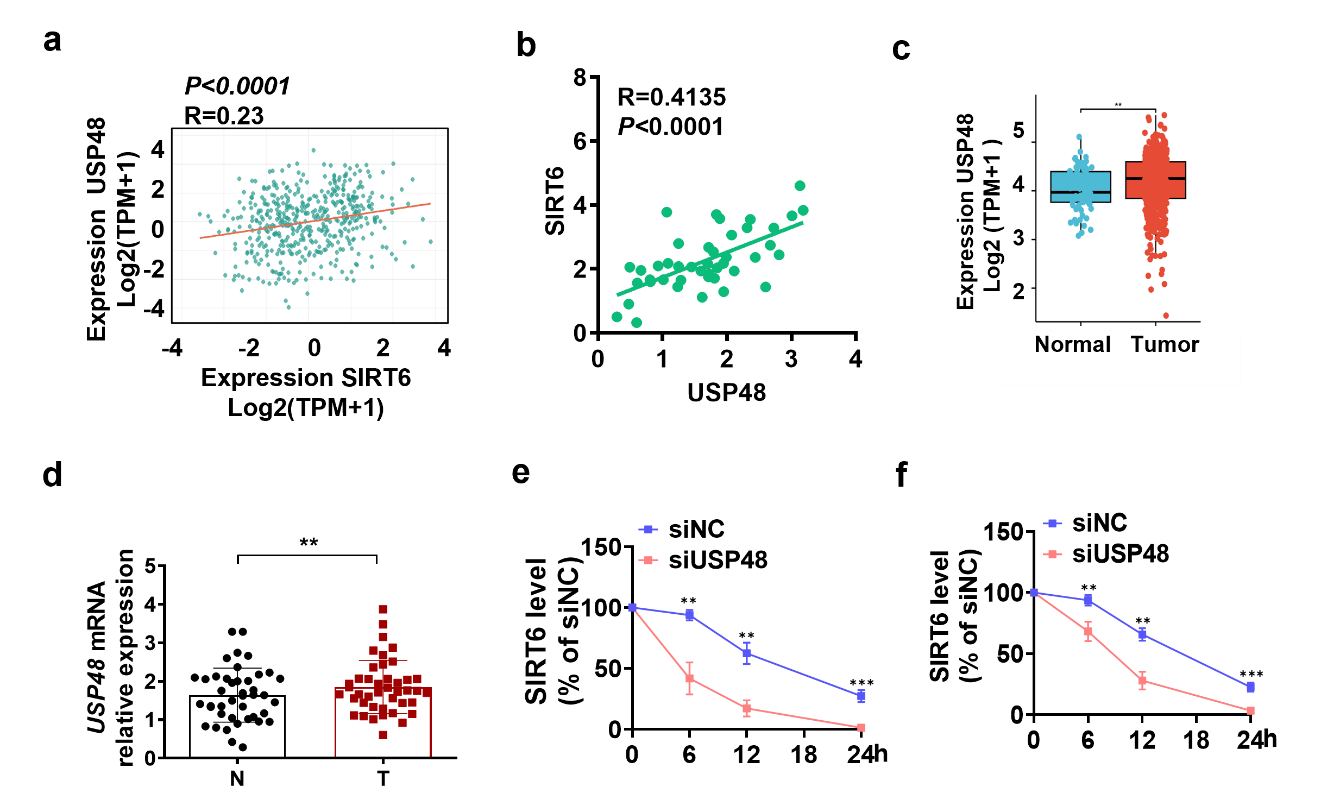
**

**Supplementary Fig. 11.** Supplementary validation and clinical relevance of USP48 in ccRCC. **a, b** Correlation between USP48 and SIRT6 expression in TCGA-KIRC (a) and clinical specimens (b). **c, d** USP48 mRNA in TCGA-KIRC tumors vs. normals (c) and clinical specimens (d). **e, f** Quantitative analysis of SIRT6 protein stability from Western blot data at indicated time points in CHX chase assays in 786-O and 769-P cells. Data are presented as mean ± SD (n ≥ 3 per group). *P < 0.05, **P < 0.01, ***P < 0.001 compared to corresponding controls.

**S****upplementary table 1 The significantly different metabolic molecules in lipid tissues (A: SAT; T: PAT)**

|  | **A2493415** | **A2504598** | **A2469128** | **T2493415** | **T2504598** | **T1092496** |
| --- | --- | --- | --- | --- | --- | --- |
| **2-phenylethanol** | 31800.0599 | 34211.08217 | 4948.652708 | 51047.48204 | 51389.78819 | 52898.58979 |
| **Betaine** | 694281.4852 | 578111.2175 | 630279.8929 | 1238200.423 | 1061227.72 | 1264954.455 |
| **Carnitine** | 1479380.478 | 1361248.248 | 1640820.45 | 2221310.637 | 2327356.813 | 2113111.51 |
| **D-glutamine** | 119224.1805 | 91782.30823 | 95018.29754 | 175230.0635 | 200333.2211 | 166413.6201 |
| **D-pyroglutamic acid** | 307578.5637 | 229824.8614 | 231594.8767 | 483860.7622 | 531997.9312 | 427048.127 |
| **DL-Glutamic acid** | 21675.72069 | 12860.83926 | 13550.06081 | 62630.03873 | 49986.47563 | 39939.66177 |
| **Glycerophosphocholine** | 2575193.681 | 1447405.31 | 2100506.091 | 2603775.385 | 3869343.97 | 3642266.279 |
| **L-Norleucine** | 33977.02889 | 59430.10836 | 37298.21562 | 97748.79414 | 76132.45293 | 61154.82177 |
| **N-acetylglutamine** | 29722.42443 | 32098.4461 | 31381.64536 | 92237.12131 | 100896.0811 | 66384.90878 |
| **Lpe 18:1** | 36993.03663 | 31475.43452 | 30309.61248 | 45223.22726 | 49958.62278 | 33550.4313 |
| **D-fructose** | 27907.31056 | 139039.6394 | 35134.01317 | 185280.0699 | 219575.793 | 122097.324 |
| **D-Mannose** | 76641.59521 | 36140.57688 | 88970.42562 | 112418.5584 | 116298.5193 | 89088.86834 |
| **Glutamine** | 35683.24015 | 103089.0418 | 45787.07185 | 262037.0946 | 213387.1124 | 84734.86222 |
| **Glycerophosphate(2)** | 30655.62081 | 16292.21895 | 28553.33146 | 30675.67027 | 59208.0535 | 38861.50821 |
| **Myo-inositol** | 761753.8375 | 348631.2738 | 784995.322 | 1012982.711 | 1063024.869 | 812694.4621 |
| **Pc(18:0e/8-hete)** | 18477.92414 | 21211.61875 | 27342.44286 | 30200.34928 | 37226.31153 | 49019.79845 |
| **Pc(18:1e/12-hete)** | 25150.57152 | 28064.45901 | 47023.55389 | 51424.53461 | 48373.21351 | 84894.64019 |
| **Sm d42:2** | 118257.0871 | 160567.5974 | 159287.9507 | 190443.2493 | 216023.4546 | 265072.3436 |
| **Sm d42:3** | 62637.82993 | 83210.69468 | 68781.97736 | 118392.1968 | 111322.1679 | 110443.2099 |
| **Gln-glu** | 4273.716635 | 1050.724958 | 1789.457342 | 8162.557028 | 10047.93399 | 8281.456137 |
| **Glu-Leu** | 714.8115397 | 412.7421427 | 279.8225384 | 1874.740026 | 1485.807075 | 648.6879588 |
| **Glutamic acid** | 34428.75873 | 27820.04355 | 29964.48601 | 177113.6031 | 148571.4422 | 48856.39828 |
| **16:0-20:4PE** | 5861382.066 | 6019359.336 | 8297494.229 | 3972449.934 | 4885944.367 | 2631793.373 |
| **Pi(18:0/12-hepe)** | 20703.13088 | 17700.97352 | 18698.76568 | 6422.347072 | 13410.03646 | 7069.994328 |
| **Myristamine oxide** | 154862.0684 | 118764.7504 | 130674.2142 | 108997.9548 | 97662.90557 | 100479.1393 |
| **Asp-Leu-Arg** | 1166.218637 | 1178.469349 | 1001.245655 | 1962.765757 | 2290.848785 | 1041.85448 |
| **Asp-Ser-Arg** | 1257.205143 | 723.5809755 | 2046.724099 | 1320.615455 | 1740.007068 | 1099.046034 |
| **Atorvastatin** | 134.5222321 | 563.3909908 | 15648.54671 | 351.0472854 | 110.9201257 | 563.3909908 |
| **Carbenoxolone** | 363.1846183 | 494.9632481 | 1192.141738 | 3540.177029 | 603.9926795 | 174.1777709 |
| **Carvedilol** | 2557.769965 | 2566.421733 | 23042.43436 | 2497.878769 | 2138.662606 | 2140.604593 |
| **Cefazolin** | 78748.05634 | 64686.70788 | 2640.380376 | 146788.5767 | 136654.2724 | 86833.25578 |
| **Cellobiose** | 1520.608353 | 1665.714866 | 1361.573446 | 1702.840072 | 2499.779808 | 1568.418072 |
| **Chaetoglobosins** | 558.2087188 | 630.7254759 | 513.6151849 | 932.7943106 | 206.3226438 | 509.4722745 |
| **Chaulmoogric acid** | 61921.46163 | 96245.32158 | 33698.72811 | 69709.15406 | 60481.076 | 83215.32287 |
| **Demissidine** | 91061.50394 | 60922.24964 | 91390.24948 | 90968.9693 | 78210.05938 | 79094.32925 |
| **Dendroamide a** | 99.0659255 | 92.8302582 | 1015.657864 | 216.481682 | 329.1181937 | 422.0818425 |
| **Deoxyguanosine** | 1767.821699 | 3162.711057 | 3243.001368 | 4207.288748 | 2588.411305 | 5247.836262 |
| **Descladinoseazithromycin** | 13768.07929 | 351.7328314 | 3714.057385 | 13715.05492 | 330.9647574 | 1615.277678 |
| **Di(2-nonyl) phthalate** | 888.5363353 | 862.4308903 | 1011.792105 | 954.8814375 | 947.460066 | 1242.944391 |
| **Diallyl sulfide** | 7770.318388 | 7842.510178 | 9313.470021 | 7892.747032 | 11239.82277 | 9371.749239 |
| **Diaveridine** | 14311.96063 | 7554.337908 | 43328.41368 | 8707.711723 | 2665.637677 | 6373.652679 |
| **Dibucaine** | 22172.9768 | 23205.30955 | 182637.6304 | 24691.80287 | 21674.17563 | 14856.60615 |
| **Diethanolamine** | 95842.75271 | 119206.693 | 87752.04751 | 137369.9408 | 175681.3133 | 125674.7054 |
| **Samidin** | 517.4099764 | 151.0803002 | 151.5261302 | 1018.055658 | 183.7157954 | 2402.798846 |
| **Securinine** | 2051.259982 | 1711.30876 | 2432.735393 | 1156.437949 | 2405.697925 | 2555.865606 |
| **Ser-Pro** | 24789.26004 | 18882.46474 | 13466.77988 | 17109.94616 | 20260.07194 | 21562.25583 |
| **Ser-Thr** | 11465.47078 | 17783.65008 | 13710.24262 | 38481.03586 | 42115.68418 | 19832.87934 |
| **Sibiricose a6** | 1814.296306 | 266.301511 | 765.290934 | 6106.055268 | 184.7738806 | 9499.97772 |
| **Siduron** | 62174.76183 | 27938.62192 | 73698.69724 | 114678.2451 | 126244.5466 | 188247.1293 |
| **Silybin** | 583.6834864 | 798.7060309 | 186.6815918 | 328.2551486 | 8505.998699 | 1532.0629 |
| **Sofalcone** | 1636.522892 | 1700.588334 | 16810.40397 | 1530.331075 | 1184.790775 | 1022.802255 |
| **Solamargine** | 3132.000318 | 2319.552328 | 389888.2362 | 4108.351627 | 3527.458841 | 2797.091635 |
| **Solanidine** | 1235.710286 | 2328.30253 | 259.123304 | 1235.710286 | 2519.283651 | 1235.710286 |
| **Arg-gly** | 867.63197 | 1128.607074 | 1549.810189 | 6349.803249 | 2168.52132 | 1350.348145 |
| **Cholesterol** | 41057.80479 | 37144.6545 | 50157.76422 | 43766.6929 | 22251.70315 | 37172.53859 |
| **Creatinine** | 511520.6022 | 395584.7518 | 346875.2364 | 827588.1261 | 618804.5 | 523380.6585 |
| **Curcumin** | 47798.64085 | 20419.51158 | 53503.95751 | 104591.9271 | 38960.26543 | 12440.77684 |
| **D-Pipecolinic acid** | 14063.01245 | 11876.29904 | 502.8509655 | 18471.25002 | 20171.59654 | 15876.02575 |
| **Daunorubicin** | 32655.52069 | 35623.22773 | 10092.15266 | 30889.25531 | 133284.0853 | 82218.7364 |
| **Glu-Ala-Arg** | 1382.479537 | 1679.608974 | 1789.619679 | 2125.316255 | 2666.563112 | 1709.814924 |
| **Glu-Arg** | 1644.018443 | 643.2263073 | 742.1851577 | 3848.929198 | 2309.972401 | 1678.640727 |
| **Glu-Glu-Lys** | 882.4032508 | 1074.623272 | 930.1197531 | 1737.724669 | 951.4255131 | 841.0121828 |
| **1-Methylnicotinamide** | 7778.767886 | 6945.818695 | 14699.99415 | 24767.20377 | 28777.67532 | 38414.53229 |
| **2-sdahma [dmed-fahfa]** | 1625.200511 | 1299.840049 | 622.2494973 | 2764.886374 | 2169.945707 | 777.8982434 |
| **4-ketopimelic acid** | 104510.4376 | 22695.74116 | 17104.38125 | 105099.6816 | 39927.22826 | 88717.87541 |
| **Arg-glu** | 2198.814876 | 1484.533988 | 1128.482238 | 2748.417984 | 4759.312681 | 2533.918193 |
| **Betulinic acid** | 28967.57871 | 29578.62428 | 347463.5662 | 35329.11437 | 17837.63642 | 12594.89849 |
| **DL-Lysine** | 15651.6002 | 3358.550634 | 9056.462093 | 32832.13757 | 22907.69931 | 25159.08675 |
| **Gly-Pro-Arg** | 14579.36528 | 8820.47687 | 9493.22319 | 23157.00198 | 9344.222865 | 11688.78966 |
| **Gly-Pro-Lys** | 1775.451358 | 2738.533799 | 528.5905507 | 6408.499034 | 2967.193509 | 2047.571919 |
| **Iridin** | 1889.103308 | 3152.0016 | 592.2113417 | 403.7339003 | 12466.94311 | 6645.156473 |
| **Lithospermic acid** | 615.2994183 | 316.6787295 | 487.7923269 | 1754.522032 | 1645.095399 | 892.911962 |
| **Melezitose** | 8233.177544 | 10396.41762 | 3776.004239 | 4006.307051 | 90354.03507 | 20412.91222 |
| **Melibiose** | 2429.958224 | 1047.440204 | 607.9051288 | 2276.097609 | 3223.882448 | 2676.223529 |
| **Meperidine** | 15304.83021 | 28145.73571 | 29270.22181 | 30943.81996 | 52413.78426 | 7766.224475 |
| **Mesaconitine** | 1178.009523 | 1258.002157 | 1101.275739 | 2047.949631 | 1733.174021 | 1362.1146 |
| **Pro-Thr** | 32563.93674 | 37641.20225 | 30239.26527 | 53561.79521 | 77226.82622 | 171540.1628 |
| **Val-Arg** | 1065.381659 | 671.3155654 | 430.0235252 | 2294.765391 | 1245.829135 | 1379.582529 |

**Supplementary table 2 The significantly different metabolic molecules of blood (N: Renal vein; T: Renal artery)**

|  | **N2207660** | **N2269369** | **N2278006** | **T2207660** | **T2269369** | **T2278006** |
| --- | --- | --- | --- | --- | --- | --- |
| **(-)-Medicarpin** | 1205.962142 | 565.0582729 | 974.2192527 | 3146.027223 | 3345.159508 | 3276.468864 |
| **(+)-.alpha.-tocopherol** | 362540.434 | 387227.0775 | 199160.1053 | 866366.5796 | 723035.516 | 547824.2261 |
| **(phenoxymethyl)penicilloic acid** | 2728.8723 | 4510.88106 | 5865.673056 | 999.8502202 | 2473.648955 | 2230.341948 |
| **1,3-diphenylguanidine** | 1968.813563 | 2032.987767 | 1642.457865 | 1563.041783 | 1578.344101 | 1239.765428 |
| **16-Hydroxypalmitic acid** | 85985.79705 | 72712.15664 | 87712.96015 | 44854.65783 | 52828.15823 | 49081.11705 |
| **3'-sialyllactose** | 4577.749586 | 2012.522721 | 1923.237828 | 2716.82035 | 398.5880615 | 1490.515769 |
| **5-aminoimidazole-4-carboxamide** | 24641.08345 | 24119.55274 | 32465.67272 | 5491.602078 | 10805.25214 | 3872.641729 |
| **Adenosine 5'-diphosphate** | 34519.4131 | 22453.93909 | 9984.123973 | 51414.78523 | 37591.20793 | 44949.1489 |
| **Arg-Ala** | 5828.380535 | 7022.18526 | 7139.933593 | 3650.954649 | 921.5050305 | 3662.671216 |
| **Bis(4-methoxyphenyl)methanol** | 7642.964616 | 6146.28837 | 8826.169938 | 6319.83814 | 4588.439429 | 7248.773405 |
| **Cytochalasin e** | 12101.59578 | 9914.405595 | 9073.264199 | 16963.44322 | 14559.63791 | 17511.47295 |
| **EDTA** | 2074432.763 | 2754635.417 | 2284534.766 | 3131699.077 | 3469300.048 | 3018328.465 |
| **Erucamide** | 3907990.314 | 2045054.873 | 1339765.988 | 7201140.395 | 6345331.394 | 3935371.848 |
| **Herbimycin a** | 1856.976581 | 703.3858693 | 629.374831 | 2628.012028 | 1463.956952 | 1820.520044 |
| **Hypoxanthine** | 1099153.084 | 2604524.514 | 3070248.478 | 391591.4854 | 742618.749 | 316729.7045 |
| **Isopropyl dodecylfluorophosphonate** | 22930.5558 | 14048.01752 | 19721.55515 | 13111.36372 | 8732.132957 | 15583.84928 |
| **Kaempferitrin** | 7658.97263 | 16331.12006 | 12469.7957 | 21334.89499 | 21895.71027 | 20612.52757 |
| **L-Anserine** | 6914.667145 | 7775.946999 | 8210.701957 | 6443.403657 | 6141.541106 | 7218.683665 |
| **Lpe 18:1** | 35221.96633 | 18658.97123 | 198555.2614 | 967852.013 | 847161.1518 | 1025435.79 |
| **Narirutin** | 5440.546172 | 7231.364095 | 4587.220628 | 11873.72144 | 10282.02428 | 11276.82867 |
| **Oligomycin b** | 8285.641604 | 7833.579392 | 10391.35093 | 20786.33447 | 15794.50472 | 14141.10091 |
| **P-tolyl-.beta.-d-glucuronide** | 6463.3264 | 4795.807674 | 6410.517892 | 4153.52814 | 1382.488581 | 4732.129994 |
| **Palatinose** | 7346.45127 | 5905.727613 | 8108.733299 | 3378.296668 | 4054.985871 | 5907.405631 |
| **Pro-Val** | 2851.270975 | 1788.298903 | 4078.132547 | 7995.626525 | 3268.201761 | 8852.806714 |
| **Pteryxin** | 5162.200298 | 2339.127143 | 3697.32817 | 492.8078711 | 913.1122297 | 693.6356974 |
| **Pyrocatechol** | 5457.540662 | 3810.132232 | 6946.209474 | 527.1998659 | 2026.724949 | 905.9558937 |
| **Specnuezhenide** | 25232.68446 | 10279.21758 | 43691.09212 | 157692.1811 | 133736.3002 | 90306.86313 |
| **Taxinine m** | 39661.43919 | 14244.85761 | 56941.25276 | 216024.2357 | 193899.0241 | 99719.29302 |
| **Thioetheramide-PC** | 5124739.628 | 2144966.149 | 3412.62933 | 15671080.07 | 6118705.098 | 8796620.745 |
| **Triphenyl phosphate** | 81649.25186 | 59931.49935 | 59152.74852 | 52198.6563 | 48256.6914 | 38648.93292 |
| **Trp-Asn-Arg** | 2454.267241 | 1597.357056 | 909.7253089 | 2582.834331 | 1791.61648 | 1053.397188 |
| **Val-Asn** | 2950.764985 | 3103.427337 | 2207.503676 | 513.9764318 | 777.6771104 | 614.1032051 |
| **Arg-gly** | 563777.9713 | 116625.997 | 66957.34926 | 149733.5286 | 179253.3611 | 608603.4346 |
| **Calcimycin** | 12260.72157 | 32440.26534 | 1778.158504 | 5730.849221 | 30814.35566 | 2253.017813 |
| **Cholesterol** | 193882.6955 | 908097.2752 | 728903.2707 | 669679.416 | 967822.822 | 1288436.241 |
| **Creatine** | 234211.237 | 326874.7523 | 115152.388 | 359008.9995 | 320743.6045 | 310914.8417 |
| **Creatinine** | 14651197.22 | 11485442.17 | 4281256.266 | 8775994.751 | 8608217.001 | 7468571.929 |
| **Curcumin** | 836399.0872 | 1498972.353 | 595293.1996 | 2199847.609 | 1726172.703 | 1824045.793 |
| **D-Pipecolinic acid** | 71879.40694 | 30754.48499 | 26528.0615 | 39390.67311 | 35600.79469 | 64972.12046 |
| **D-pyroglutamic acid** | 907490.9228 | 833521.4911 | 284019.1772 | 894402.1048 | 835586.439 | 799290.1754 |
| **Daunorubicin** | 4066.926021 | 3938.388987 | 14892.04875 | 4982.827522 | 8077.639087 | 12365.288 |
| **DL-arginine** | 1232334.953 | 3223.673062 | 3542.047049 | 1412.335194 | 2488.707564 | 15656.8339 |
| **DL-tyrosine** | 125460.9221 | 248244.6017 | 9856.525906 | 345511.2893 | 281769.7246 | 193108.4057 |
| **Glu-Ala-Arg** | 8330.203549 | 11651.72922 | 4130.258717 | 10731.72953 | 6808.623799 | 6514.121135 |
| **Glu-Arg** | 120874.3234 | 47290.36207 | 16817.38529 | 106890.6664 | 51828.21983 | 89186.06994 |
| **Glu-Asn-Arg** | 1030.084584 | 1236.035325 | 182.4473158 | 881.4535846 | 452.9998166 | 598.3261998 |
| **2-hydroxybenzyl alcohol** | 28901.09614 | 24424.35178 | 4026.728292 | 16926.97376 | 22126.1184 | 16342.3295 |
| **Acetylcarnitine** | 3674150.58 | 7405119.903 | 3906521.801 | 9616313.315 | 6085121.606 | 6230174.751 |
| **Acetylcholine** | 1819963.461 | 875780.4835 | 178294.8669 | 945204.5529 | 812462.1667 | 662425.6512 |
| **Aniline** | 61979.87433 | 65690.93521 | 69106.74514 | 53646.9263 | 66647.71195 | 48734.70082 |
| **Arg-Asp-Arg** | 9730.889319 | 2486.562737 | 1338.073413 | 3950.175994 | 2404.959037 | 5936.503842 |
| **Arg-Pro** | 64633.04191 | 17743.02717 | 14235.09079 | 15759.71056 | 29525.72674 | 70044.61135 |
| **Asn-Arg** | 48997.56564 | 43037.80474 | 26761.01739 | 50577.08479 | 40066.23617 | 33958.70985 |
| **Auranofin** | 442.4256792 | 738.7448473 | 7151.534257 | 2902.259115 | 1467.520948 | 2265.700088 |
| **Bufotalin** | 14898.38578 | 10271.65402 | 33251.25613 | 2206.409602 | 91226.95928 | 6403.298383 |
| **Cyclohexylamine** | 96048.13375 | 21514.33108 | 30041.09778 | 16276.64478 | 22987.33645 | 4445.4944 |
| **Dl-2,4-diaminobutyric acid** | 47053.5837 | 36925.69473 | 20317.1527 | 38830.81439 | 34966.69559 | 43235.36853 |
| **Ergothioneine** | 17063.44495 | 146671.4453 | 2490.633034 | 15439.85222 | 114511.8474 | 39300.7079 |
| **Fumagillin** | 10374.54469 | 9798.700998 | 20410.91502 | 10989.51968 | 9900.700901 | 10113.62154 |
| **Glu-Glu-Lys** | 8237.947422 | 1969.122069 | 2052.624626 | 1940.074487 | 2958.976528 | 3549.890433 |
| **Indoleacetic acid** | 49408.95952 | 40308.68473 | 22592.87851 | 42997.84312 | 38616.19156 | 48530.4659 |
| **Indoleacrylic acid** | 28374.14602 | 27368.37181 | 8390.066402 | 26859.50323 | 27793.94448 | 19392.38871 |
| **L-Tryptophan** | 1364535.401 | 1394490.532 | 60135.38006 | 1071108.273 | 1864923.374 | 1431591.692 |
| **L-tryptophanamide** | 434.0521575 | 2159.948627 | 758.0057944 | 4953.012492 | 1635.480856 | 1303.525244 |
| **Lys-Asn** | 9343.262046 | 2699.288129 | 1020.338302 | 3411.522437 | 439.4609835 | 6245.458473 |
| **Lys-Pro** | 1769.234264 | 781.7570907 | 9694.009468 | 1100.367207 | 10367.02959 | 969.0891104 |
| **Pyrrolidine** | 40940.47227 | 18573.86294 | 14363.23486 | 24386.59697 | 17377.07451 | 89648.89799 |
| **Sphingomyelin (d18:1/18:0)** | 112790.6282 | 9769.218367 | 89093.58785 | 12537.37211 | 10046.43412 | 12228.93288 |
| **Val-Ile** | 6098.881508 | 4741.834063 | 2171.023845 | 5720.648721 | 4810.15572 | 13769.68632 |

| **Supplementary table 3 The significantly different metabolic molecules in ccRCC tissues** | | | | | | | | | | |
| --- | --- | --- | --- | --- | --- | --- | --- | --- | --- | --- |
|  | **Normal-1** | **Normal-2** | **Normal-3** | **Normal-4** | **Normal-5** | **ccRcc-1** | **ccRcc-2** | **ccRcc-3** | **ccRcc-4** | **ccRcc-5** |
| **Lpe 18:1** | 1133283.141 | 679487.0043 | 1157351.151 | 1241550.194 | 633917.6933 | 39770.40428 | 44965.66128 | 50565.68094 | 120523.6451 | 89854.74477 |
| **Dl-octopamine** | 777599.7404 | 577373.9037 | 730844.7488 | 563264.9825 | 629416.2249 | 254213.1136 | 395879.6123 | 204782.7946 | 288331.3614 | 120415.3469 |
| **Ser-Asn-Arg** | 15370.73967 | 11285.24803 | 17652.47685 | 10546.57228 | 15219.7844 | 45101.50176 | 62751.92945 | 52203.33727 | 43529.10797 | 36951.86885 |
| **L-Tryptophan** | 25744.83342 | 25515.44932 | 35287.47466 | 28139.57716 | 34293.14194 | 13390.86397 | 16798.99277 | 5065.652778 | 11272.15518 | 11467.83208 |
| **D-glutamine** | 2017888.826 | 2138442.846 | 2416877.393 | 1478328.349 | 1628918.236 | 2780010.031 | 3579400.524 | 3409374.956 | 3250861.029 | 2815109.899 |
| **Mesaconitine** | 6715.948604 | 6271.389594 | 8425.981066 | 2895.924182 | 4827.270307 | 16650.44931 | 28362.06513 | 23422.67289 | 24459.77128 | 13496.67184 |
| **Acetylcarnitine** | 14011173.94 | 11970366.05 | 21932556.43 | 14170001.06 | 15634342.39 | 30584692.72 | 34186728.64 | 31679866.5 | 41667889.97 | 24032951.45 |
| **2-hydroxybenzyl alcohol** | 76958.8206 | 46538.54403 | 69666.11586 | 60873.09408 | 61382.82516 | 34394.13678 | 49981.14654 | 26632.10354 | 31163.14576 | 20561.49634 |
| **2-dhahpa [dmed-fahfa]** | 21018.70948 | 29437.38553 | 29442.01436 | 27169.24194 | 25086.49877 | 110954.358 | 50829.39656 | 82907.89699 | 72760.61645 | 132546.6524 |
| **Phenylacetaldehyde** | 1073408.536 | 650053.7813 | 898611.0799 | 800970.0924 | 798630.684 | 442287.8069 | 674504.3096 | 340195.7308 | 447151.7207 | 247083.4151 |
| **2-sdahma [dmed-fahfa]** | 42914.73303 | 37037.43889 | 36516.5825 | 47446.07402 | 60924.11521 | 7920.881575 | 9462.732112 | 13366.15888 | 16460.85056 | 32486.58315 |
| **Dl-2,4-diaminobutyric acid** | 31470.78756 | 33739.6619 | 38334.57132 | 25018.5632 | 26907.58418 | 40285.31672 | 51449.30849 | 49845.78169 | 50258.53942 | 42204.70067 |
| **Ser-Ala-Lys** | 5217.584258 | 4138.984589 | 10067.33186 | 4120.987491 | 5088.863853 | 26317.76359 | 25157.62038 | 17999.72295 | 16370.86323 | 11412.51816 |
| **Asp-Asp-Arg** | 13906.71938 | 11463.97652 | 16833.09185 | 15390.0709 | 18152.66878 | 37962.55908 | 40039.96647 | 22197.37406 | 32665.41675 | 25925.46344 |
| **1,5-pentanediamine** | 2099292.917 | 1637952.132 | 2101663.42 | 1689426.989 | 2087734.35 | 1049666.615 | 1670897.393 | 793967.2889 | 1129721.29 | 641726.1632 |
| **Benzoylhypaconine** | 14281.26846 | 3187.516305 | 11916.88106 | 5613.406021 | 5113.733886 | 25741.12609 | 38889.76605 | 18857.15128 | 32165.53362 | 23766.64028 |
| **Indoleacrylic acid** | 26967.58216 | 18905.76835 | 29923.68048 | 29440.16956 | 30796.69171 | 12107.45157 | 22970.17128 | 7799.601451 | 13426.29967 | 6107.244483 |
| **L-methionine** | 633339.3512 | 481422.073 | 788778.968 | 527438.3791 | 603729.8707 | 214600.156 | 528018.8763 | 162669.6376 | 218772.4357 | 142510.3058 |
| **Ala-Ala-Lys** | 4735.01843 | 2500.20545 | 14095.35125 | 2196.333961 | 2995.890297 | 35058.84913 | 24923.37452 | 20693.35843 | 13267.01423 | 19143.89483 |
| **Albiflorin** | 12785.01648 | 18845.43784 | 23025.12091 | 15233.83161 | 15766.55337 | 6285.635756 | 3042.385618 | 10053.68294 | 3110.551341 | 9926.117118 |
| **Dapsone** | 4511.061515 | 8944.766132 | 9964.916767 | 5757.995012 | 5627.437998 | 35911.67781 | 47607.82064 | 31957.25393 | 13545.08012 | 24993.86013 |
| **L-tryptophanamide** | 33465.23618 | 13182.50929 | 88390.81373 | 23667.33141 | 50922.47053 | 283489.1762 | 445606.2793 | 309159.395 | 548895.2081 | 123541.9452 |
| **D-threonine** | 259073.9216 | 39134.10248 | 107107.4682 | 175278.1027 | 248552.58 | 44856.25182 | 10365.92194 | 11180.79398 | 58637.32044 | 16689.18743 |
| **Bufotalin** | 7229.039882 | 5649.655263 | 8674.463422 | 7120.426005 | 5000.79732 | 19062.24129 | 29311.99802 | 10133.21595 | 35167.4568 | 19340.47893 |
| **Lys-Pro** | 6893.983185 | 5554.397367 | 7487.747359 | 4168.596255 | 16497.55246 | 123113.6465 | 195082.8145 | 73407.39573 | 59803.37744 | 55845.89952 |
| **Licofelone** | 25308.6624 | 16113.15106 | 12145.29525 | 33469.11655 | 11201.69214 | 2313.952527 | 972.8097208 | 11687.08133 | 6230.135391 | 2374.62522 |
| **Melezitose** | 5115.204893 | 9705.703052 | 3229.580943 | 5959.975097 | 5371.392922 | 240889.8689 | 14900.72882 | 158231.1093 | 90908.31023 | 272317.4722 |
| **Decanoyl-l-carnitine** | 10100.19333 | 35603.72743 | 32372.04031 | 11586.60009 | 10143.40581 | 249669.7922 | 490460.4644 | 112000.6512 | 281781.7351 | 137448.1636 |
| **Phe-thr** | 45365.21696 | 103892.3318 | 57638.92107 | 35193.03569 | 45092.96763 | 10054.3606 | 29272.56738 | 4896.118017 | 8946.607774 | 3221.165967 |
| **Pro-Asn-Lys** | 7997.458866 | 6555.674572 | 11210.33209 | 6492.011451 | 5220.539417 | 13234.30562 | 18285.69374 | 19405.74023 | 9696.045289 | 12380.62502 |
| **Siduron** | 1137930.283 | 681624.9428 | 2338269.65 | 1579873.984 | 1097978.8 | 5592561.004 | 11195837.42 | 7817949.68 | 11936942.17 | 1873578.825 |
| **L-glutarylcarnitine** | 1722377.819 | 1669464.998 | 684325.7239 | 1553525.817 | 683032.0287 | 318974.5223 | 41652.66767 | 365707.2497 | 974647.7358 | 139661.6934 |
| **Vinblastine** | 6329.145521 | 4391.856795 | 7311.74875 | 4816.227275 | 5073.57079 | 16242.83145 | 6864.709302 | 10534.27642 | 12247.4007 | 22197.96475 |
| **Arg-glu** | 87244.18278 | 74795.99336 | 178280.3265 | 88855.848 | 88734.90672 | 193501.6804 | 428991.8398 | 213911.803 | 195862.2188 | 279938.643 |
| **Hamamelitannin** | 12472.98885 | 6688.083244 | 9606.562826 | 6418.913194 | 14490.81988 | 3192.02431 | 9413.053454 | 2407.220978 | 2373.397607 | 984.3584228 |
| **Dl-laudanosine** | 3968746.166 | 5939342.996 | 12686490.62 | 4502410.896 | 10379335.03 | 1889056.693 | 1650219.508 | 2624216.804 | 977509.1204 | 2042647.588 |
| **Fumagillin** | 3346.003043 | 4097.661995 | 3488.830006 | 3337.37627 | 2415.545037 | 22642.82216 | 38129.72536 | 23540.35875 | 15350.45358 | 4860.572055 |
| **NAD+** | 89361.37801 | 74873.84022 | 74492.44155 | 90409.61449 | 58715.03943 | 9340.429502 | 18807.79829 | 8220.898467 | 6115.159279 | 3344.560624 |
| **Ht-2 toxin** | 81198.28865 | 66537.32274 | 113309.9957 | 57760.53564 | 120534.915 | 318668.7219 | 408651.2894 | 215161.0232 | 208908.3218 | 110054.4661 |
| **Aniline** | 29773.09068 | 15544.70396 | 50157.40967 | 25415.41729 | 18423.96432 | 87033.60888 | 167043.5991 | 115142.2565 | 177451.5186 | 30963.19472 |
| **1-Methylnicotinamide** | 69200.50083 | 36748.23505 | 25006.70306 | 18647.50004 | 72290.38719 | 239141.4862 | 734554.2459 | 535241.1123 | 445464.9949 | 84078.78644 |
| **Glu-Glu-Arg** | 7964.304249 | 8271.562722 | 3627.894077 | 2391.948482 | 8561.772496 | 27347.03567 | 29652.23634 | 12550.25653 | 7593.980321 | 17922.67678 |
| **DL-Lysine** | 1043356.173 | 882783.0098 | 820886.3626 | 910862.5842 | 933113.3755 | 890419.728 | 603796.4148 | 773114.0332 | 749755.2749 | 735540.1694 |
| **Gln-Glu-Lys** | 20724.20498 | 11419.71309 | 25154.6436 | 14544.22056 | 13966.25109 | 49155.0329 | 62738.05863 | 30833.22014 | 43980.60872 | 18737.67029 |
| **Urea** | 553581.2928 | 569410.5486 | 330087.1899 | 652168.9523 | 463434.046 | 364587.9176 | 255258.6437 | 363098.308 | 402805.7723 | 321041.3747 |
| **Cyclohexylamine** | 5759.967687 | 5115.390394 | 5360.769553 | 562.1819889 | 5883.355823 | 12552.5764 | 17821.7089 | 11180.6657 | 25739.87614 | 6028.379768 |
| **Thr-Pro-Arg** | 20069.80055 | 18531.76091 | 34746.29005 | 15918.81942 | 20818.1591 | 80508.1624 | 67921.6002 | 31245.46481 | 39923.78552 | 34295.91352 |
| **Cytochalasin a** | 11359.96773 | 9952.634117 | 7941.209068 | 9897.477779 | 10709.03972 | 47027.7593 | 47252.20242 | 13382.35085 | 22443.01931 | 20376.34537 |
| **Trp-Asp-Arg** | 2015.519655 | 6556.420587 | 8079.829956 | 968.0126143 | 1559.627798 | 15661.92357 | 20691.23127 | 11687.69725 | 16646.83515 | 2396.757099 |
| **Ser-Gln** | 10511.87987 | 6149.666792 | 13970.56936 | 9175.299292 | 4996.778323 | 2111.739162 | 8575.748154 | 2465.716853 | 3302.102462 | 1636.291916 |
| **Laminaritetraose** | 4447.374784 | 5985.849839 | 4872.828363 | 4084.505714 | 2885.624649 | 35246.72775 | 3777.221576 | 14761.12243 | 15230.09794 | 37349.86997 |
| **Val-Pro-Lys** | 8763.061624 | 6136.831994 | 16942.58495 | 5850.194161 | 5834.201548 | 52223.26254 | 25201.34057 | 20702.11369 | 24388.07828 | 13385.58246 |
| **Trimethylamine n-oxide** | 90978.05203 | 394645.2174 | 397950.0953 | 142289.9522 | 439441.6222 | 33086.66362 | 108417.0333 | 117404.397 | 36341.75037 | 51298.48021 |
| **Cabergoline** | 12500.34097 | 8664.055758 | 21805.54144 | 5316.161328 | 10879.65781 | 75298.38204 | 60423.81439 | 17712.54504 | 38492.60666 | 21509.89738 |
| **Asn-Ala-Arg** | 14531.3439 | 6488.248405 | 18156.95489 | 6460.267367 | 7847.936312 | 1740.071304 | 9536.199534 | 1347.382058 | 2671.236415 | 2031.866313 |
| **Tetramisole** | 79158.09147 | 95230.52156 | 94632.43485 | 102830.1297 | 91392.68701 | 72651.71492 | 49058.36284 | 39924.37947 | 65355.61197 | 91578.47739 |
| **Ser-Asp** | 21122.33531 | 13148.34317 | 9810.031272 | 8294.23122 | 11323.17573 | 4530.798365 | 12329.94283 | 4518.80734 | 4275.551639 | 4036.482307 |
| **Gly-Lys-Arg** | 6009.119735 | 5397.580154 | 9512.482311 | 6326.790271 | 7186.064209 | 36736.86358 | 21400.85992 | 8231.579547 | 16795.33745 | 14305.24052 |
| **Val-Glu** | 49586.80446 | 29955.92793 | 50168.39163 | 31684.83542 | 28334.56114 | 13323.13895 | 35373.11822 | 26785.41536 | 21779.13206 | 10948.63457 |
| **Niacinamide** | 43179482.4 | 35829562.82 | 35690071.25 | 40956303.67 | 37527057.67 | 19146973.71 | 42791465.04 | 27331287.06 | 24888108.91 | 10161165.75 |
| **Halofantrine** | 1846.169134 | 3300.736867 | 3769.083242 | 2838.528278 | 2864.001962 | 12122.94518 | 5516.444255 | 14765.16669 | 16255.41609 | 38244.08976 |
| **3-aminobutanoic acid** | 42073.80904 | 38260.83423 | 62085.69051 | 27233.0606 | 22112.4086 | 7943.273543 | 34237.6658 | 13251.03611 | 19795.52113 | 2719.919947 |
| **Acetylcholine** | 2765661.452 | 3203378.275 | 3437727.172 | 2008711.878 | 2640434.991 | 2948594.842 | 6901069.991 | 5259999.577 | 7338292.56 | 3864004.681 |
| **Lauroyl-l-carnitine** | 41789.22928 | 42210.64834 | 148088.046 | 26114.4347 | 69315.92812 | 331330.268 | 683038.9698 | 249918.6942 | 805709.0522 | 102234.4054 |
| **Sulfometuron methyl** | 85925.08195 | 77988.61788 | 46893.94699 | 68594.73292 | 125972.1695 | 15538.05003 | 47299.2025 | 20353.74541 | 79053.9422 | 13865.02323 |
| **Picolinic acid** | 92779.88025 | 85978.12537 | 72563.51828 | 91230.88944 | 86724.44756 | 44226.20343 | 93948.66191 | 64850.7151 | 55150.40781 | 22048.08019 |
| **Oleoyl-l-carnitine** | 1153428.089 | 663404.5454 | 2032928.753 | 449971.5873 | 785131.5829 | 3577581.491 | 5398668.117 | 2898620.023 | 4758606.543 | 508766.144 |
| **Gamabufotalin** | 11025.15315 | 5423.653001 | 27779.92588 | 8557.548765 | 7322.787608 | 253.7135199 | 4418.775473 | 1081.329022 | 1188.595423 | 728.7637411 |
| **Hexanoyl-l-carnitine** | 419341.7016 | 601770.8158 | 643903.796 | 495710.9859 | 568171.9003 | 2014918.469 | 3588992.298 | 1093883.372 | 3995951.592 | 694676.4887 |
| **Salicin** | 15820.05409 | 7174.123001 | 26060.83976 | 12764.53827 | 24347.14494 | 5055.609213 | 16848.95145 | 3603.497148 | 4213.024336 | 2695.069584 |
| **3,4-dihydroxy-l-phenylalanine** | 22294.80352 | 10556.5103 | 12449.68741 | 51161.5883 | 24328.82892 | 6980.219438 | 5743.917037 | 6343.8657 | 7030.682007 | 5515.46698 |
| **Taurocholate** | 14923.11785 | 5120.035073 | 5176.766151 | 16745.79383 | 21924.58517 | 4533.844833 | 2367.669862 | 2259.409202 | 9645.322705 | 2243.904381 |
| **Pro-pro** | 19488.24317 | 20938.47368 | 27727.35126 | 19740.27275 | 35961.26752 | 53657.89536 | 164756.2506 | 62128.94705 | 86569.3574 | 45833.76236 |
| **L-.beta.-homoserine** | 84187.30654 | 87349.34895 | 143205.9795 | 50917.56196 | 41441.36134 | 9824.477978 | 68416.45834 | 26688.89453 | 34176.00663 | 17797.6491 |
| **Pro-hyp** | 700925.9961 | 358049.6963 | 1842956.132 | 517966.8168 | 669845.0964 | 212265.0902 | 68540.77814 | 305369.1059 | 125889.3085 | 64735.44096 |
| **Cysteine** | 17813.57709 | 28556.8753 | 17992.53585 | 15150.57021 | 31892.73604 | 6306.849666 | 25388.95302 | 3673.320067 | 8082.27135 | 388.2076022 |
| **Nelfinavir** | 8249.090394 | 11056.74142 | 17475.113 | 4821.067714 | 10472.30729 | 25845.53239 | 20595.05523 | 15668.39269 | 18937.69289 | 10191.3909 |
| **Glucosamine** | 17694.8979 | 11559.29808 | 18772.9702 | 10860.26895 | 14200.73634 | 32673.578 | 52124.87555 | 48577.2262 | 46731.91216 | 5751.051307 |
| **Tyr-Gly** | 5720.52943 | 8379.152574 | 12792.53602 | 8010.904521 | 8486.919467 | 20971.90624 | 39148.91862 | 11660.25785 | 10603.71556 | 31580.39672 |
| **Ranaconitine** | 6302.658444 | 5198.867717 | 7796.662682 | 4703.730459 | 4483.12105 | 53732.86265 | 8759.763649 | 10853.46503 | 16731.16033 | 49454.93899 |
| **Pro-Gln-Arg** | 7623.647432 | 6624.447448 | 11459.8857 | 6502.381317 | 3917.976455 | 14303.19255 | 30529.32592 | 10134.05986 | 31619.01528 | 11043.53032 |
| **Asp-Pro** | 9008.346671 | 10943.56658 | 14858.5476 | 24282.20514 | 17064.53994 | 25730.29285 | 45036.38665 | 30824.84954 | 28286.21469 | 14683.33347 |
| **Asn-Lys** | 48630.30837 | 29755.95169 | 83590.89631 | 42518.61387 | 44524.08146 | 86457.42983 | 159574.181 | 69323.61395 | 75013.46311 | 87848.11371 |
| **Latrunculin a** | 20727.6649 | 7439.683415 | 17648.78219 | 8236.106302 | 18967.11062 | 43483.86192 | 29561.85841 | 27273.82322 | 26708.5458 | 12826.05163 |
| **Octanoylcarnitine** | 49843.68808 | 184875.4441 | 69675.8701 | 64575.66772 | 69272.1022 | 570221.9594 | 1688426.354 | 288946.8903 | 809961.7516 | 254264.2085 |
| **Celaxanthin** | 10695.04933 | 40339.31672 | 62555.62729 | 49873.38321 | 42252.01928 | 84070.3584 | 198568.5152 | 114620.2396 | 107462.2991 | 33427.27912 |
| **D-ornithine** | 31456.11929 | 42486.40293 | 33072.70189 | 29359.05267 | 36906.79459 | 60448.37179 | 104950.3257 | 51382.38186 | 70141.28774 | 31624.3602 |
| **Lys-Pro-Lys** | 6954.539481 | 24333.43207 | 30279.03943 | 16458.15899 | 4360.755341 | 228611.9409 | 59421.71167 | 24910.05721 | 51978.64443 | 101121.1922 |
| **Sphingomyelin (d18:1/18:0)** | 82876.24427 | 53623.98664 | 76808.65339 | 49232.90198 | 57782.08963 | 236941.4772 | 458922.0052 | 172591.7879 | 178276.5877 | 63807.14816 |
| **4-ketopimelic acid** | 528936.5505 | 551615.3529 | 444207.9797 | 302240.9304 | 459210.5894 | 764931.204 | 1147781.278 | 774774.7781 | 324209.3959 | 926295.7771 |
| **N-nervonoyltaurine** | 8050.590901 | 7097.131728 | 10422.5021 | 4345.342786 | 6456.564436 | 18089.92003 | 102538.1296 | 74727.77099 | 57692.31961 | 3501.04782 |
| **Glu-Ala-Lys** | 14236.26197 | 38460.17505 | 114211.0434 | 13553.2607 | 14131.705 | 108195.9236 | 147673.5318 | 176916.4171 | 139068.9909 | 12443.75489 |
| **Nivalenol** | 42898.10014 | 49891.57128 | 71863.18705 | 35181.67724 | 62814.10922 | 106369.958 | 110339.5453 | 102258.3594 | 59131.23518 | 48234.62336 |
| **Pyrrolidine** | 14521.38875 | 11286.59241 | 36135.62676 | 36836.9877 | 56568.63798 | 8787.249709 | 14342.72673 | 13767.22931 | 12894.39782 | 12382.26652 |
| **Ala-Thr-Lys** | 5925.476856 | 4530.234091 | 12611.77805 | 7328.802099 | 7212.717896 | 20485.00617 | 17308.47791 | 8888.548115 | 14037.62453 | 7628.888729 |
| **L-Leucine** | 61462.7975 | 51272.19174 | 75867.95249 | 59878.03367 | 50774.00169 | 34753.90909 | 69613.12358 | 37240.41122 | 42212.62116 | 28003.4915 |
| **2(1h)-pyridinone** | 126140.3195 | 113405.4318 | 120949.0581 | 129049.3529 | 119520.7275 | 73070.74822 | 139182.4537 | 99154.76658 | 87677.08542 | 34769.52313 |
| **Pyrimethanil** | 7786.245345 | 11699.23711 | 7429.475595 | 7498.179936 | 10000.12688 | 11280.54309 | 42803.32404 | 23996.84066 | 31811.95705 | 8704.367079 |
| **Leu-Glu-Arg** | 29212.23786 | 17351.97737 | 127057.3578 | 38225.21678 | 76306.839 | 126009.568 | 228009.5599 | 93506.80458 | 216137.3507 | 60154.72344 |
| **7-Oxocholesterol** | 70826.35597 | 57060.56712 | 160672.3117 | 52823.80871 | 66548.14256 | 189784.2068 | 372336.4215 | 173107.8928 | 344105.4324 | 43042.98282 |
| **Urobilin** | 7599.729072 | 29184.62511 | 24205.5244 | 29895.93582 | 6601.793349 | 6180.344608 | 11120.49033 | 4844.630984 | 11189.13789 | 1062.048523 |
| **L-palmitoylcarnitine** | 2295431.502 | 2042127.803 | 5954590.099 | 1595140.881 | 2295162.452 | 7160981.583 | 13338550.58 | 5464199.388 | 12928622.16 | 1591595.055 |
| **Pro-Thr** | 543594.9978 | 629135.8812 | 772725.8149 | 1651076.119 | 1781097.91 | 2466380.427 | 802816.7862 | 4730127.092 | 7254945.457 | 2236863.916 |
| **Methyltyrosinate** | 26412.62697 | 27154.22345 | 59053.32705 | 20135.44618 | 17085.68244 | 2950.937435 | 27605.90579 | 9823.245569 | 11657.95955 | 9101.93938 |
| **Gly-Pro-Lys** | 7447.063998 | 11728.68683 | 11303.93052 | 7853.072641 | 10446.50298 | 65110.94889 | 3374.60217 | 146404.5356 | 50568.9787 | 31079.73493 |
| **Iridin** | 1012.693619 | 1540.315231 | 1059.930974 | 2434.158418 | 7119.218513 | 23584.49063 | 1562.872513 | 10137.05246 | 7048.794132 | 41017.13794 |
| **Epothilone b** | 7409.963536 | 4727.466671 | 18516.47659 | 10713.47394 | 14578.48211 | 45831.49277 | 22558.29257 | 14873.73462 | 21153.2573 | 13555.21457 |
| **Amikacin** | 7198.333469 | 9566.277158 | 35627.91702 | 6483.408545 | 8210.419051 | 32658.8098 | 128883.5551 | 57880.73522 | 160492.7281 | 5880.515462 |
| **Leu-Phe** | 26059.87168 | 13417.02724 | 32153.08279 | 14102.95477 | 14802.71831 | 8616.320437 | 20668.32644 | 10411.24365 | 9160.886705 | 9524.889321 |
| **Gly-Pro-Arg** | 30300.26972 | 17388.70656 | 32471.91231 | 17137.14107 | 23075.37587 | 65025.18468 | 206370.0546 | 49378.17847 | 88200.97691 | 41039.23193 |
| **Ser-Glu-Lys** | 6231.645704 | 10862.28138 | 18432.43298 | 7088.654096 | 8095.564428 | 16097.86104 | 34425.05408 | 17109.98518 | 19777.00913 | 10672.17521 |
| **Lewis x trisaccharide** | 17662.77765 | 6274.575045 | 2594.677925 | 15750.40686 | 16773.70092 | 7424.975364 | 4369.95547 | 6845.008674 | 6346.431291 | 4906.675458 |
| **Loganin** | 10994.39296 | 6101.291188 | 10442.20106 | 13548.66105 | 12314.86207 | 5927.734201 | 14048.73538 | 3186.913055 | 5578.632469 | 1351.847534 |
| **DL-arginine** | 26327785.79 | 22210958.5 | 30787386.98 | 23202439.02 | 24546771.96 | 31589082.58 | 38946225.96 | 32160163.62 | 27565939.8 | 25719893.53 |
| **Myristoyl-l-carnitine** | 216597.8568 | 153682.3436 | 683816.724 | 168637.561 | 221455.5215 | 879200.0175 | 2857919.355 | 693062.0205 | 2808186.394 | 165072.7528 |
| **Cystine** | 160502.744 | 74713.88018 | 79305.87532 | 107514.5182 | 88187.73022 | 126820.4421 | 425242.3125 | 120476.2565 | 183610.198 | 293993.0622 |
| **Ergothioneine** | 42001.72425 | 111248.2631 | 126946.0338 | 329290.1899 | 157090.5332 | 241616.0202 | 1454983.176 | 640119.9897 | 1898225.57 | 131044.8192 |
| **Theanine** | 13299.79936 | 15946.39712 | 10794.49509 | 15191.90077 | 10499.95597 | 5432.626455 | 331460.9835 | 142187.221 | 268525.2924 | 3634.361201 |
| **L-carnitine** | 25822301.37 | 24485638.53 | 28383043.58 | 21943336.01 | 24564621.42 | 24916650.28 | 33445950.13 | 32636337.9 | 35071050.42 | 24361794.72 |
| **Arg-Asp-Arg** | 16118.47881 | 15690.78502 | 29093.5451 | 11554.39858 | 12607.85758 | 23755.40729 | 43771.00877 | 73813.21123 | 45185.40809 | 9841.673275 |
| **Arg-Pro** | 15701.54561 | 13355.10309 | 19638.6536 | 7143.688359 | 31584.38315 | 103863.0171 | 559786.5636 | 116289.0501 | 110211.8754 | 103907.1008 |
| **Etizolam** | 8392.837481 | 5921.146956 | 8317.237504 | 4309.325618 | 5850.34398 | 43992.90621 | 300707.7211 | 169623.4968 | 21364.98951 | 32062.02215 |
| **N-acetylsalicylamide** | 94973.81595 | 56266.90456 | 147223.3218 | 369.2707176 | 5416.927242 | 27375.62807 | 2139.074111 | 11395.62642 | 1358.766498 | 1082.983365 |
| **Celastrol** | 2821.153018 | 3598.379256 | 4796.100503 | 3641.482627 | 3195.953539 | 12200.91773 | 8140.626713 | 10089.29008 | 6952.309642 | 44527.75144 |
| **Arginine** | 923273.6281 | 805514.7116 | 1103616.642 | 771831.2348 | 908424.6617 | 1120822.451 | 1261138.308 | 989893.3919 | 854473.907 | 1134923.59 |
| **Chymostatin** | 1544.672035 | 3124.071224 | 9393.403064 | 1970.162908 | 2512.461057 | 5486.515719 | 21220.5401 | 8894.725543 | 17907.86236 | 2282.028098 |
| **Trp-Gln-Lys** | 5699.562677 | 7730.492421 | 11005.71946 | 8938.31887 | 11444.77784 | 25645.31433 | 22432.28502 | 7066.265732 | 15616.02953 | 8214.808256 |
| **3-deoxyaconitine** | 4427.100477 | 10474.15763 | 13988.63974 | 7956.390537 | 5321.925526 | 13750.4047 | 20717.49162 | 66408.82075 | 37896.10691 | 4770.395153 |
| **Beta-octylglucoside** | 77077.16386 | 537859.3729 | 173651.8984 | 150305.1713 | 266037.0016 | 180093.1378 | 813334.18 | 201266.1025 | 752119.0758 | 668055.6331 |
| **Pyroglu-Lys-Arg** | 15401.45557 | 10169.10577 | 21989.71538 | 10817.78194 | 7524.322777 | 25013.29466 | 43041.93219 | 12265.61325 | 33303.21805 | 11959.52875 |
| **Pantoprazole** | 16479.59211 | 18143.08262 | 44156.99237 | 15202.21442 | 18684.17083 | 3742.646115 | 20682.05943 | 21480.59923 | 5944.936996 | 1386.593279 |
| **Val-Arg** | 137992.043 | 73408.46713 | 186514.2853 | 63780.58431 | 63954.38287 | 16750.94946 | 135049.0982 | 46212.29373 | 53236.69248 | 9565.689937 |
| **Pro-Ala-Arg** | 7724.573811 | 7989.339913 | 18509.75054 | 8477.012487 | 4106.038791 | 20107.05397 | 44285.47491 | 15175.35379 | 16506.7943 | 9261.823528 |
| **Morphine n-oxide** | 26052.4542 | 8665.936555 | 29195.54165 | 17156.03775 | 14375.1888 | 4743.62365 | 26787.92265 | 4440.990831 | 11162.38552 | 4431.39329 |
| **Oxindole** | 17916.23759 | 74642.98311 | 17709.67069 | 13554.64024 | 42377.90485 | 18713.13348 | 107675.7844 | 42416.86492 | 82668.25857 | 73803.95425 |
| **Betulinic acid** | 70099.89652 | 17855.57957 | 13413.3422 | 8528.847809 | 5370.897106 | 79086.52004 | 13245.01808 | 13165.59003 | 122185.7518 | 93485.67419 |
| **Auranofin** | 2829.274685 | 3054.75114 | 3249.081481 | 2460.922539 | 3287.408131 | 8087.778545 | 125638.3897 | 70461.47995 | 7289.703816 | 10868.44529 |
| **Lithospermic acid** | 17656.13715 | 7431.107079 | 7733.880899 | 9660.506469 | 15083.09438 | 21703.97017 | 48693.03143 | 14654.229 | 72186.68443 | 5311.28082 |
| **L-propionylcarnitine** | 8342647.352 | 6902430.22 | 5638080.315 | 5277671.896 | 4208003.61 | 2731489.381 | 5953280.079 | 4911795.952 | 6550136.738 | 1498799.609 |
| **Shikimate** | 6797.52339 | 12153.29458 | 5128.684833 | 76032.07613 | 14573.08934 | 1237.286577 | 1318.414779 | 1043.393434 | 4146.286447 | 1155.500786 |
| **Digitoxin** | 15005.72235 | 14817.80791 | 10365.06425 | 13244.05149 | 3522.443122 | 25226.70342 | 7851.330009 | 10738.57702 | 20028.51948 | 23351.77947 |
| **Lys-Asn** | 26934.64539 | 12216.63835 | 65590.94039 | 19548.38236 | 21853.62249 | 52478.2812 | 95659.66496 | 39751.19253 | 40026.53517 | 37085.2277 |
| **Furmecyclox** | 34709.78012 | 42294.97982 | 66021.53173 | 27094.15967 | 27524.08961 | 8154.638941 | 53621.26002 | 14914.14911 | 23134.52982 | 16411.49489 |
| **Glu-Gly-Glu** | 8814.926309 | 4837.709993 | 14989.96114 | 8114.869229 | 7363.578703 | 5026.608258 | 32759.41864 | 22491.3034 | 54521.32691 | 6135.711481 |
| **Val-met** | 30322.88238 | 10194.4658 | 41455.06718 | 15775.36131 | 15425.62562 | 2397.578265 | 31653.67189 | 9926.794632 | 10551.83621 | 3853.766091 |
| **Pyrazoxyfen** | 8722.038961 | 5163.228018 | 8533.423289 | 6978.180131 | 6176.037782 | 13280.85824 | 29187.67818 | 10100.07901 | 21914.74438 | 1343.674178 |
| **6-hydroxyflavone** | 16961.81377 | 3911.713937 | 7570.753641 | 7441.059348 | 6945.137821 | 33298.47401 | 5277.192437 | 16255.22161 | 14228.20414 | 12051.50055 |
| **Calcimycin** | 23236.41806 | 15784.87075 | 25200.9519 | 41360.97328 | 20396.90288 | 33458.07574 | 147194.2096 | 37337.8989 | 64092.9968 | 27177.82233 |
| **Val-Ile** | 213816.9932 | 65621.9514 | 285067.1782 | 80330.42198 | 65178.67092 | 7536.605167 | 160210.1416 | 120044.6562 | 48020.15691 | 6269.713418 |
| **Creatine** | 295308.3979 | 213809.3904 | 283430.4447 | 289385.5787 | 387083.6167 | 231908.9182 | 909676.6605 | 411034.9142 | 472074.9957 | 390864.3547 |
| **Arg-Trp** | 13683.14095 | 10734.14772 | 24407.78903 | 12328.62934 | 19367.74612 | 38504.15128 | 65182.93475 | 13982.78274 | 17362.11643 | 20235.10178 |
| **Asn-Arg** | 55350.01726 | 38270.19165 | 95561.93077 | 58590.08712 | 46193.61254 | 72172.64358 | 237692.5092 | 73889.69298 | 138182.9826 | 49695.16918 |
| **Pro-Cys-Arg** | 18265.27022 | 28222.94087 | 63043.79269 | 11664.20717 | 17627.34839 | 70257.56078 | 60373.91812 | 31046.40005 | 47827.73383 | 19380.74375 |
| **Fulvestrant 9-sulfone** | 9050.209048 | 6814.659465 | 17441.48982 | 5338.673357 | 6088.84523 | 9781.605816 | 15761.14141 | 11789.14733 | 23195.08409 | 9370.653606 |
| **Samidin** | 9706.399989 | 13162.28564 | 27951.4681 | 40311.10241 | 48948.61491 | 3860.643684 | 3608.225848 | 8854.900969 | 12703.10271 | 37903.89574 |
| **3-hydroxyanthranilic acid** | 149852.0277 | 111430.3578 | 146821.8549 | 151125.4993 | 187698.8861 | 107000.4336 | 280428.0108 | 191237.7607 | 236531.8522 | 181928.7838 |
| **Hypoxanthine** | 27082449.07 | 48255223.71 | 20974405.78 | 48842665.73 | 23861761.53 | 24095547.58 | 20987127.63 | 24180783.51 | 24834486.69 | 27544704.34 |
| **His-His** | 18288.88858 | 10163.58404 | 27063.53729 | 8012.248325 | 3782.113354 | 4081.66295 | 16463.93988 | 4724.825286 | 6470.770321 | 3568.137535 |
| **L-cystine** | 139660.6361 | 47178.51334 | 76397.41753 | 95267.97334 | 70029.64857 | 98979.0906 | 228401.0935 | 67881.42418 | 115605.9123 | 178103.5905 |
| **Ala-Ser-Arg** | 21586.09404 | 18321.82576 | 12208.275 | 14672.71792 | 17840.43711 | 17386.29294 | 81308.76043 | 27456.02894 | 42287.91569 | 12556.63013 |
| **Thr-Asn-Lys** | 12228.97479 | 14483.8076 | 21094.04045 | 14340.23997 | 13864.85135 | 220365.4597 | 28453.80606 | 16231.93595 | 14486.61262 | 48891.90297 |
| **Nudifloramide** | 286413.3104 | 267680.5298 | 159221.1362 | 438774.5067 | 171315.9475 | 213154.8338 | 132060.9378 | 205089.7433 | 246221.4434 | 151688.0268 |
| **Met-Met-Arg** | 114568.5493 | 85476.62435 | 107746.3927 | 103657.9184 | 73841.37345 | 161759.3885 | 108306.2251 | 114532.9474 | 152079.7172 | 677052.2311 |
| **Gossypol** | 45013.91619 | 49732.89824 | 36301.14461 | 31775.78038 | 24639.40558 | 49376.72755 | 33264.20224 | 42500.77786 | 41838.81114 | 66954.44739 |
| **Leu-Pro-Lys** | 9900.126952 | 6575.143414 | 20669.63976 | 6267.989199 | 5971.867245 | 29559.65001 | 37475.21073 | 9061.840854 | 15404.07637 | 4820.778212 |
| **Methoxyfenozide** | 10895.76612 | 6984.092666 | 30101.14139 | 23120.01635 | 10544.74821 | 50616.43397 | 36044.14102 | 11952.13163 | 26976.71488 | 12157.503 |
| **Hexaconazole** | 7671.141633 | 5171.229541 | 8086.578088 | 6157.929851 | 6639.834387 | 7059.103317 | 90868.34022 | 32214.8526 | 15277.2876 | 4458.680172 |
| **Stearoylcarnitine** | 192726.7942 | 121909.1171 | 412304.6945 | 188995.169 | 210684.6644 | 408064.2005 | 475695.4315 | 233773.647 | 1168427.631 | 104183.6409 |
| **Arg-Cys** | 8706.210785 | 29743.13259 | 13527.46737 | 8627.2865 | 9012.847677 | 16119.64008 | 118404.7861 | 22734.8916 | 32178.31512 | 16651.05401 |
| **Asp-Asn** | 26944.63013 | 7085.544191 | 23470.83664 | 17401.7107 | 4641.602991 | 7826.886228 | 25203.64048 | 2684.992327 | 6311.725952 | 3582.466694 |
| **Manumycin a** | 16219.79131 | 16953.57267 | 11378.75729 | 20941.8221 | 17863.65463 | 9052.504347 | 9555.153935 | 25570.42565 | 10257.04902 | 5162.279176 |
| **Terfenadine** | 33961.22934 | 27387.29538 | 42227.59195 | 19742.16479 | 18352.29559 | 13157.326 | 139944.8462 | 76606.20704 | 88236.46726 | 3160.444664 |
| **Taurine** | 6201146.441 | 4167172.99 | 5541007.04 | 4923031.41 | 4460466.919 | 2961771.201 | 6234849.279 | 5144627.251 | 4283828.515 | 2170152.662 |
| **Tyr-Thr** | 14296.97326 | 8986.797628 | 23603.47286 | 9015.883434 | 12514.16424 | 3224.518628 | 23970.75974 | 4638.936437 | 5913.890394 | 2151.547112 |
| **Gly-Val-Arg** | 49476.58896 | 14929.42185 | 86139.2451 | 43186.54029 | 39341.61471 | 16452.82957 | 75034.0272 | 12100.89311 | 19318.97729 | 13996.78712 |
| **Ala-Val-Lys** | 5864.43051 | 5243.541493 | 15999.79546 | 3127.75557 | 4587.886623 | 13656.06922 | 38641.59868 | 7276.563397 | 9467.861722 | 7983.922006 |
| **Ile-Glu-Arg** | 11766.18451 | 7178.601505 | 15925.83755 | 8262.361841 | 8354.979038 | 20941.13758 | 89740.78852 | 9669.77346 | 16867.43044 | 14558.27122 |
| **Dl-5-hydroxylysine** | 991060.5871 | 938479.1967 | 1022432.43 | 473026.6448 | 533526.529 | 1060056.209 | 534055.9151 | 385629.121 | 590821.6627 | 175113.7212 |
| **Guanine** | 101614.4104 | 77378.2986 | 202201.5088 | 76260.00521 | 73491.45243 | 117221.3052 | 451134.2339 | 146225.8056 | 270607.8282 | 44712.58285 |
| **Pro-val** | 5359.698058 | 5920.668665 | 14473.37682 | 4108.789909 | 4891.849206 | 8970.599229 | 61042.97306 | 9419.279718 | 10945.71896 | 11784.21369 |
| **Cytidine** | 249310.6307 | 297865.5542 | 170009.4954 | 340330.3898 | 295335.701 | 72820.83995 | 345002.3718 | 183998.168 | 326233.5219 | 17512.18516 |
| **Glu-Pro-Arg** | 13204.5972 | 11456.2666 | 21525.59873 | 11932.04581 | 13379.80334 | 18962.06398 | 37352.25526 | 18091.76548 | 16050.33987 | 11537.14242 |

**Supplementary table 4 The significantly different genes of RNA-Seq in 769 cells**

|  | **LPE(18:1)-1** | **LPE(18:1)-2** | **LPE(18:1)-3** | **sham-1** | **sham-2** | **sham-3** |
| --- | --- | --- | --- | --- | --- | --- |
| **ACAT2** | 1.511337917 | 1.575462286 | 1.479571187 | 0.791924568 | 0.388876103 | 0.00 |
| **CAPZA1** | 13.89778587 | 24.4732565 | 0.687832838 | 0.395093311 | 0.335110497 | 1.790164926 |
| **RAB6D** | 0.044596183 | 0.066411927 | 0.074843688 | 0.026706186 | 0.019671187 | 0.007156241 |
| **ADAMTS5** | 0.05277688 | 0.050014676 | 0.061061668 | 0.025140462 | 0.019752437 | 0.013473374 |
| **SEPTIN7P6** | 0.184454628 | 0.149551757 | 0.140449233 | 0.021478269 | 0.084375567 | 0.046042869 |
| **PCOTH** | 0.102594472 | 0.071298293 | 0.066958696 | 0.035838922 | 0.017598773 | 0.019206934 |
| **SIRPB1** | 0.059795854 | 0.05936469 | 0.064114152 | 0.029840357 | 0.005861266 | 0.025587455 |
| **TRIM80P** | 0.074027963 | 0.060020245 | 0.120786623 | 0.043099827 | 0.033862807 | 0.018478581 |
| **H3C2** | 0.229214655 | 0.28672797 | 0.224396804 | 1.104974985 | 0.707739288 | 0.720917795 |
| **DKKL1** | 0.070719208 | 0.092149681 | 0.121157339 | 0.389088977 | 0.345732868 | 0.238310939 |
| **MAPK8IP1P1** | 0.048837535 | 0.050909657 | 0.031874014 | 0.34120447 | 0.25132368 | 0.109715773 |
| **HSD17B14** | 0.084265244 | 0.131760785 | 0.123741115 | 0.46361771 | 0.390274938 | 0.141979314 |
| **EMC6** | 1.426178333 | 1.644847931 | 1.633852898 | 2.289605747 | 2.373554661 | 5.317235652 |
| **C19orf73** | 0.252568601 | 0.263284802 | 0.123629937 | 0.694801744 | 0.357430596 | 0.602869935 |
| **PSPC1P1** | 0.035226673 | 0.036721301 | 0.068972491 | 0.147667138 | 0.181280589 | 0.158276689 |
| **U2AF1L5** | 0.471927804 | 1.500011826 | 1.452026333 | 0.311314173 | 0.268880248 | 0.357346742 |
| **FAM156A** | 1.145053463 | 0.073909399 | 0.114527945 | 0.152320842 | 0.135000269 | 0 |
| **LDHAP3** | 0.302117649 | 0.072677576 | 0.273016112 | 0.146128938 | 0.0717569 | 0.026104662 |
| **TMEM38A** | 0.0862682 | 0.089928463 | 0.069099491 | 0.238346229 | 0.153363257 | 0.193805487 |
| **GLDCP1** | 0.052484849 | 0.492405502 | 0.085636122 | 0.100838789 | 0.027009319 | 0.039303211 |
| **PSMD7P1** | 0.343234252 | 0.178898648 | 0.084004958 | 0.04496275 | 0.088316185 | 0.048193223 |
| **CBSL** | 0.520701962 | 0.628178207 | 0.658675235 | 1.679971849 | 0.61419892 | 2.60900582 |
| **TMEM225B** | 0.113796089 | 0.033892665 | 0.159148879 | 0.255548178 | 0.250974862 | 0.292169321 |
| **RPL15P5** | 0.147100394 | 0.038335425 | 0.036002125 | 0.154158001 | 0.189248967 | 0.247850859 |

**Supplementary table 5 The significantly different genes of RNA-Seq in 786 cells**

|  | **LPE(18:1)-1** | **LPE(18:1)-2** | **LPE(18:1)-3** | **sham-1** | **sham-2** | **sham-3** |
| --- | --- | --- | --- | --- | --- | --- |
| **CAPZA1** | 1.046464285 | 0.845317388 | 0.969081704 | 0.303354391 | 0.282281882 | 0.170822529 |
| **CHI3L2** | 0.031522024 | 0.04507209 | 0.091447076 | 0.133098306 | 0.243767729 | 0.131890006 |
| **LLPHP3** | 0.532641377 | 0.444268269 | 0.386305277 | 0.937091707 | 1.029762392 | 1.361924008 |
| **TPPP3** | 0.109815047 | 0.076130994 | 0.08688527 | 0.149877028 | 0.299159634 | 0.213492334 |
| **SERF1B** | 0.774477321 | 0.78693958 | 0.748933371 | 0.255542543 | 0.312624522 | 0.237396247 |
| **PSMD7P1** | 0.254571246 | 0.424668198 | 0.246174931 | 0.059716628 | 0.061520731 | 0.118349011 |
| **ADAMTS5** | 0.180321299 | 0.206267411 | 0.209248692 | 0.067678845 | 0.069723495 | 0.05029833 |
| **RPL15P5** | 0.128252839 | 0.125390523 | 0.162183636 | 0.20673933 | 0.940949298 | 0.565665108 |
| **CBSL** | 0.142671797 | 0.12981865 | 0.100339033 | 0.133870244 | 1.667512965 | 0.150744245 |
| **TMEM225B** | 0.079855255 | 0.076121238 | 0.064351212 | 0.187322279 | 0.167250619 | 0.28461997 |
| **MIR4648** | 2.163855593 | 1.718895088 | 1.046243459 | 0.338394227 | 0 | 0.335322199 |
| **LINC02672** | 0.16078184 | 0.122610968 | 0.124383123 | 0.060345224 | 0 | 0.059797395 |
| **PSMC2P1** | 0.19821578 | 0.377894493 | 0.095839095 | 0.092993833 | 0.03832131 | 0.092149612 |
| **SOCS5P2** | 0.133748249 | 0.163921121 | 0.036953411 | 0.017928171 | 0 | 0.071061658 |
| **ATP5F1EP2** | 0.499351291 | 0.634668955 | 0.482881596 | 0.312363902 | 0.160900374 | 0 |
| **DDT** | 0.092078961 | 0.105328039 | 0.142467194 | 0.03455941 | 0.035603487 | 0.017122836 |
| **C5orf46** | 0.213187743 | 0.264184861 | 0.412312693 | 0.760136787 | 1.195259919 | 0.495550048 |
| **SPINK13** | 0.022098951 | 0.021065608 | 0.021370079 | 0.269563402 | 0.106810461 | 0.061642209 |
| **RARRES2P1** | 0.053983923 | 0.10291929 | 0.052203416 | 0.405228846 | 0.10436781 | 0.200775038 |
| **IL17B** | 0.148136813 | 0.141209963 | 0.163715358 | 0.039713748 | 0 | 0.039353217 |
| **GLDCP1** | 0.297485875 | 0.18229853 | 0.143837071 | 0.039876243 | 0 | 0.098785591 |
| **C19orf73** | 0.152254232 | 0.171523007 | 0.053539111 | 0.298710469 | 0.227456179 | 0.411824279 |
| **ACAT2** | 0.447991337 | 0.049274232 | 0.316580635 | 0.064669899 | 0.033311823 | 0.128165618 |

**Supplementary table 6 The significantly different proteins of mass spectrometry**

|  | **NC_1** | **NC_2** | **NC_3** | **shCAPZA1_1** | **shCAPZA1_2** | **shCAPZA1_3** |
| --- | --- | --- | --- | --- | --- | --- |
| **AAMDC** | 1.0895 | 0.8154 | 0.6372 | 4.00E-05 | 4.00E-05 | 4.00E-05 |
| **CRADD** | 0.8476 | 0.7611 | 0.8934 | 4.00E-05 | 4.00E-05 | 4.00E-05 |
| **HADHB** | 14.7846 | 23.7772 | 17.8402 | 41.4666 | 46.7696 | 37.8748 |
| **LPCAT1** | 1.4199 | 1.3354 | 0.9491 | 2.2929 | 3.0083 | 3.1967 |
| **DTX3L** | 0.4509 | 1.2196 | 0.4368 | 2.0183 | 2.4483 | 3.1217 |
| **BCKDHA** | 3.3221 | 1.9143 | 3.9116 | 11.8946 | 13.5464 | 15.541 |
| **LACTB** | 0.1739 | 4.00E-05 | 0.6427 | 1.0725 | 1.4431 | 1.3043 |
| **MIER1** | 0.0839 | 2.2317 | 0.8649 | 11.0469 | 12.9306 | 10.1346 |
| **ISG15** | 4.00E-05 | 4.00E-05 | 4.00E-05 | 0.6433 | 1.2014 | 1.4568 |
| **KCNK1** | 4.00E-05 | 0.4062 | 4.00E-05 | 1.36 | 1.266 | 1.7548 |
| **PARP9** | 0.0855 | 0.0301 | 0.2257 | 1.0206 | 1.5425 | 1.4166 |
| **PROS1** | 0.0339 | 4.00E-05 | 4.00E-05 | 0.3583 | 0.332 | 0.3211 |
| **ENTPD6** | 4.00E-05 | 0.5147 | 4.00E-05 | 10.6528 | 10.4797 | 9.6772 |
| **AP5B1** | 0.0292 | 4.00E-05 | 4.00E-05 | 2.4187 | 2.1958 | 2.2718 |
| **ZNF106** | 4.00E-05 | 4.00E-05 | 4.00E-05 | 0.0144 | 0.0156 | 0.0111 |
| **CCPG1** | 4.00E-05 | 4.00E-05 | 4.00E-05 | 0.0228 | 0.015 | 0.0189 |
| **SIRT6** | 1.2358 | 0.793 | 0.8765 | 4.00E-05 | 4.00E-05 | 4.00E-05 |
| **MAP4K3** | 0.2499 | 0.1906 | 0.1729 | 4.00E-05 | 0.0328 | 4.00E-05 |
| **N6AMT1** | 1.3415 | 1.0601 | 0.6193 | 4.00E-05 | 4.00E-05 | 0.3089 |
| **DDHD2** | 0.1719 | 0.0701 | 0.154 | 4.00E-05 | 4.00E-05 | 0.0612 |
| **METTL5** | 1.2642 | 0.8923 | 0.9828 | 0.6331 | 4.00E-05 | 4.00E-05 |
| **PFKFB1** | 0.0589 | 0.0785 | 0.0726 | 0.0098 | 0.0195 | 0.0165 |
| **PFKFB4** | 0.0539 | 0.0719 | 0.0666 | 0.009 | 0.0179 | 0.0151 |
| **ELOVL5** | 2.8233 | 3.9283 | 3.8407 | 0.705 | 0.2103 | 2.2784 |
| **HMMR** | 1.0431 | 0.6336 | 0.9067 | 0.2322 | 0.3596 | 0.2591 |
| **METTL17** | 0.6461 | 0.7584 | 1.0354 | 0.2079 | 0.5271 | 0.2989 |
| **CAPZA1** | 57.0763 | 55.0952 | 62.6969 | 25.8364 | 23.4595 | 25.2935 |
| **ECHDC1** | 6.7256 | 8.1699 | 7.0865 | 3.0021 | 2.9147 | 4.5969 |
| **ACAT2** | 10.9331 | 7.471 | 8.2748 | 3.3076 | 4.1392 | 5.446 |

**Supplementary table 7**  primers used in the study

| **Name** | **Sequcence 5’ to 3’** |
| --- | --- |
| GAPDH-F | ATGAATGGGCAGCCGTTAGG |
| GAPDH-R | GCATCACCCGGAGGAGAAAT |
| Ucp1-F | AGGTCCAAGGTGAATGCCC |
| Ucp1-R | TTACCACAGCGGTGATTGTTC |
| Prdm16-F | CTTCGGATGGGAGCAAATACTG |
| Prdm16-R | TCCACGCAGAACTTCTCACTG |
| Pgc1α-F | CTGTGTCACCACCCAAATCCTTAT |
| Pgc1α-R | TGTGTCGAGAAAAGGACCTTGA |
| Fabp4-F | ACTGGGCCAGGAATTTGACGAAGT |
| Fabp4-R | TCTCGTGGAAGTGACGCCTTTCAT |
| PPARγ2-F | ACCAAAGTGCAATCAAAGTGGA |
| PPARγ2-R | ATGAGGGAGTTGGAAGGCTCT |
| USP48-F | CACATCGAGACCGCTTACCG |
| USP48-R | ACCAACCAAGCAATTCGGATT |
| CAPZA1-F | TTCATCACTCATGCACCCCC |
| CAPZA1-R | TCACAGGCGTGAACTGATCC |
| KLK2-F | TCAGAGCCTGCCAAGATCAC |
| KLK2-R | GAATCACCCTTTCCCCTCCAG |
| BOLA2-F | CTCCTGTAAGGCAGCAAGGT |
| BOLA2-R | GAAGCTACAGGAGCAACGGT |
| 18S-F | CAGCCACCCGAGATTGAGCA |
| 18S-R | TAGTAGCGACGGGCGGTGTG |
| ACAT2-F | GACTTCGTCTCCTTCGTGCC |
| ACAT2-R | TAGGCCTGACCCACAGATCA |
| RPL15P5-F | CCACAAGCACAGGGAAATGC |
| RPL15P5-R | TGGCACCTGTCTCAGATCAC |
| CBSL-F | CACACCATCGAGATCCTCCG |
| CBSL-R | ATCCTACCTGGCCGACTTCT |
| TMEM225B-F | GCATGCCCTGGAGATCAAGGC |
| TMEM225B-R | GTGGACAACAAGTGCCAGCA |
| ADAMTS5-F | CCAAATGCACTTCAGCCACC |
| ADAMTS5-R | CCAGGATCTGCTTTCGTGGT |
| DLAG5-F | GGTTGTGAGGGTTCCTGCTT |
| DLAG5-R | TGAAGACATCCTGAGCCACC |
| CCDC85B-F | TAGCGCTTAAGGAGCTCTGC |
| CCDC85B-R | AGTGCCTTCAATCATCGGGG |
| HMMR-F | TGGCCGTCAACATGTCCTTT |
| HMMR-R | TGGAGATGGTGCACAACCAG |
| ARMC1-F | GAGCCATCGTCCAGGATCAG |
| ARMC1-R | ATCTGAACGGATTCGCACCA |
| SIRT6-F | AGGATGTCGGTGAATTACGC |
| SIRT6-R | CCAGTTCCCACACCTTCC |
| siSIRT6-F | GUCUCACUUUGUUACUUGUTT |
| siSIRT6-R | AUAAGUAACAAAGUGAGACTT |
| siCAPZA1-F | GGAUUCAACGUGUGGUCAUTT |
| siCAPZA1-R | AUGACCACACGUUGAAUCCTT |
| siACAT2-F | GCAUUUCACAACUGUCAUATT |
| siACAT2-R | UAUGACAGUUGUGAAAUGCTT |
| Control shRNA | TTCTCCGAACGTGTCACGT |
| shCAPZA1-1 | GATGGGCAACAGACTATTATT |
| shCAPZA1-2 | CCAGTATAACATGGATCAGTT |
| shCAPZA1-3 | GTGGTTGGCGTGCTTAAGATT |
| shSIRT6 | GCTACGTTGACGAGGTCATGA |
| shNRF2-1 | CAGTCTTCATTGCTACTAATC |
| shNRF2-2 | AGATATAGATCTTGGAGTAAG |
| ChIP_449-F | GCTCGCACTCGAGATTCTCGCCCGTGGG |
| ChIP_449-R | GGCAAAGCTCCTCCTCGCAGCCCACAGG |
| ChIP_796-F | GCTTGCCTGGAAACCCTAGAAAGCTCAG |
| ChIP_796-R | GGACTGGAATCCCACGGGCGAGAATCTCG |
| ChIP_1247-F | GATAGAATAGAGCAAAAGCCACGGGAGGC |
| ChIP_1247-R | CAGGCAAGCTTATGAGGGGCCACCG |
| ChIP_1650-F | CAGTAGCTGGGATTACAGGCGCACGGCACC |
| ChIP_1650-R | CAAGGAATGCCTCCCGTGGCTTTTGC |
| ChIP_1999-F | GATAAACTAGGAATTACATGGTAAGTTGAAAG |
| ChIP_1999-F | GCCGGGCGTGGTGCCGTGCGCCTGTAATC |
|  |  |
|  |  |
|  |  |
|  |  |
|  |  |
|  |  |
|  |  |

**REFERENCES**

1. Yang, Z. et al. Dysregulation of p53-RBM25-mediated circAMOTL1L biogenesis contributes to prostate cancer progression through the circAMOTL1L-miR-193a-5p-Pcdha pathway. *Oncogene.* **38**, 2516-2532 (2019).

2. Qi, J.C. et al. CDK13 upregulation-induced formation of the positive feedback loop among circCDK13, miR-212-5p/miR-449a and E2F5 contributes to prostate carcinogenesis. *J. Exp. Clin. Cancer Res.* **40**, 2 (2021).

3. Yang, Z. et al. SF3B4 promotes Twist1 expression and clear cell renal cell carcinoma progression by facilitating the export of KLF 16 mRNA from the nucleus to the cytoplasm. *Cell Death Dis.* **14**, 26 (2023).

4. Li, X. et al. Glycolytic reprogramming in macrophages and MSCs during inflammation. *Front. Immunol.* **14**, 1199751 (2023).

5. Jemal, M. High-throughput quantitative bioanalysis by LC/MS/MS. *Biomed. Chromatogr.* **14**, 422-429 (2000).
