# Supplementary material for: Lysophosphatidylethanolamine 18:1 drives clear cell renal cell carcinoma by stabilizing SIRT6 to reprogram lipid metabolism
Source: Signal Transduct Target Ther. 2025 Dec 8;10:398. doi: 10.1038/s41392-025-02496-1 (PMC12682907; doi:10.1038/s41392-025-02496-1)
Supplement: Supplementary file 2 — Original data of Western blot [file 41392_2025_2496_MOESM2_ESM.docx]

**Western blot**

**Fig 3d**







**Kda**

**43**

**35**

**Kda**

**40**

**33**

**CAPZA1 β-actin**







**Kda**

**43**

**35**

**Kda**

**40**

**33**

**CAPZA1 β-actin**

**Fig 4e**







**Kda**

**55**

**52**

**Kda**

**43**

**34**

**CyclinD1 CyclinA2**







**Kda**

**40**

**35**

**Kda**

**43**

**34**

**CDK4 ACAT2**





**Kda**

**55**

**43**

**β-actin**

**Fig 6f**







**Kda**

**43**

**Kda**

**42**

**33**

**IP SIRT6 WB CAPZA1 β-actin**







**Kda**

**43**

**33**

**Kda**

**42**

**IP SIRT6 CAPZA1**





**Kda**

**43**

**β-actin**

**Fig 6h**







**Kda**

**43**

**35**

**Kda**

**42**

**35**

**SIRT6 β-actin**

**Fig 7d**


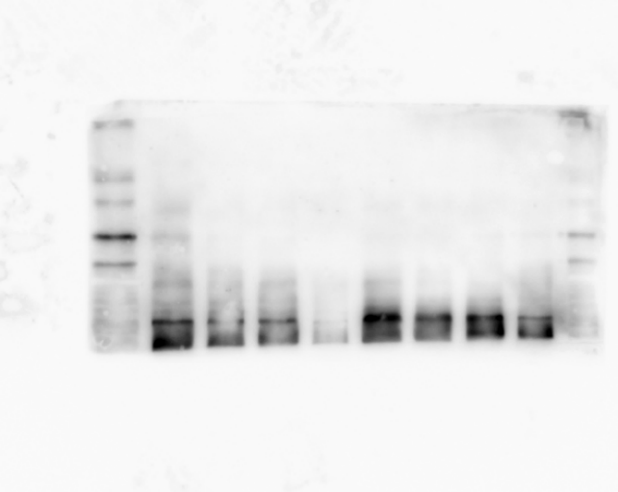




**Kda**

**70**

**40**

**Kda**

**40**

**34**

**ACAT2 CDK4**





**Kda**

**43**

**35**

**β-actin**

**Fig 7f**







**Kda**

**40**

**35**

**Kda**

**42**

**35**

**SIRT6 ACAT2**





**Kda**

**43**

**β-actin**

**Fig 8a**







**Kda**

**43**

**33**

**Kda**

**42**

**35**

**SIRT6 CAPZA1**





**Kda**

**43**

**35**

**β-actin**

**Fig 8b**


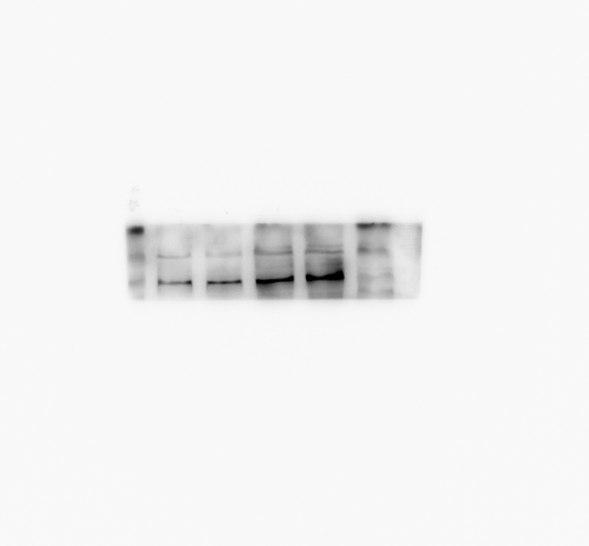

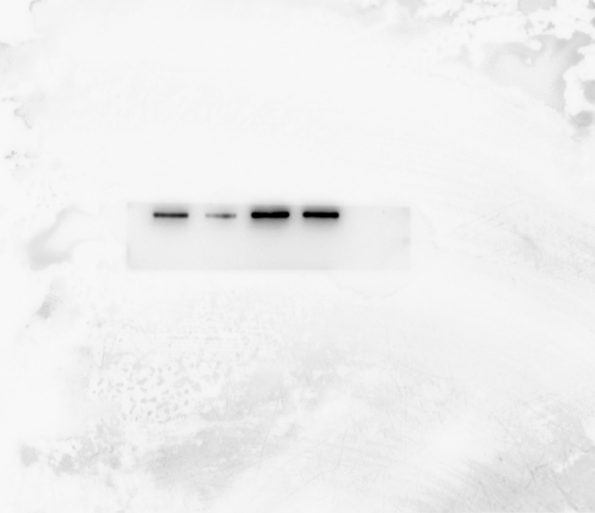


**Kda**

**33**

**25**

**Kda**

**55**

**42**

**SIRT6 CAPZA1**





**Kda**

**55**

**43**

**β-actin**

**Fig 8c**







**Kda**

**55**

**42**

**Kda**

**43**

**33**

**SIRT6 CAPZA1**





**Kda**

**55**

**43**

**β-actin**

**Fig 8d**







**Kda**

**55**

**42**

**Kda**

**43**

**33**

**SIRT6 CAPZA1**





**Kda**

**55**

**43**

**β-actin**

**Fig 8e**







**Kda**

**43**

**33**

**Kda**

**42**

**35**

**SIRT6 CAPZA1**





**Kda**

**43**

**35**

**β-actin**

**Fig 8f**







**Kda**

**40**

**33**

**Kda**

**42**

**SIRT6 CAPZA1**





**Kda**

**43**

**34**

**β-actin**

**Fig 8g**





 **SIRT6 CAPZA1**

**Kda**

**40**

**33**

**Kda**

**42**





**Kda**

**43**

**β-actin**

**Fig 8h**







**Kda**

**42**

**35**

**Kda**

**43**

**33**

**SIRT6 CAPZA1**





**Kda**

**43**

**35**

**β-actin**

**Fig 8i**







**Kda**

**42**

**Kda**

**33**

**SIRT6 CAPZA1**





**Kda**

**43**

**34**

**β-actin**

**Fig 8j**







**Kda**

**55**

**33**

**Kda**

**42**

**35**

**SIRT6 CAPZA1**





**Kda**

**43**

**35**

**β-actin**

**Fig 8k**


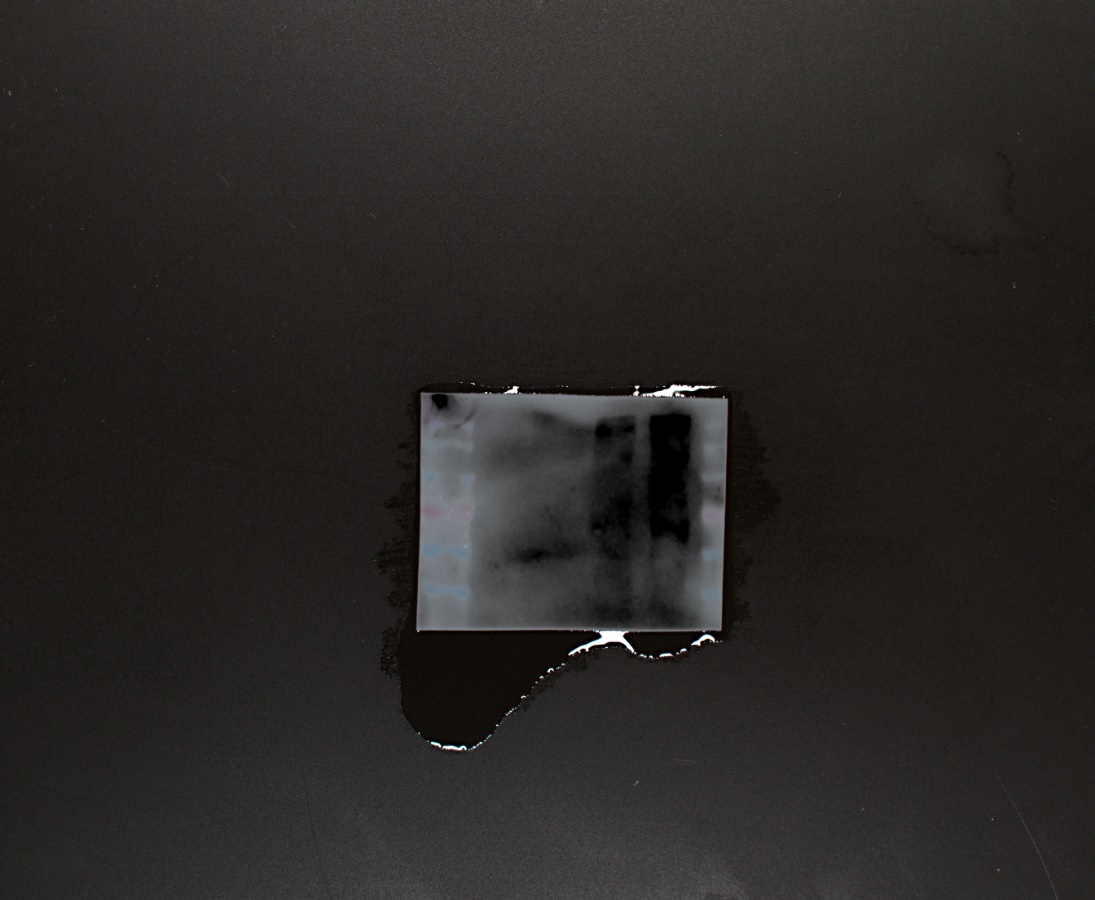




**Kda**

**180**

**70**

**Ubiquitin**


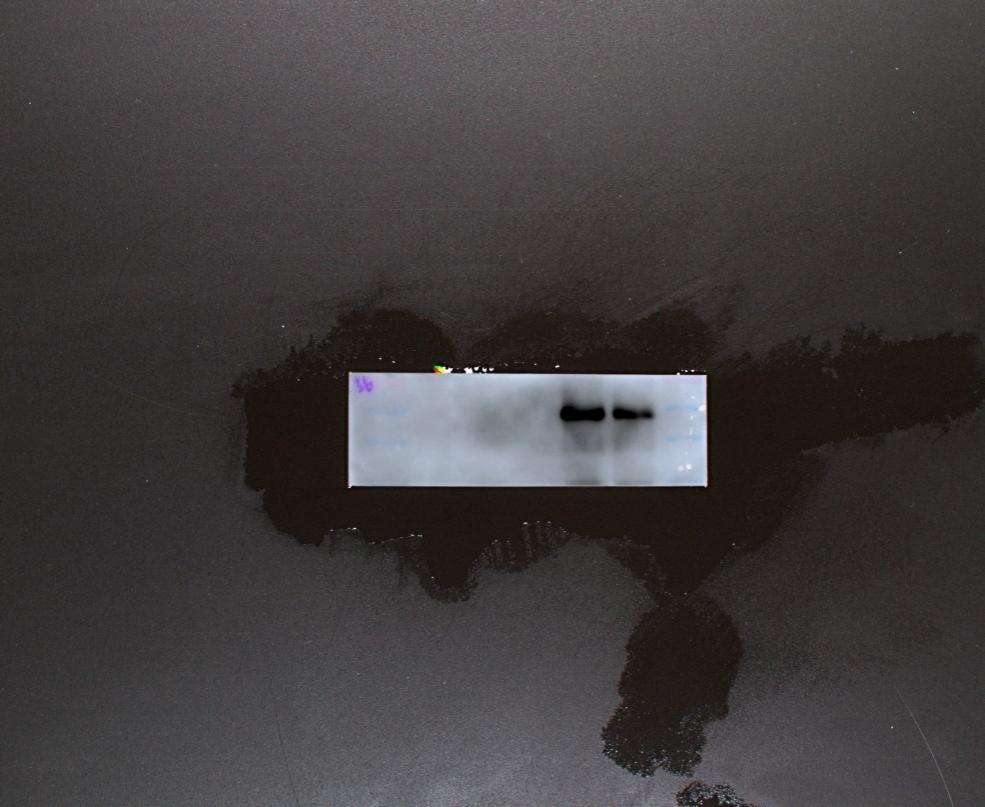

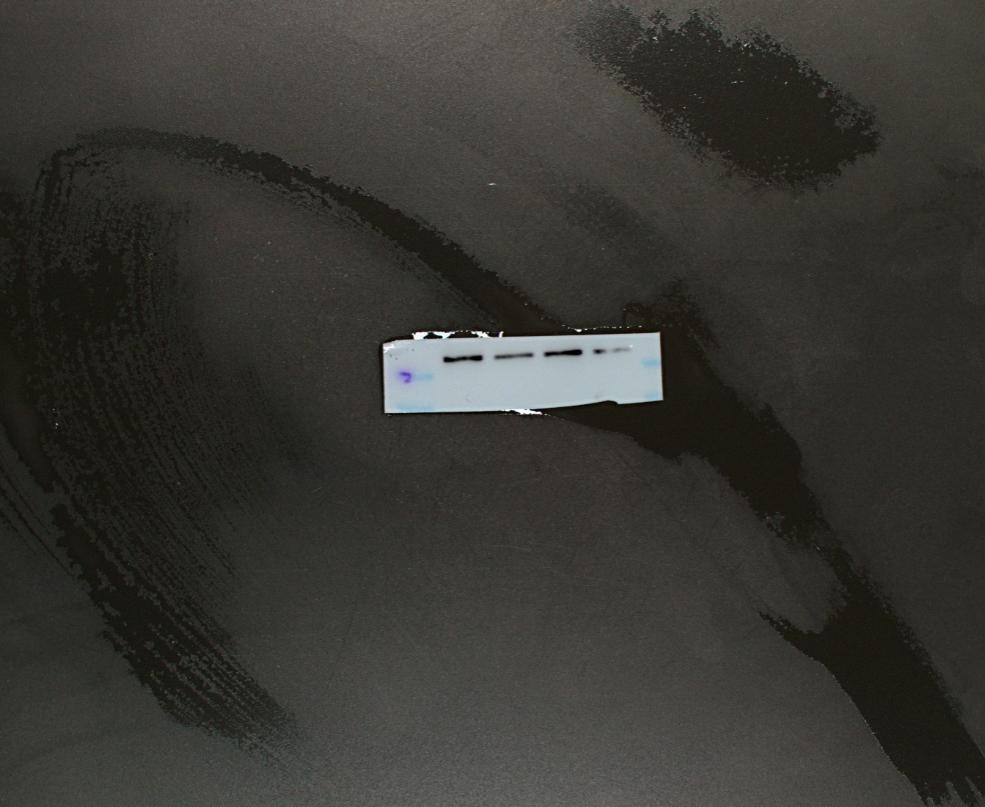


**Kda**

**34**

**25**

**Kda**

**42**

**35**

**SIRT6 CAPZA1**





**Kda**

**43**

**35**

**β-actin**

**Fig 9a**







**Kda**

**180**

**119**

**Kda**

**42**

**35**

**USP48 SIRT6**







**Kda**

**43**

**Kda**

**33**

**CAPZA1 β-actin**

**Fig 9b**







**Kda**

**42**

**35**

**Kda**

**180**

**119**

**USP48 SIRT6**





 **CAPZA1 β-actin**

**Kda**

**43**

**33**

**Kda**

**55**

**43**

**Fig 9d,e**







**Kda**

**42**

**35**

**Kda**

**119**

**70**

**USP48 SIRT6**







**Kda**

**119**

**70**

**Kda**

**43**

**35**

**β-actin USP48**







**Kda**

**43**

**Kda**

**42**

**35**

**SIRT6 β-actin**

**Fig 9f**







**Kda**

**42**

**35**

**Kda**

**119**

**70**

**USP48 SIRT6**





**Kda**

**43**

**β-actin**

**Fig 9g**







**Kda**

**42**

**35**

**Kda**

**119**

**70**

**SIRT6 USP48**





**Kda**

**43**

**β-actin**

**Fig 9h**







**Kda**

**119**

**70**

**Kda**

**42**

**35**

**USP48 SIRT6**





**Kda**

**43**

**35**

**β-actin**

**Fig 9i**







**Kda**

**119**

**Kda**

**180**

**55**

**IP Ubiquitin IP USP48**







**Kda**

**42**

**35**

**Kda**

**33**

**25**

**IP CAPZA1 IP SIRT6**







**Kda**

**33**

**25**

**Kda**

**42**

**IB SIRT6 IB CAPZA1**







**Kda**

**55**

**43**

**Kda**

**119**

**70**

**IB USP48 β-actin**

**Fig 9j**







**Kda**

**119**

**Kda**

**180**

**70**

**IP Ubiquitin IP USP48**







**Kda**

**42**

**35**

**Kda**

**33**

**25**

**IP CAPZA1 IP SIRT6**







**Kda**

**33**

**25**

**Kda**

**42**

**35**

**IB SIRT6 IB CAPZA1**







**Kda**

**55**

**43**

**Kda**

**33**

**25**

**IB CAPZA1 β-actin**

**Fig S2a**







**Kda**

**120**

**Kda**

**43**

**34**

**ACLY CDK4**





**Kda**

**43**

**β-actin**

**Fig S2f**





**Kda**

**120**

**Kda**

**130**

**N-caderin E-caderin**

**Kda**

**43**

**35**

**Kda**

**150**

**ZEB2 β-actin**

**Kda**

**120**

**Kda**

**130**

**N-caderin E-caderin**

**Kda**

**43**

**35**

**Kda**

**150**

**ZEB2 β-actin**

**Fig.S3b**

**Kda**

**43**

**33**

**Kda**

**43**

**CAPZA1 β-actin**

**Fig S3e**

**Kda**

**33**

**Kda**

**55**

**43**

**CAPZA1 β-actin**

**Fig S4a**

**Kda**

**55**

**43**

**Kda**

**43**

**33**

**CAPZA1 β-actin**

**Kda**

**43**

**Kda**

**33**

**CAPZA1 β-actin**

**Fig S7a**

**Kda**

**55**

**42**

**Kda**

**43**

**SIRT6 β-actin**

**Kda**

**42**

**35**

**Kda**

**43**

**SIRT6 β-actin**

**Fig S8f**

**Kda**

**110**

**70**

**Kda**

**40**

**ACAT2 NRF2**

**Kda**

**43**

**Kda**

**40**

**β-actin ACAT2**

**Kda**

**43**

**Kda**

**110**

**70**

**NRF2 β-actin**

**Fig S9b**

**Kda**

**40**

**Kda**

**42**

**35**

**SIRT6 ACAT2**

**Kda**

**43**

**35**

**β-actin**
